# From observable fermentation data to hidden cell states: A modeling study of a mixotrophic *Clostridium* coculture under perfusion mode

**DOI:** 10.1371/journal.pcbi.1014759

**Published:** 2026-09-28

**Authors:** Juhyeon Kim, Hangjun Cho, Jin Hong Mok, Hyeongmin Seo, Joseph Sang-Il Kwon

**Affiliations:** 1 Artie McFerrin Department of Chemical Engineering, Texas A&M University, College Station, Texas, United States of America; 2 Texas A&M Energy Institute, Texas A&M University, College Station, Texas, United States of America; 3 William G. Lowrie Department of Chemical and Biomolecular Engineering, The Ohio State University, Columbus, Ohio, United States of America; 4 Department of Mathematics Education, Kongju National University, Kongju, Republic of Korea; 5 Department of Food Science and Biotechnology, Dongguk University–Seoul, Goyang-si, Gyeonggi-do, Republic of Korea; 6 Department of Chemical and Biochemical Engineering, The University of Iowa, Iowa City, Iowa, United States of America; University of Nebraska-Lincoln, UNITED STATES OF AMERICA

## Abstract

Microbial cocultures exhibit complex population dynamics that are difficult to interpret because internal physiological states are only partially observable. In particular, active and dormant cell states can influence system-level behavior but are rarely resolved from standard fermentation measurements. In this study, we present a hybrid modeling framework that combines structured population-state reconstruction with sparse identification of nonlinear dynamics (SINDy) to analyze a *Clostridium acetobutylicum*-*Clostridium ljungdahlii* coculture under perfusion mode. The framework estimates active $C_{ac}$ and $C_{lj}$ biomass-equivalent trajectories from observable biomass and activity measurements, reconstructs dormant populations as model-constrained latent states, and uses these states to identify extracellular metabolite dynamics. After accounting for first-principles perfusion transport, SINDy identified sparse biological reaction terms associated with organic acid turnover, solvent formation, and acetone-to-isopropanol conversion. The resulting model captured active-biomass and metabolite trajectories and suggested that the coculture dynamics are consistent with acid-associated state transitions and sequential metabolic exchange. This framework provides a transparent strategy for interpreting partially observed microbial cocultures while explicitly treating dormant biomass as a latent reconstructed state.

## Author summary

Synthetic microbial consortia are rapidly emerging as a promising paradigm in modern biotechnology because different organisms can work together to perform complicated tasks. However, elucidating their behavior remains challenging. In particular, important cellular states, such as whether they are actively growing or temporarily inactive, cannot be directly measured. This limits the interpretation of experimental data and the design of effective operating strategies. In this work, we present a modeling approach to reconstruct these hidden aspects of the microbial coculture systems. We first use a mechanistic model to estimate how the total biomass is distributed between active and inactive cells. We then combine this with a data-driven method to identify the key relationships associated with system behavior. Using a mixotrophic *Clostridium* coculture in continuous perfusion fermentation, we demonstrate that its dynamics are consistent with shifts in cell activity and cooperative interactions between the two species. By mathematically resolving the aggregate biomass into active and dormant subpopulations, we obtained model-supported insights suggesting that the complex dynamics of cocultures arise from a nuanced interplay between stress-induced dormancy and sequential metabolic exchanges, rather than simple growth dynamics. Overall, this approach provides a practical way to better understand and improve various microbial coculture systems.

## Introduction

The production of biofuels and biochemicals from renewable feedstocks has attracted significant attention as a sustainable alternative to petrochemical processes [1–3]. Industrial biotechnology is revisiting anaerobic fermentation routes to produce drop-in fuels and commodity solvents, as solventogenic Clostridia can achieve high product titers and use diverse feedstocks [4–6]. *Clostridium acetobutylicum* is a well-established ABE (acetone, butanol, ethanol) producer utilizing carbohydrates. However, the inherent limitation in carbon yield during ABE fermentation arises since a significant portion of sugar carbon is released as CO_2_ during decarboxylation reactions in solventogenesis [7], leading to observed total product yields that are substantially below the theoretical maximum from the carbon balance [8,9]. To overcome this constraint, the mixotrophic coculture *Clostridium acetobutylicum* ($C_{ac}$) with the acetogen *Clostridium ljungdahlii* ($C_{lj}$) has been proposed [10,11]. This synergistic system can simultaneously utilize organic substrates and gases, enhancing total carbon recovery through interspecies cross-feeding and metabolite re-assimilation.

Despite these advantages, the optimization of such coculture systems is fundamentally constrained by limited observability of cell physiological states including their populations [12]. In addition, *Clostridium* organisms exhibit multiple cellular states such as actively growing, resting (metabolically active but non-growing), sporulating, and spored states [13,14]. Therefore, $C_{ac}$ and $C_{lj}$ could present as either metabolically active or dormant populations within the fermentation broth. However, cell growth in heterogeneous microbial cocultures is often assessed using aggregate parameters such as total optical density or overall dry cell weight. The dynamic and complex cellular states in coculture systems often complicate fermentation control. Dormancy is defined here as a reversible state of reduced metabolic activity rather than cell death or complete metabolic shutdown [15,16]. Accordingly, cells may transition between active and dormant physiological states in response to environmental stress and metabolic conditions. Some dormant cells (*e.g.*, resting cells or non-growing cells) can uptake sugars and convert to electron-balanced products to some extent. In practical fermentation monitoring, however, only total biomass or active fractions can be quantified, while the distribution of dormant subpopulations remains experimentally inaccessible. This observability gap obscures the true physiological state of the consortia, making it difficult to develop accurate control strategies using standard unstructured kinetic models.

To reconstruct these hidden physiological states and identify metabolic relationships, a novel modeling approach is required. Notably, first-principles models provide a structural basis for cell growth and formulate the rate equations for each reaction [17,18]. For example, kinetic models based on metabolic reaction networks, such as the one developed by Shinto *et al.*, utilize a set of ordinary differential equations to describe the flux transitions in ABE fermentation [19]. Similarly, genome-scale metabolic models, exemplified by Nagarajan *et al.*, provide a comprehensive map of the metabolic potential of $C_{lj}$ [20]. Efforts have also been made to extend these frameworks to microbial consortia. For the specific $C_{ac}$-$C_{lj}$ pair, Foster *et al.* developed a kinetic model incorporating interspecies cell fusion and metabolite exchange [11]. Related fermentation and biosystem studies have also used multiscale modeling frameworks to connect process-level conditions with product-distribution properties [21]. In addition, broader modeling strategies for other coculture systems have employed dynamic flux balance analysis (dFBA) to predict interspecies interactions [22]. However, whether kinetic or constraint-based, these approaches rely on pre-defined kinetic expressions (*e.g.*, Michaelis-Menten types) that are structurally rigid. This rigidity limits their ability to capture unobservable states or transient physiological shifts, which often govern the system’s robustness under inhibitory stress, without requiring extensive parameter tuning or ad-hoc assumptions.

Conversely, purely data-driven strategies have emerged to bypass the complexity of kinetic formulation. In the broader context of bioprocess engineering, machine learning algorithms such as Artificial Neural Networks (ANN) and Long Short-Term Memory (LSTM) networks have been widely adopted to predict biomass growth and product formation from time-series data [23–28]. Narrowing down to the specific system of interest, Roell *et al.* demonstrated the efficacy of ensemble methods, such as random forests, in predicting complex syngas fermentation outcomes with high accuracy [29,30]. Furthermore, in the realm of microbial consortia, Treloar *et al.* reported the application of deep reinforcement learning to control coculture population ratios without requiring prior biological knowledge [31]. However, despite their predictive power, these models operate primarily as ‘black-boxes’. By prioritizing predictive accuracy over mechanistic transparency, they offer limited insight into the underlying physiological transitions or the causal relationships associated with metabolic changes. Consequently, they remain insufficient for uncovering the survival strategies of the consortia, creating a critical need for a strategy that bridges the gap between mechanistic interpretability and data-driven flexibility [32–34].

To incorporate physical laws directly from data while maintaining interpretability, sparse regression methods have gained prominence as an alternative to the existing black-box algorithms. Sparse representations of biological dynamical systems have been successfully employed in numerous studies, suggesting that macroscopic reaction dynamics often admit parsimonious descriptions despite underlying molecular complexity [35–37]. Among these, the Sparse Identification of Nonlinear Dynamics (SINDy) [38] enables the discovery of parsimonious governing equations by selecting the most relevant terms from a candidate function library [39]. This approach has been successfully applied to diverse physical systems, ranging from chemical oscillations [40] to reactor modeling [41]. However, a critical challenge in applying SINDy to biological systems is its reliance on full state measurements. It struggles when key physiological variables become noisy [42] or unobservable [40]. In this study, therefore, we address this limitation by establishing a physiologically informed data-driven strategy that integrates domain knowledge with sparse identification. Rather than applying SINDy directly to raw data, we first reconstruct the hidden metabolic states to enable accurate structure discovery. Our framework specifically aims to: (i) mathematically resolve the aggregate biomass into active and dormant fractions to resolve the hidden physiological transitions; (ii) construct a physiologically informed feature library based on these reconstructed states; and (iii) employ SINDy to identify the unknown kinetic structures associated with substrate uptake and solvent production. Through this stepwise approach, we provide a model-supported interpretation of survival-related population and metabolic trends of the coculture, providing a rational basis for designing robust mixotrophic fermentation processes.

## Materials and methods

The overall workflow is summarized in Fig 1, including experimental measurements, state reconstruction, and SINDy-based model identification.

**Fig 1:**
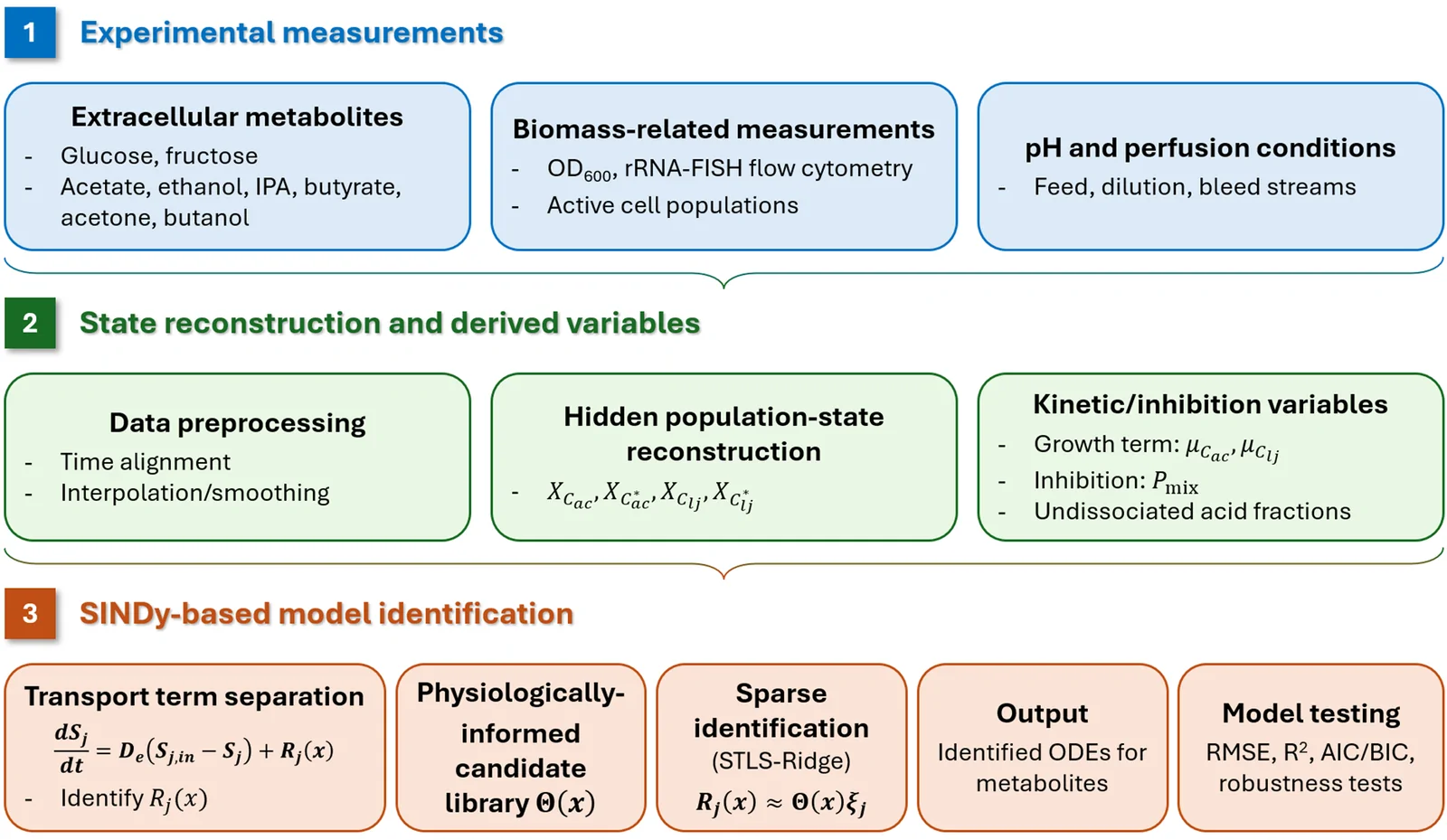
Overview of the proposed modeling framework.

A genetically engineered *Clostridium acetobutylicum* strain CACas9 Δhbd harboring p95ace02atoB plasmid ($C_{ac}$) and *C. ljungdahlii* harboring p100ptaHALO ($C_{lj}$) were used for coculture as previously described [43]. CACas9 Δhbd lacks butyrate formation by complete deletion of 3-hydroxybutyryl-CoA dehydrogenase gene (*hbd*) [44]. 2xYTG liquid or solid media supplemented with 30 mM of sodium butyrate and 40 μg/mL clarithromycin were used to prepare $C_{ac}$ seed culture [43]. For $C_{lj}$, a frozen glycerol stock was inoculated into 20 *mL* of YTF medium supplemented with 3.5 *g*/*L* arginine and 25 *g*/*L* MES (YTAF-MES) with 100 μg/mL clarithromycin in a 160 *mL* serum bottle [43]. The serum bottle was flushed for 2 *min* with an 80% H_2_, 20% CO_2_ gas mixture and pressurized to 20 psig. The cells were grown for 24 hours at $37^{\circ }C$ in a shaking incubator at 90 rpm. All subsequent culturing steps were performed in Turbo CGM growth medium supplemented with 30 *mM* sodium butyrate (“Turbo CGMB”) with 100 μg/mL clarithromycin. The experimental data shown here were retrieved from Willis *et al.* [43]. The dataset used in this modeling study was obtained from a single perfusion bioreactor operation and did not include independent biological replicate runs.

### Bioreactor setup and operation

The schematic diagram is shown in Fig 2a. Perfusion cocultures were conducted in a bioreactor system as previously described [43]. The reactor vessel was maintained at a 1.2 *L* working volume, and the packed column contained 2.4 *L*, resulting in a total system volume of 3.6 *L*. An anaerobic environment was established by sparging an 80/20 H_2_/CO_2_ gas mixture through the packed bed at 3.6 *L*/*h*. Reactor pH was controlled by automated addition of 4 *M* NaOH. To initiate the perfusion coculture, 80 *mL* of *C. acetobutylicum* pre-culture was inoculated into the perfusion reactor containing Turbo CGMB medium supplemented with 60 *g*/*L* glucose and 100 μg/mL clarithromycin. After inoculation, perfusion was started with TurboCGMB containing 20 *g*/*L* glucose, fed and withdrawn at 4.86 *mL*/*min* (approximately two reactor volumes per day). Cell retention was achieved usin*g* a GE Healthcare hollow-fiber cartridge (0.1 μm pore size, 850 *cm*^2^ surface area; CFP-1-E-4X2MA). After 70 h of fermentation, 120 *mL* of concentrated *C. ljungdahlii* pre-culture was introduced into the reactor system. The reactor was operated in complete cell-retention mode until 100 h, after which a 10% bleed was initiated.

**Fig 2:**
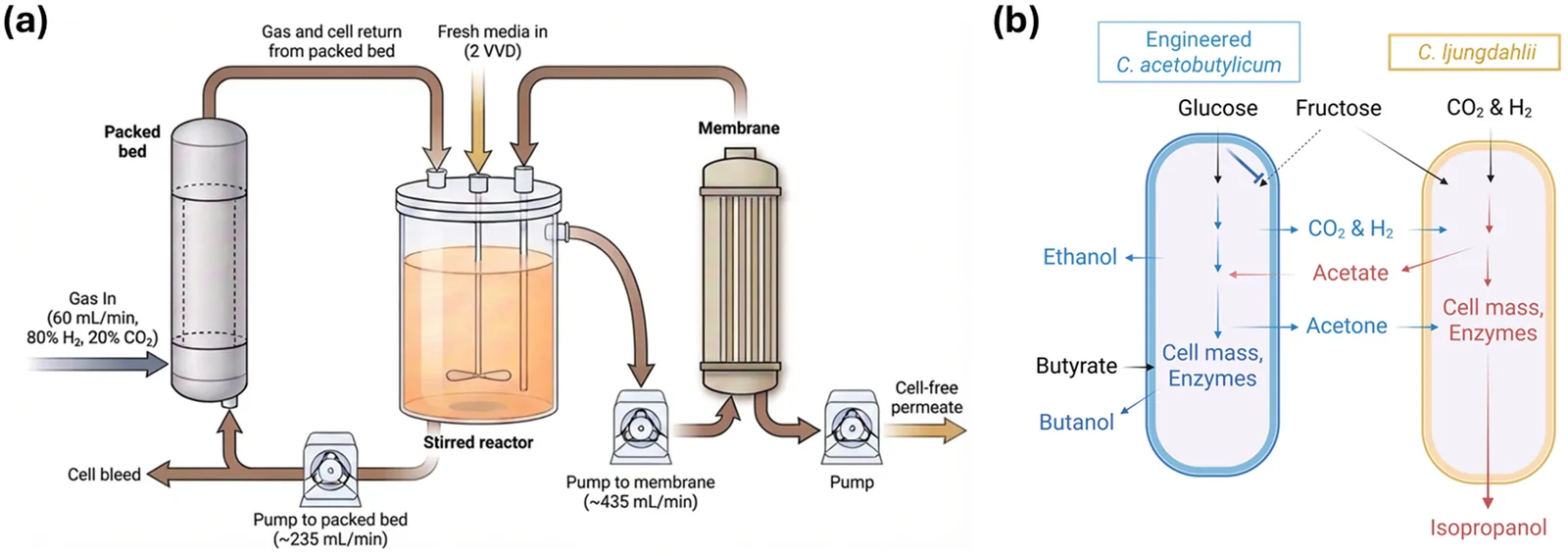
Visualization of the system configuration. A: A schematic representation of the fermentation system. B: A visualization of interspecies reaction network.

### Analytical methods

#### High performance liquid chromatography (HPLC).

Metabolite composition of the culture media was analyzed as previously described [45,46]. HPLC (Agilent) was performed using an Aminex HPX-87H (Bio-Rad) with 5 *mM* H_2_SO_4_ as the mobile phase at a flow rate of 0.5 *mL*/*min* at $15^{\circ }C$. The metabolites were detected and quantified using a refractive index detector.

#### Ribosomal RNA-fluorescence *in situ* hybridization (rRNA-FISH).

To analyze population dynamics of the $C_{ac}$-$C_{lj}$ coculture, rRNA-FISH was performed using the probes specific for $C_{ac}$ and $C_{lj}$ [47]. After washing culture samples twice in filter-sterilized (0.2 μm) ice-cold 1× PBS (Gibco, pH 7.4), the pellet was resuspended thoroughly in ice-cold 1× PBS and fixed by 1:1 dilution in ice-cold absolute ethanol. The fixed samples were stored at $-20^{\circ }C$ for up to a month. After removing the supernatant, pellets were dried at $46^{\circ }C$ for 5–15 *min* to remove residual ethanol. Pellets were resuspended in 75 μL hybridization buffer (0.9 *M* NaCl, 0.02 *M* Tris-HCl pH 7.0, 20% formamide, 0.01% SDS, 1 μM probe) and incubated at $46^{\circ }C$ for 5 h. Cells were then pelleted by centrifugation at 10,000 xg for 10 *min*, and the supernatant was discarded into formamide waste. Pellets were washed twice in 500 μL prewarmed wash buffer (0.215 *M* NaCl, 5 μM EDTA, 0.02 *M* Tris-HCl pH 7.0, 0.01% SDS) at $48^{\circ }C$ for 20 *min* followed by a final wash and resuspension in 1 *mL* ice-cold 1× PBS.

#### Flow cytometry.

Flow cytometry was performed using a CytoFLEX S analyzer (Beckman Coulter Life Sciences, Indianapolis, IN, USA) [47]. Data were acquired and processed with CytExpert software (version 2.4.0.28; Beckman Coulter). Cell suspensions were diluted in ice-cold 1× PBS to approximately 10^5^ cells/mL (OD_600_~ 0.1). The sample flow rate (10–60 μL/min) was adjusted to achieve ~1,000 events per second while maintaining an abort rate < 7%. For each sample, 5 μL was analyzed. Fluorescence was collected using the following configurations: ClosAcet and Janelia 549, yellow laser with 585/42 BPF; ClosLjun, Janelia 646, and Deep Red, red laser with 712/25 BPF. Avalanche photodiode gains were set to 1,000 and 850 for ClosAcet, and ClosLjun, respectively, and standard quality-control gain settings were used for HaloTag ligands and Deep Red. Fluorescence intensity was recorded as the integrated area of the emission pulse, and the population median was used as the summary statistic for each channel. For population-fraction estimation, fluorescence thresholds for rRNA-FISH-labeled cells were established based on the distribution observed in negative control experiments. Cells exhibiting fluorescence signals above the corresponding threshold were classified as active cells for each species. The resulting active fractions of $C_{ac}$ and $C_{lj}$ were multiplied by the OD_600_-derived total biomass-equivalent concentration to estimate $X_{C_{ac}}$ and $X_{C_{lj}}$, respectively. Unlabeled cells were not treated as independently measurable values, and $X_{C_{ac}^{*}}$ and $X_{C_{lj}^{*}}$ were reconstructed as latent states in the structured population model as discussed below.

## Model formulation

### Cell population estimation

An overview of the overall reaction pathway is shown in Fig 2b. In this work, the microbial populations within the coculture system are categorized into four distinct functional states: active $C_{ac}$, active $C_{lj}$, dormant $C_{ac}$ (denoted as $C_{ac}^{*}$ hereafter), and dormant $C_{lj}$ ($C_{lj}^{*}$), which is consistent with previous studies where populations are partitioned into actively growing and dormant subpopulations connected by reversible transitions [48,49].

Within this framework, all four categories are assumed to remain metabolically active at the enzymatic level. Nevertheless, their physiological roles differ with respect to growth and substrate utilization. Active cells are capable of carbohydrate uptake and proliferation, whereas dormant cells are assumed to have ceased growth and do not actively consume sugars (*i.e.*, glucose and fructose). Instead, the dormant subpopulation is supported primarily by low-level maintenance metabolism [50].

While the concentration of sugar-consuming active cells can be experimentally tracked via quantitative analysis, monitoring the dormant population presents a significant challenge. Despite their inability to replicate or consume sugar, dormant cells continue to engage in metabolic activities, thereby influencing the overall system behavior. The lack of observability for the dormant fraction complicates the quantitative interpretation of the coculture system. Consequently, to successfully predict carbohydrate consumption and accurately trace the concentration profiles of associated metabolites, it is imperative to rigorously track the dynamics of both active and dormant populations.

Note that ‘dormant’ here is used as an effective latent-state descriptor for viable, non-growing cells that retain low-level metabolic activity. This definition overlaps with resting or maintenance-state cells, and the present data do not allow these non-growing phenotypes to be resolved separately. Therefore, the dormant compartment should be interpreted as a lumped non-growing but metabolically retained state. Alternative terms such as ‘resting’ or ‘maintenance-state’ would lead to an equivalent formulation under the same assumptions of reversibility and low-level metabolic activity.

The temporal evolution of $C_{ac}$ and $C_{lj}$ biomasses in the reactor is governed by five primary mechanisms: specific cell growth, inactivation (transition from active to dormant), reactivation (transition from dormant to active), cell death, and physical removal via bleeding. The dynamic mass balances for the active and dormant cell populations (*X*) for each species are described by the following equations:


$$\begin{array}{cc} \frac{dX_{C_{ac}}}{dt} & =(\mu_{C_{ac}}-k_{i,C_{ac}}-k_{d,C_{ac}}-b)X_{C_{ac}}+k_{r,C_{ac}}X_{C_{ac}^{*}}\\ \frac{dX_{C_{lj}}}{dt} & =(\mu_{C_{lj}}-k_{i,C_{lj}}-k_{d,C_{lj}}-b)X_{C_{lj}}+k_{r,C_{lj}}X_{C_{lj}^{*}} \end{array}$$ (1)

$$\begin{array}{cc} \frac{dX_{C_{ac}^{*}}}{dt} & =k_{i,C_{ac}}X_{C_{ac}}-(k_{d,C_{ac}}+b+k_{r,C_{ac}})X_{C_{ac}^{*}}\\ \frac{dX_{C_{lj}^{*}}}{dt} & =k_{i,C_{lj}}X_{C_{lj}}-(k_{d,C_{lj}}+b+k_{r,C_{lj}})X_{C_{lj}^{*}} \end{array}$$ (2)

where μ is the specific growth rate, $k_{i}$ is the specific inactivation rate, $k_{d}$ is the specific cell death rate, $k_{r}$ is the specific reactivation rate, and *b* is the dilution rate due to the bleed stream in *h*^–1^. Comparing Eqs 1–2, only active cells, $C_{ac}$ and $C_{lj}$, can grow over time. The population variables in Eqs 1–2 are expressed as biomass-equivalent concentrations. The total biomass scale was obtained from OD_600_ measurements using an OD-to-biomass conversion factor of $0.50\;gDCW\;L^{-1}\;OD_{600}^{-1}$, while rRNA-FISH/flow cytometry measurements were used to constrain the active populations of $C_{ac}$ and $C_{lj}$. Thus, $X_{C_{ac}}$ and $X_{C_{lj}}$ represent experimentally constrained active biomass-equivalent concentrations, whereas $X_{C_{ac}^{*}}$ and $X_{C_{lj}^{*}}$ represent model-reconstructed dormant biomass-equivalent latent states on the same scale.

To mathematically describe the physiological state of the cells, the specific growth rates (μ) were formulated using a multiplicative kinetic model. This model accounts for substrate limitation via Monod kinetics [51] and the cumulative inhibitory effects of metabolic products using an exponential inhibition term [52].

Growth of $C_{ac}$ is modeled as growing exclusively on glucose. Its specific growth rate, $\mu_{C_{ac}}$, follows Monod kinetics with respect to glucose concentration, modulated by the product inhibition term:


$$\begin{array}{cc} \mu_{C_{ac}} & =\mu_{max,C_{ac}}\frac{S_{glu}}{K_{glu}+S_{glu}}\exp(-P_{mix,C_{ac}}) \end{array}$$ (3)

where $\mu_{max,C_{ac}}$ is the maximum specific growth rates of $C_{ac}$, $S_{glu}$ is the glucose concentration, and $K_{glu}$ is the half-saturation constant for glucose. Particularly, $\exp\left(-P_{mix,C_{ac}}\right)$ accounts for the inhibition caused by solvents and acid accumulation. Note that the concentrations are in *mM* and the growth rates are in *h*^–1^.

Growth of $C_{lj}$ is capable of mixotrophic growth, utilizing both organic substrates (fructose) and gaseous substrates (H_2_ and CO_2_) via the Wood-Ljungdahl pathway [53]. Accordingly, the specific growth rate for $C_{lj}$, $\mu_{C_{lj}}$, is described as follows:


$$\begin{array}{cc} \mu_{Clj}= & \left[\mu_{max,fru}\left(\frac{S_{fru}}{K_{fru}+S_{fru}}\right)+\mu_{max,gas}\left(\frac{S_{H_{2}}}{K_{H_{2}}+S_{H_{2}}}\right)\left(\frac{S_{CO_{2}}}{K_{CO_{2}}+S_{CO_{2}}}\right)\right]\\ & \times \exp(-P_{mix,C_{lj}}) \end{array}$$ (4)

where $\mu_{max,fru}$ and $\mu_{max,gas}$ stand for the maximum specific growth rates on fructose and gases, respectively. $S_{fru}$, $S_{H_{2}}$, $S_{CO_{2}}$ correspond to the concentration of fructose and dissolved gases. The multiplicative term for gases within the autotrophic component reflects the stoichiometric requirement for both H_2_ and CO_2_ in the Wood-Ljungdahl pathway, and $\exp\left(-P_{mix,C_{lj}}\right)$ represents the inhibition term.

To quantify the cumulative toxicity exerted by the accumulation of metabolic end-products, a dimensionless product inhibition term, $P_{mix}$, was introduced. The fermentation broth accumulates various byproducts, particularly solvents [6] and organic acids [54], which can create a toxic environment that can hinder cell growth and accelerate inactivation. Based on the assumption that the inhibitory effects of multiple toxic compounds are additive within the exponential inhibition framework, $P_{mix}$ is defined as the sum of the concentrations of individual metabolites normalized by their respective inhibition constants ($K_{I}$) [51,52].

For solvents (neutral species), the effective concentration is equal to the total concentration. However, for organic acids (acetate and butyrate), the toxicity is primarily attributed to the undissociated form [55]. Their influx and intracellular dissociation impose proton stress and energetic burden for pH homeostasis, making inhibition correlate with the undissociated acid fraction estimated by the Henderson-Hasselbalch equation [54] as follows:


$$\begin{array}{cc} S_{A,\text{eff}} & =\frac{S_{A,\text{measured}}}{1+10^{pH-pK_{a,A}}} \end{array}$$ (5)

where A∈ {*ace* (Acetate), *buty* (Butyrate)}, $S_{A,\text{measured}}$ is the measured total concentration of each organic acid (*mM*), *pH* is the current culture pH, and $pK_{a,A}$ is the acid dissociation constant (*e.g.*, 4.76 for acetate and 4.82 for butyrate).

Accordingly, the composite inhibition term $P_{mix}$ is formulated as:


$$\begin{array}{cc} P_{mix,C_{ac}} & =\sum_{j}\frac{S_{j,\text{eff}}}{K_{I,j,C_{ac}}}\\ P_{mix,C_{lj}} & =\sum_{j}\frac{S_{j,\text{eff}}}{K_{I,j,C_{lj}}} \end{array}$$ (6)

where $S_{j,\text{eff}}$ represents the effective concentrations of metabolites *j* (mM), where j∈ {*ace* (Acetate), *acn* (Acetone), *EtOH* (Ethanol), *IPA* (Isopropanol), *buty* (Butyrate), *BuOH* (Butanol)}. $K_{I}$ is used as the inhibition constant, showing different values for each cell and metabolite. Solvents, particularly butanol, disrupt the phospholipid bilayer of the cell membrane, leading to leakage of intracellular components. In contrast, the undissociated organic acids calculated via Eq 5 collapse the transmembrane proton gradient upon entering the cytoplasm and dissociating, thereby depleting the ATP pool required for growth. This formulation allows the model to dynamically account for the pH-dependent toxicity of weak acids.

The specific inactivation rate, $k_{i}$, represents the physiological transition from the active to the dormant state. It is postulated that this transition is driven primarily by metabolic stress resulting from product accumulation. Consequently, $k_{i}$ is modeled as the sum of a stress-induced component linearly proportional to the appropriate metabolite concentrations, using the Hill equation [56].


$$\begin{array}{cc} k_{i,C_{ac}} & =k_{i,ace}\frac{S_{ace}^{2}}{K_{ace}^{2}+S_{ace}^{2}}+k_{i,buty}\frac{S_{buty}^{2}}{K_{buty}^{2}+S_{buty}^{2}}\\ k_{i,C_{lj}} & =k_{i,IPA}\frac{S_{IPA}^{2}}{K_{IPA}^{2}+S_{IPA}^{2}} \end{array}$$ (7)

where the subscripts *ace*, *buty*, and *IPA* stand for acetate, butyrate, and IPA, respectively. The linear formulation captures the assumption that higher concentrations of toxic metabolites directly precipitate the cessation of cell replication and sugar consumption. The Hill coefficient was fixed at *n* = 2 as a phenomenological choice to capture a threshold-like, saturating stress response while avoiding additional non-identifiability from fitting the exponent itself.

The specific cell death rate ($k_{d}$) is modeled as a first-order process whose specific rate coefficient depends linearly on the concentrations of inhibitory metabolites within the experimentally observed range.


$$\begin{array}{cc} k_{d,C_{ac}} & =k_{d0,C_{ac}}+D_{C_{ac},IPA}S_{IPA}+D_{C_{ac},buty}S_{buty}\\ k_{d,C_{lj}} & =k_{d0,C_{lj}}+D_{C_{lj},IPA}S_{IPA} \end{array}$$ (8)

where $k_{d0}$ represents the basal turnover of cells that undergo cell death spontaneously, and *D* is a proportionality constant. In this framework, cell death is distinguished from inactivation. Cell inactivation denotes the reversible transition from active cells to dormant cells, and reactivation denotes the reverse transition from dormant cells back to active cells. By contrast, cell death is treated as an irreversible sink from the viable population. Cells undergoing death are removed from the modeled active or dormant populations and are not eligible for reactivation. The death term therefore acts as a sink from both active and dormant populations in Eqs 1– 2. A small basal turnover (see Table 1) is accounted for by a constant offset.

**Table 1:** Kinetic parameters and inhibition constants used in the coculture model to fit the structured population model to the experimental dataset used in this study.

Parameter	Symbol	Unit	$C_{ac}$ Value	$C_{lj}$ Value
**Growth Kinetics (Eqs 3–4)**				
Maximum specific growth rate (Glucose/Fructose)	$\mu_{max}$	*h* ^–1^	0.327	0.225
Maximum specific growth rate (Gas)	$\mu_{max,gas}$	*h* ^–1^	–	0.105
Half-saturation constant (Glucose)	$K_{Glu}$	*mM*	5.000	–
Half-saturation constant (Fructose)	$K_{Fru}$	*mM*	–	0.100
Half-saturation constant (*H*_2_)	$K_{H2}$	*mM*	–	0.100
Half-saturation constant (*CO*_2_)	$K_{CO2}$	*mM*	–	0.060
**Inactivation Kinetics (Eq 7)**				
Stress sensitivity coefficient (acetate)	$k_{i,ace}$	*h* ^–1^	0.0474	–
Stress sensitivity coefficient (butyrate)	$k_{i,buty}$	*h* ^–1^	0.0059	–
Stress sensitivity coefficient (isopropanol)	$k_{i,IPA}$	*h* ^–1^	–	0.0051
Half-saturation constant (acetate)	$K_{ace}$	*h* ^–1^	6.23	–
Half-saturation constant (butyrate)	$K_{buty}$	*h* ^–1^	25.0	–
Half-saturation constant (isopropanol)	*K_IPA_*	*h* ^–1^	–	100.
**Death Kinetics (Eq 8)**				
Basal turnover	$k_{d0}$	*h* ^–1^	0.0021	0.203
Linear coefficients (isopropanol)	$D_{IPA}$	*h* ^–1^	0.0032	0.0407
Linear coefficients (butyrate)	$D_{buty}$	*h* ^–1^	1.22E-5	–
**Product Inhibition Constants ($K_{I}$)**				
Acetate	$K_{I,Ace}$	*mM*	83.3	100.
Butyrate	$K_{I,Buty}$	*mM*	20.0	18.2
Ethanol	$K_{I,EtOH}$	*mM*	133.	80.0
Isopropanol	$K_{I,IPA}$	*mM*	133.	88.9
Acetone	$K_{I,Acn}$	*mM*	100.	100.
Butanol	*K_I,BuOH_*	*mM*	5.00	6.25
**Reactivation (Eq 9)**				
Rate coefficient (Acid)	$k_{r}^{acid}$	*h* ^–1^	1.133	3.155
Rate coefficient (Sugar)	$k_{r}^{sugar}$	*h* ^–1^	1.498	2.997
Acid relief scale	$K_{a}$	*mM*	0.995	0.995
Sugar sensitivity parameter	$K_{s}$	*mM*	5.100	5.100

The reactivation rates are defined using saturating functional forms, which have been used in active-dormant microbial models [57], to phenomenologically represent the combined effects of acid stress relief and restoration of metabolic homeostasis under reduced inhibitory conditions as follows:


$$\begin{array}{cc} k_{r,C_{ac}} & =k_{r,C_{ac}}^{\text{acid}}\frac{1}{1+\frac{\sum S_{\text{acid,eff}}}{K_{a}}}+k_{r,C_{ac}}^{\text{sugar}}\frac{\sum S_{\text{sugar}}}{K_{s}+\sum S_{\text{sugar}}}\\ k_{r,C_{lj}} & =k_{r,C_{lj}}^{\text{acid}}\frac{1}{1+\frac{\sum S_{\text{acid,eff}}}{K_{a}}}+k_{r,C_{lj}}^{\text{sugar}}\frac{\sum S_{\text{sugar}}}{K_{s}+\sum S_{\text{sugar}}} \end{array}$$ (9)

where $k_{r}^{l}$ is a reactivation rate coefficient associated with each cell and species, where acid refers to acetate and butyrate, and sugar represents glucose and fructose, respectively. $K_{a}$ represents acid relief scale, and $K_{s}$ stands for sugar sensitivity parameter in *mM*. The kinetic parameters used in this study were determined by fitting the experimental data and are summarized in Table 1.

### Metabolite concentration profiles

Building upon the cell population dynamics estimated, the next step is to identify the governing reaction kinetics for the extracellular metabolites. Since the specific metabolic rates are highly nonlinear and depend on the unobservable physiological states derived in Eqs 1–9, we employed the Sparse Identification of Nonlinear Dynamics (SINDy) framework to discover the underlying model structure.

The mass balance for extracellular metabolite *j* in the perfusion bioreactor is governed by:


$$\begin{array}{c} \frac{dS_{j}}{dt}=D_{e}(S_{j,in}-S_{j})+R_{j}(𝐱) \end{array}$$ (10)

where $S_{j}$ denotes the concentration of metabolite *j*, and $D_{e}$ is the effective dilution rate accounting for the bleed fraction. The term $D_{e}(S_{j,in}-S_{j})$ represents the net convective transport due to medium inflow (feed) and outflow (bleed) under perfusion operation. $R_{j}(𝐱)$ represents the net biological reaction rate, but the physics governing $R_{j}$ is unknown. Therefore, we adopt a hybrid modeling architecture [58] that embeds a data-driven component into the underlying first-principles model, $D_{e}(S_{j,in}-S_{j})$.

This setting corresponds to a parallel-type hybrid model, in which the known physics term and the data-driven component jointly contribute to the overall dynamics [59]. Among various possible data-driven approaches, we employ basis-function-based methods to maintain interpretability. In this framework, the basis functions, referred to as a dictionary, consist of physically meaningful candidate terms, inspired by the interspecies reaction network shown in Fig 2.

***Remark 1.*** *The physical transport terms are analytically determined by the operating conditions. For each metabolite in the system in Eq 10,* $S_{j,in}$ *can be zero or non-zero, according to the feed composition. Before applying SINDy, the time-derivatives (*$dS_{j}/dt$*) were computed using central finite differences after smoothing the concentration profiles with a Savitzky-Golay filter (window size 11, polynomial order 2) to mitigate experimental noise.*

#### Using SINDy: Physiologically informed feature library.

Standard SINDy implementations typically rely on generic polynomial libraries, which often fail to capture saturation kinetics and metabolic switching inherent in bioprocesses. To address this, we constructed a physiologically informed candidate library Θ(𝐱) in MATLAB, and then applied structural constraints to enforce biological plausibility. The feature library was explicitly populated with the following functional forms derived, integrating the hidden physiological states estimated, which include biomass-coupled growth terms (active state), non-growth associated metabolism (dormant state), metabolic shifts, environmental and maintenance factors, constants, and concentrations of each metabolite. All candidates are listed in Table 2.

**Table 2:** Candidate function list from the library, Θ(x).

Candidates	Functional forms
**Biomass-coupled growth terms (active state)**	
Active growth of $C_{ac}$	$\mu_{C_{ac}}X_{C_{ac}}$
Active growth of $C_{lj}$	$\mu_{C_{lj}}X_{C_{lj}}$
**Non-growth associated metabolism (dormant state)**	
Dormant $C_{ac}$	$X_{C_{ac}^{*}}$
Dormant $C_{lj}$	$X_{C_{lj}^{*}}$
**Environmental and maintenance factors**	
H^+^ concentration from pH ([H^+^], in μM)	10^–*pH* + 6^
Active biomasses	$X_{C_{ac}}$, $X_{C_{lj}}$
**Metabolite concentrations**	
Constant	1
Sugar contents	$S_{glu},S_{fru}$
Metabolite concentrations	$S_{ace},S_{acn},S_{EtOH},S_{IPA},S_{BuOH},S_{buty}$
**Metabolic shifts**	
Acetate consumption (a)	$S_{ace}X_{C_{ac}}\frac{1}{1+P_{mix,C_{ac}}}$
	$S_{ace}X_{C_{ac}^{*}}\frac{1}{1+P_{mix,C_{ac}}}$
Acetate consumption (b)	$S_{ace}X_{C_{ac}}\frac{P_{mix,C_{ac}}}{1+P_{mix,C_{ac}}}$
	$S_{ace}X_{C_{ac}^{*}}\frac{P_{mix,C_{ac}}}{1+P_{mix,C_{ac}}}$
Butyrate consumption (a)	$S_{buty}X_{C_{ac}}\frac{1}{1+P_{mix,C_{ac}}}$
	$S_{buty}X_{C_{ac}^{*}}\frac{1}{1+P_{mix,C_{ac}}}$
Butyrate consumption (b)	$S_{buty}X_{C_{ac}}\frac{P_{mix,C_{ac}}}{1+P_{mix,C_{ac}}}$
	$S_{buty}X_{C_{ac}^{*}}\frac{P_{mix,C_{ac}}}{1+P_{mix,C_{ac}}}$
Acetone consumption	$S_{acn}X_{C_{lj}}\frac{1}{1+P_{mix,C_{lj}}}$
	$S_{acn}X_{C_{lj}^{*}}\frac{1}{1+P_{mix,C_{lj}}}$

(a): Uptake inhibited by total metabolic stress ($P_{mix}$). (b): Solventogenesis induction for survival under elevated stress.

#### Structural constraints and robust identification.

To ensure biological plausibility, we imposed structural masking on the regression. The feature space for each metabolite equation was restricted to its direct metabolic precursors and relevant regulatory factors. The unknown function $R_{j}(𝐱)$ was approximated as a sparse linear combination of candidate functions from a library Θ(𝐱). The feature library included linear and nonlinear terms derived from the measured state variables (metabolites) and the estimated population states (active/dormant biomass of $C_{ac}$ and $C_{lj}$). For instance, $S_{IPA}$ was constrained to depend on $S_{acn}$ and $X_{C_{lj}}$, reflecting the specific reduction pathway.

After the first-principles transport contribution was specified, the unknown biological reaction term was isolated from the metabolite mass balance (See Eq 10). For each metabolite *j*, the regression target was defined as:


$$\begin{array}{c} R_{j}=\frac{dS_{j}}{dt}-D_{e}(S_{j,in}-S_{j}) \end{array}$$ (11)

such that $R_{j}$ represents the net biological contribution to the extracellular metabolite dynamics. This term was then approximated as a sparse linear combination of physiologically informed candidate functions,


$$\begin{array}{c} R_{j}\approx \Theta_{j}\xi_{j} \end{array}$$ (12)

where $\Theta_{j}$ is the candidate library for metabolite *j* and $\xi_{j}$ is the corresponding coefficient vector. The coefficient vector was estimated using the STLS-Ridge procedure. Specifically, a ridge-regularized least-squares problem was solved iteratively as:


$$\begin{array}{c} \hat{\xi_{j}}=\text{arg}\min_{\xi_{j}}{\left|R_{j}-\Theta_{j}\xi_{j}\right|}_{2}^{2}+\gamma {\left|\xi_{j}\right|}_{2}^{2}. \end{array}$$ (13)

After each regression step, candidate terms with coefficient magnitudes below the threshold λ were removed, using the remaining active terms until the selected active set no longer changed. The nonzero coefficients obtained from this procedure define the identified data-driven reaction terms, as shown later in Eqs 14–19.

Sequential Threshold Least Squares (STLS) is an algorithm to solve the $\ell^{0}$ sparse least-squares problem, which relies on splitting to efficiently solve the least-squares portion while handling the sparse term via proximal methods [38]. However, metabolic time-series data inherently exhibit a high degree of collinearity, particularly between biomass growth and product formation, which renders standard least squares regression numerically unstable. To address this challenge, we employed a Ridge-regularized variant (STLS-Ridge).

This algorithm iteratively does the followings: (i) ridge regression with regularization parameter γ>0 to find a coefficient vector ξ; (ii) identify the indices for which the magnitudes of the corresponding components of the coefficient vector are less than the given threshold λ>0; and (iii) remove dictionary candidates corresponding to the indices selected above. In this work, γ and λ are set to 10^–6^ and 0.5, respectively. To ensure scale invariance during the regression, the columns of the feature library Θ were normalized to unit Euclidean length prior to fitting. The identification process utilized the full dataset without splitting, maximizing the information available for capturing the complex dynamics of the coculture system.

## Results and discussion

### Estimating the concentration profile of biomasses

rRNA-FISH detects ribosome-rich, translationally active cells rather than the entire community [47,60], which complicates direct inference of population structure and activity. Consequently, a central challenge in modeling the $C_{ac}$-$C_{lj}$ coculture is the limited observability of underlying cell physiological states. While the total biomass concentration and active cell concentrations can be experimentally obtained through the OD and fluorescence measurements, the distribution of dormant (non-reproducing but metabolically active) subpopulations remains inaccessible. However, resolving these hidden physiological states is a prerequisite for interpreting the complex metabolic dynamics of the system.

The developed kinetic model (Eqs 1–9) addresses this observability gap by mathematically resolving the aggregate data from Fig 3 into species-specific trajectories. According to Fig 4a, the model accurately tracks the active $C_{ac}$ and $C_{lj}$ populations. The reconstructed trajectories show that the active biomass observed in Fig 3 is not dominated by a single species throughout the fermentation. Instead, the system exhibits a sequential population structure in which $C_{ac}$ initially dominates the active biomass, followed by a later expansion of $C_{lj}$.

**Fig 3:**
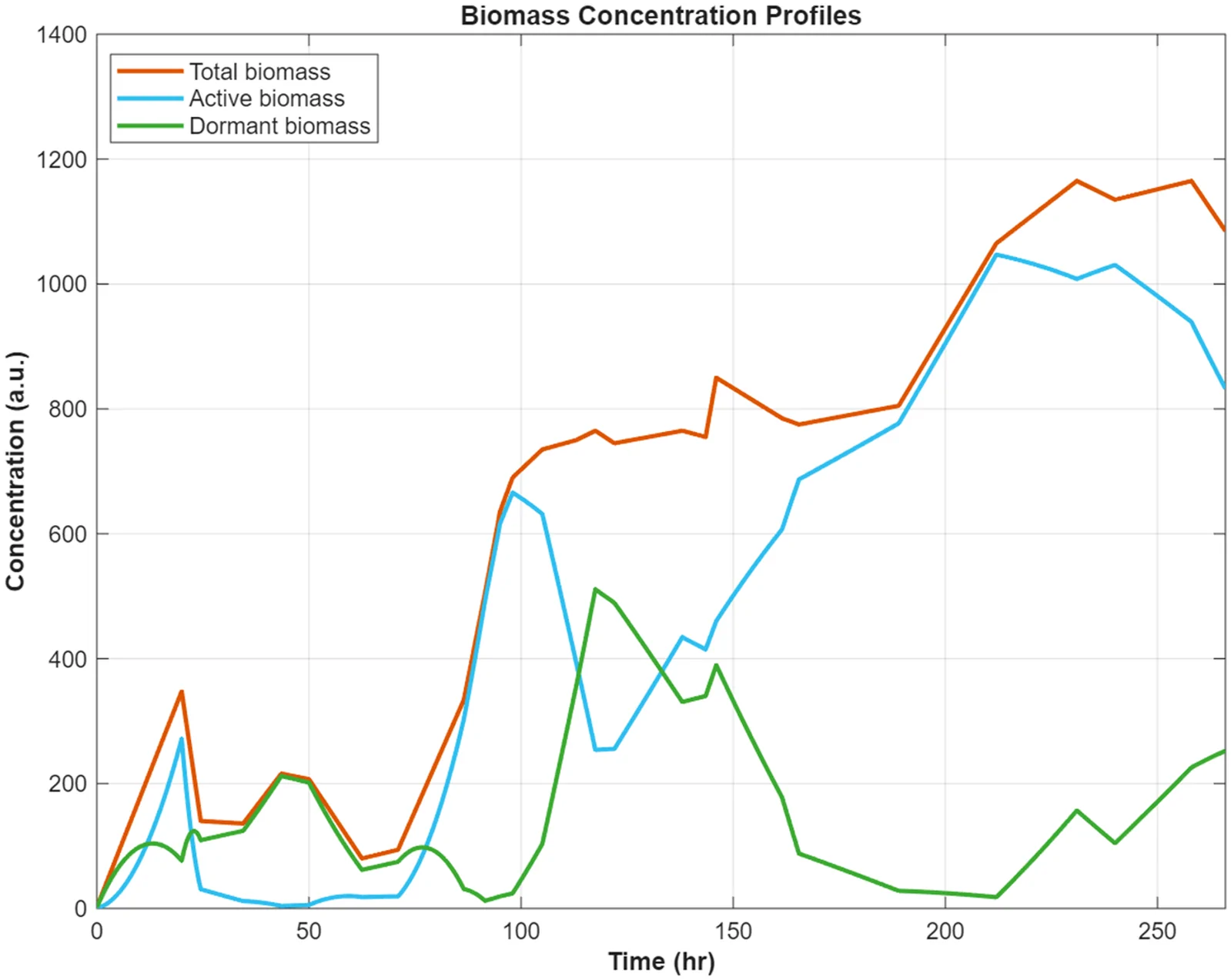
Experimentally constrained population information used for latent-state reconstruction. Total biomass was estimated from OD_600_, whereas the active cell population was obtained from rRNA-FISH/flow cytometry measurements. Dormant biomass was not directly measured, but was inferred as a latent state in the structured population model.

**Fig 4:**
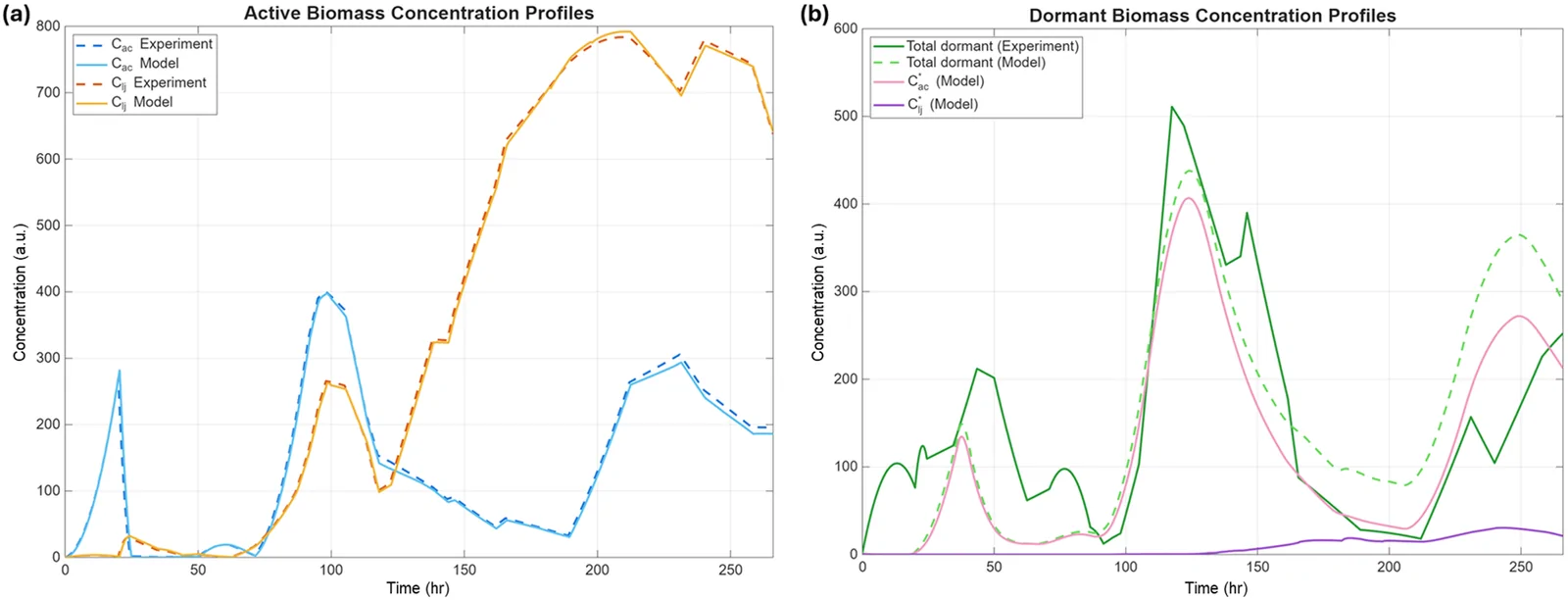
Comparison between experimentally constrained active population data and model-reconstructed population states. A: Active biomass concentrations. B: Model-reconstructed dormant biomass concentrations.

Here, the dormant biomass trajectories should be interpreted as model-reconstructed latent states. Experimentally, OD_600_ provided total biomass information, while rRNA-FISH/flow cytometry measurements provided active cell population information; the dormant fractions were inferred through the structured population model rather than measured directly. Accordingly, trajectory-level performance metrics were reported only for experimentally measured metabolites and experimentally constrained active-biomass trajectories. A notable feature is evident in the reconstructed dormant populations shown in Fig 4b. The model takes the aggregate ‘total dormant biomass’ and resolves it into $C_{ac}^{*}$ and $C_{lj}^{*}$. At the early stage, there is a significant spike in dormant $C_{ac}$ cells. This behavior is consistent with elevated organic acid stress, including butyrate supplied in the feed stream. Under such conditions, the influx of undissociated acids can exceed the rate at which these compounds are metabolized or detoxified by the cells. Therefore, undissociated acids accumulate and disrupt the proton motive force, inducing a stress-driven transition to dormancy and leading to the ‘death valley’ observed in Fig 4a. In this regime, the expected metabolic role of $C_{ac}$ as a butyrate reducer to butanol is temporarily suppressed as the cells become inhibited.

The subsequent recovery of $C_{ac}$ from 66 h onwards reflects a metabolic state transition rather than passive survival. According to the reactivation kinetics (Eq 9), the reactivation of dormant cells is associated with progressive alleviation of acid stress and the restoration of metabolic homeostasis. As accumulated acids are gradually reassimilated, a fraction of $C_{ac}^{*}$ cells regain metabolic activity and resume solventogenesis. Once reactivation begins, the growing active biomass promotes acid reassimilation and IPA formation, reinforcing the transition from an acid-inhibited state to a solventogenic active phase.

Another important observation in Fig 4a is the delayed growth of $C_{lj}$, which becomes dominant only after the recovery of $C_{ac}$ (>120 h). This sequential succession is consistent with the structured population model, in which both growth (Eqs 3–4) and state transitions (Eqs 1–2) depend on the composite inhibition burden (Eq 6). During the early phase (<70 h), the growth of $C_{lj}$ remains limited despite its presence in the reactor. This delayed expansion likely arises from a combination of environmental stress and limited availability of substrates supporting autotrophic metabolism. Although organic acid concentrations had not yet reached their peak levels, the metabolic environment remained unfavorable due to transient acid accumulation and associated physiological stress. In addition, autotrophic growth of $C_{lj}$ depends on the availability of gaseous substrates (H_2_/CO_2_) through the Wood–Ljungdahl pathway (Eq 4), which can further constrain growth in gas-fermentation environments. It is also well known that $C_{lj}$ typically does not reach very high cell densities in conventional CO_2_/H_2_ fermentation systems [61], where gas availability and mass-transfer limitations frequently restrict growth [62]. In contrast, the substantial $C_{lj}$ expansion observed in the later stage of this coculture suggests that the metabolic activity of $C_{ac}$ gradually stabilizes the environment and enables more favorable conditions for $C_{lj}$ growth.

As $C_{ac}$ progressively reassimilates acids and the system transitions toward a more permissive metabolic regime, $C_{lj}$ resumes active growth through its mixotrophic capability on fructose and gases (Eq 4), enabling its later-phase expansion. Therefore, the late $C_{lj}$ surge is interpreted as an emergent outcome of environmental stabilization and regime transition in the coculture, rather than a solvent-induced activation signal. The model therefore suggests a sequential population structure rather than direct competition between the two species. In this interpretation, $C_{ac}$ appears to play an early conditioning role that gradually stabilizes the metabolic environment, facilitating the transition toward a solventogenic regime. While the metabolite profiles do not indicate extremely high levels of toxic byproducts in the early stage, the transient accumulation of organic acids and associated physiological stress may still suppress cell activity and induce dormancy, as reflected in the model structure through the inhibition and state-transition terms. As the culture progresses, the metabolic turnover driven by $C_{ac}$ reduces these stresses and establishes conditions that are more favorable for the subsequent expansion of $C_{lj}$.

### Identification of metabolic dynamics via SINDy

Building upon the reconstructed population dynamics discussion, the next step is to interpret how these physiological states affect extracellular metabolite dynamics. Using the reconstructed active and dormant biomass trajectories, the SINDy framework identifies kinetic structures for the key metabolites in this coculture system. The sparse regression strategy extracts sparse, model-supported associations from the data. The resulting equations therefore provide an interpretation of how organic acid turnover, solvent formation, and interspecies metabolite exchange emerge from the underlying physiological dynamics.

Fig 5 compares the experimental measurements with the SINDy-predicted trajectories for substrates and products. The agreement supports the suitability of the physiologically informed library Θ(𝐱). The agreement across all state variables suggests that the identified kinetic structures capture the dominant regulatory interactions governing metabolite dynamics under the operating conditions. To further evaluate whether the full physiologically informed library was justified relative to simpler alternatives, we performed a reduced-model comparison using different libraries with progressively increasing biological information. The trajectory-level comparison and complexity-penalized analysis are provided in the SI. In addition, various model robustness analyses were conducted and summarized in the SI to evaluate the stability of the identified SINDy structure.

**Fig 5:**
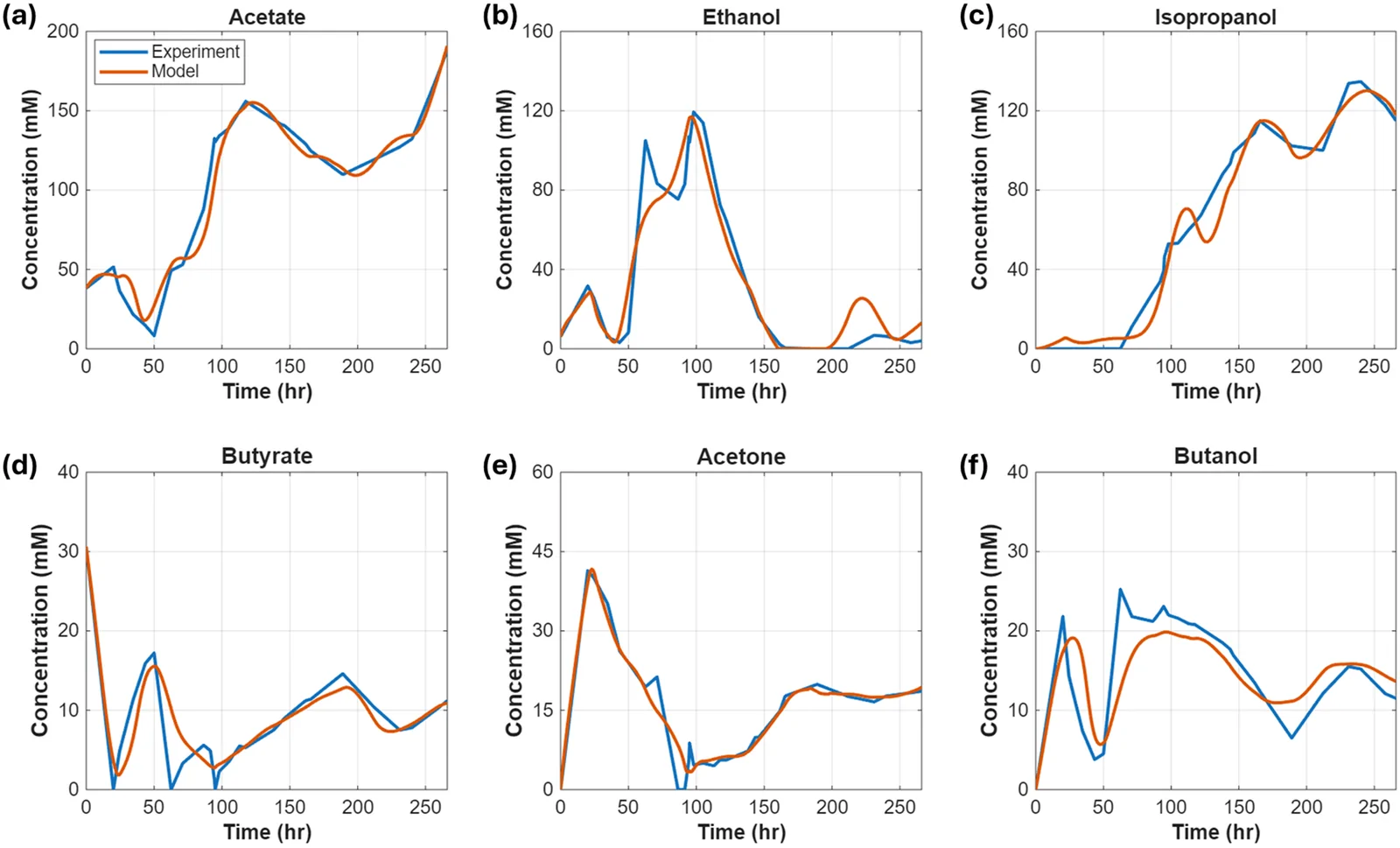
A comparison plot between the experimental data and SINDy results for each metabolite.

To interpret how the reconstructed physiological states regulate extracellular metabolites, the identified kinetic structures are analyzed for key metabolites in the coculture system. We first examine the dynamics of organic acids, which play a central role in metabolic regulation and solventogenic transitions. Subsequently, we discuss solvent formation pathways and their coupling to physiological state shifts and interspecies metabolic interactions.

#### Organic acids.

Organic acids play a central regulatory role in clostridial fermentations since their accumulation simultaneously imposes metabolic stress and triggers the transition toward solventogenesis. In this coculture system, acetate and butyrate therefore function not only as metabolic intermediates but also as key regulators of the physiological state of the population. The SINDy framework identified the kinetic structures for both acids, suggesting how their turnover is coupled to the metabolic activity of $C_{ac}$ and to the environmental conditions in the fermentation broth.

The identified kinetic structure for acetate dynamics is given as follows:


$$\begin{array}{cc} \frac{dS_{ace}}{dt}=\underset{\text{First-principles}}{\underbrace{-0.075S_{ace}}} & +\underset{\text{Data-driven components}}{\underbrace{3.898+0.4523[H^{+}]-0.0986\mu_{C_{ac}}X_{C_{ac}}}}\\ & \underset{\text{Data-driven components}}{\underbrace{-0.2075X_{C_{ac}}-0.0702X_{C_{ac}^{*}}}} \end{array}$$ (14)

Note that the first metabolite-proportional term applies for all species, indicating the effective dilution effect under perfusion operation. The coefficient $0.075\;h^{-1}$ in the first-principles transport term represents the effective dilution rate under perfusion operation. Based on the feed flow rate $F\approx 0.30\;L\;h^{-1}$ and the total working volume V=3.6 L, the nominal dilution rate is $D=F/V\approx 0.083\;h^{-1}$. After accounting for the 10% bleed fraction, the effective dilution rate for extracellular metabolites becomes $D_{e}=0.9D\approx 0.075\;h^{-1}$. Thus, when a metabolite is absent from the feed stream, $D_{e}(S_{j,in}-S_{j})$ simplifies to $-0.075S_{j}$.

Eq 14 indicates that acetate turnover is represented mainly through $C_{ac}$ terms. The major contribution to acetate consumption is associated with active metabolism of $C_{ac}$, as represented by the growth-associated term $\mu_{C_{ac}}X_{C_{ac}}$ and the biomass-proportional term $X_{C_{ac}}$. The presence of the dormant biomass term $X_{C_{ac}^{*}}$ is also noticeable, which implies that acetate metabolism takes place even when cells are not actively growing. This behavior is consistent with the solventogenic phase of $C_{ac}$ metabolism, where accumulated acids are reassimilated and redirected toward solvent production. This may reflect maintenance metabolism and redox-balancing reactions that persist through enzymatic activities.

In addition, the kinetic structure for butyrate dynamics is identified as follows:


$$\begin{array}{cc} \frac{dS_{buty}}{dt}= & \;\underset{\text{First-principles}}{\underbrace{0.075(S_{buty,in}-S_{buty})}}\\ & -\underset{\text{Data-driven components}}{\underbrace{1.127+0.0228[H^{+}]-0.196\mu_{C_{ac}}X_{C_{ac}}-0.0425S_{BuOH}}} \end{array}$$ (15)

where $S_{buty,in}=30$. For butyrate, the primary biological contribution comes from the growth-associated term, $\mu_{C_{ac}}X_{C_{ac}}$, indicating that butyrate consumption is closely linked to the active growth of $C_{ac}$. In contrast, no biomass-only or dormant-cell terms were retained. This suggests that butyrate turnover is sensitive to active metabolic flux rather than maintenance-level metabolism, as the reduction of butyrate to butanol requires reducing equivalents (*e.g.*, NAD(P)H) that are primarily generated through glycolytic activity. Additional effects are captured through environmental variables. More specifically, the dependence on [*H*^+^] reflects the influence of pH-dependent acid-base equilibrium on measurable butyrate concentration. Meanwhile, the negative coupling with $S_{BuOH}$ suggests a feedback relationship between solvent accumulation and butyrate metabolism. As butanol accumulates in the broth, the associated metabolic stress may suppress the pathways responsible for butyrate turnover.

These results indicate a mechanistic distinction between the two organic acids. While acetate dynamics involve both growth-associated and maintenance-level metabolic activities, butyrate turnover appears to be more directly linked to active solventogenic metabolism of the $C_{ac}$ population.

#### Solvent production dynamics.

Ethanol production follows a growth-associated pattern, as shown in Eq 16, suggesting that it is a primary fermentation product of $C_{ac}$.


$$\begin{array}{cc} \frac{dS_{EtOH}}{dt}=\underset{\text{First-principles}}{\underbrace{-0.075S_{EtOH}}} & +\underset{\text{Data-driven components}}{\underbrace{16.71+0.241\mu_{C_{ac}}X_{C_{ac}}-0.0376X_{C_{ac}}-0.0557X_{C_{ac}^{*}}}}\\ & \underset{\text{Data-driven components}}{\underbrace{-0.0262S_{glu}-0.108S_{ace}-0.219S_{acn}}} \end{array}$$ (16)

This suggests that the primary biological driver is the growth-associated term $\mu_{C_{ac}}X_{C_{ac}}$, which is consistent with the well-established metabolic role of ethanol as a primary fermentation product generated during solventogenesis. In addition to the growth-coupled pathway, the model retains biomass-proportional terms for both active and dormant populations. This suggests that ethanol metabolism may also involve non-growth-associated metabolic activity similar to the behavior observed for acetate. This behavior may reflect the redistribution of reducing equivalents during metabolic shifts. While $C_{lj}$ is capable of producing small amounts of ethanol under mixotrophic conditions, its contribution is likely limited relative to the primary ethanol production associated with $C_{ac}$ metabolism. Furthermore, the negative coupling with glucose, acetate, and acetone indicates that ethanol production is modulated by the surrounding metabolic environment. As these metabolites accumulate, the associated metabolic feedback may redistribute intracellular fluxes toward alternative solvent pathways, reducing the relative flux directed to ethanol formation.

The SINDy-identified kinetic structure for butanol is comparatively compact, as highlighted below:


$$\begin{array}{cc} \frac{dS_{BuOH}}{dt}= & \underset{\text{First-principles}}{\underbrace{-0.075S_{BuOH}}}+\underset{\text{Data-driven components}}{\underbrace{1.749-0.0227S_{buty}-0.0502[H^{+}]}} \end{array}$$ (17)

Eq 17 suggests that extracellular butanol dynamics are captured mainly by environmental and metabolite concentrations rather than direct biomass-related terms. In particular, the negative coupling with $S_{buty}$ indicates that butanol accumulation increases as butyrate is consumed, reflecting the metabolic transition from acidogenesis to solventogenesis in clostridial fermentation. The dependence on [*H*^+^] further suggests that butanol accumulation is sensitive to acid stress. High proton concentration corresponds to stronger weak-acid stress, which imposes an energetic burden on cellular homeostasis. Under such conditions, metabolic resources may be redirected away from solvent production, resulting in reduced net butanol accumulation in the broth.

Although no explicit biomass-dependent terms were retained, this does not imply that butanol formation is independent of cellular metabolism. Instead, the identified structure indicates that the dominant drivers of butanol dynamics in this dataset are captured through environmental variables that implicitly reflect the metabolic state of the culture.

It is noticeable that acetone and isopropanol represent a coupled solvent pair in the coculture system, reflecting the metabolic interaction between $C_{ac}$ and $C_{lj}$. In clostridial solventogenesis, acetone is produced by $C_{ac}$ through the decarboxylation of acetoacetate [63], whereas isopropanol can be formed through the subsequent reduction of acetone by $C_{lj}$ [46]. The SINDy framework captures this interaction by identifying distinct yet interconnected kinetic structures for acetone and isopropanol dynamics.

The kinetic structure for acetone dynamics is given by Eq 18, highlighting that its concentration is shaped by both production from $C_{ac}$ metabolism and consumption associated with the $C_{lj}$ population.


$$\begin{array}{cc} \frac{dS_{acn}}{dt}=\underset{\text{First-principles}}{\underbrace{-0.075S_{acn}}} & +\underset{\text{Data-driven components}}{\underbrace{0.892+0.0250[H^{+}]-0.128\mu_{C_{ac}}X_{C_{ac}}-0.0308\mu_{C_{lj}}X_{C_{lj}}}}\\ & \underset{\text{Data-driven components}}{\underbrace{+0.0103X_{C_{ac}}-0.226X_{C_{lj}^{*}}-0.0404X_{C_{lj}^{*}}S_{acn}\frac{1}{1+P_{mix,C_{lj}}}}} \end{array}$$ (18)

This equation indicates that acetone formation is closely associated with the metabolic activity of $C_{ac}$ during solventogenesis. Particularly, the presence of $C_{ac}$-related terms suggests that acetone production is consistent with the solventogenic metabolism of the $C_{ac}$ population. This behavior is consistent with the classical ABE fermentation pathway, where acetone is produced as a characteristic solvent during the acid reassimilation phase. However, acetone does not accumulate indefinitely in the coculture system. The presence of $C_{lj}$-dependent terms suggests that acetone is rapidly removed through downstream metabolic reactions mediated by $C_{lj}$. In particular, the terms involving $C_{lj}$ biomass imply that acetone turnover is strongly influenced by the presence and physiological state of the $C_{lj}$ population. This observation is consistent with the fact that acetone reduction to isopropanol is mediated by $C_{lj}$, while $C_{ac}$ does not possess the enzymatic machinery for this conversion.

The identified kinetics for isopropanol dynamics are described by Eq 19.


$$\begin{array}{cc} \frac{dS_{IPA}}{dt}=\underset{\text{First-principles}}{\underbrace{-0.075S_{IPA}}} & +\underset{\text{Data-driven components}}{\underbrace{0.0192S_{acn}+0.0196\mu_{C_{lj}}X_{C_{lj}}+0.0291X_{C_{lj}}}}\\ & \underset{\text{Data-driven components}}{\underbrace{+0.171X_{C_{lj}^{*}}-0.0396X_{C_{lj}^{*}}S_{acn}\frac{1}{1+P_{mix,C_{lj}}}}} \end{array}$$ (19)

The structure of the isopropanol equation shows a clear retained acetone contribution, $S_{acn}$, indicating that acetone acts as a metabolic precursor for isopropanol formation. This relationship suggests that isopropanol production is not an independent pathway but rather arises from the reduction of acetone within the coculture system. Unlike acetone, the isopropanol production is associated with the $C_{lj}$ population. The positive coefficient for $\mu_{C_{lj}}X_{C_{lj}}$ shows that isopropanol formation increases as $C_{lj}$ actively proliferates, reflecting the reduction of acetone to isopropanol during $C_{lj}$ metabolism. Additionally, the presence of $X_{C_{lj}}$ and $X_{C_{lj}^{*}}$ suggests that the enzymatic machinery responsible for this conversion remains active throughout the fermentation process.

Additionally, both Eqs 18–19 include terms involving $C_{lj}^{*}$ modulated by the toxicity factor $P_{mix}$. These terms indicate that metabolic activity related to acetone turnover and isopropanol formation is sensitive to the overall stress level of the fermentation environment. It suggests that the conversion flux decreases as the toxicity burden increases, providing a model-supported interpretation for the slowdown of solvent production observed at high product concentrations. Overall, Eqs 18–19 are associated with a sequential metabolic interaction between the two species. The acetone-isopropanol coupling reflects a syntrophic metabolic relay in which solvent intermediates generated by one organism become substrates for downstream transformation by the other one.

***Remark 2.*** *Throughout this hybrid modeling approach, the SINDy-identified equations in Eqs 14–19 follow the generic metabolite mass balance structure defined in Eq 10.* *This consistency ensures that the identified dynamics remain physically interpretable, where each metabolite rate is expressed as the combination of dilution and net metabolic conversion terms. Consequently, the sparse regression procedure identifies the governing kinetic contributions while preserving the mechanistic* *mass-balance formulation of the system.*

The reduced-model comparison in the SI further supports the use of the full physiologically informed library at the overall model level. In this comparison, adding active-biomass terms substantially improved trajectory reconstruction relative to metabolite/pH-only models, and the full active/dormant/stress-informed library provided the best overall balance between trajectory fit and model complexity.

In conclusion, the SINDy-identified kinetic structures suggest a coordinated metabolic organization within the $C_{ac}$-$C_{lj}$ coculture system. Rather than simple growth competition between the two organisms, the identified equations indicate a structured metabolic organization organized through physiological state transitions and metabolite-mediated interactions. Most notably, the discovered equations highlight the central role of acetone as an intermediate metabolite linking the metabolic activities of the two species. The identified kinetic structures suggest a syntrophic metabolic relay in which acetone produced by $C_{ac}$ is subsequently reduced to isopropanol by $C_{lj}$, providing a model-supported interpretation for the experimentally observed coupling between acetone turnover and isopropanol formation. In addition, organic acid dynamics constitute the primary kinetic layer, where acetate and butyrate accumulation contribute to metabolic stress and trigger transitions between active and dormant states. The identified solvent production pathways further suggest that solventogenesis emerges as a downstream metabolic regime associated with the accumulation of organic acids, consistent with the known shift from acidogenic to solventogenic regimes in clostridial fermentations. The results therefore suggest that optimal solvent production in this coculture system emerges not from maximizing individual species growth, but from maintaining a balanced physiological state distribution and supporting coupled metabolite conversion pathways between the two organisms.

The SINDy-identified structures provide a data-constrained kinetic interpretation of the observed coculture dynamics under perfusion operation. In particular, the temporal ordering of organic acid accumulation, active-dormant population shifts, acetone turnover, and isopropanol formation is consistent with a sequential interpretation of stress response and interspecies metabolic exchange. Future perturbation studies that vary organic acid loading, gas availability, or initial species composition would provide a direct experimental route to further test these inferred interactions and extend the present model-based interpretation. In addition, the reduced-library and AIC/BIC analyses support the practical usefulness of dormant- and stress-related terms at the overall model level, while independent perturbation experiments and additional state-specific measurements would further strengthen the evaluation of identifiability and generalizability. Future extensions may also connect the present dynamic coculture framework with transfer-learning or molecular-property modeling approaches for extrapolation across new substrates, feed conditions, or chemical environments [64,65]. Reactor-scale extensions could further incorporate compartment or hydrodynamic modeling strategies to account for spatial heterogeneity, mixing, and reactor-design effects during scale-up [66,67].

## Conclusion

This work demonstrates that integrating mechanistic kinetic modeling with data-driven sparse identification (SINDy) provides a hybrid modeling framework for resolving the observability limits inherent in mixed-culture fermentations. By mathematically resolving the aggregate biomass into active and dormant states, we investigated diverse mechanistic insights, including that the complex dynamics of cocultures are driven by a sophisticated interplay of stress-induced dormancy and sequential metabolic handoffs, rather than simple growth competition.

Beyond the specific case of $C_{ac}$ and $C_{lj}$, this study highlights the broader potential of such a modeling strategy to infer hidden population-state and metabolite-dynamic relationships in biological systems without extensive proteomic or transcriptomic datasets. The identification of model-supported syntrophic interactions and distinct survival strategies can provide mechanistic guidance for the optimization of coculture fermentation processes. In particular, the identification of the acetone–isopropanol metabolic relay further supports how cooperative metabolic interactions can be quantitatively resolved using the proposed framework. Ultimately, this methodology offers a potential basis for the rational design and control of robust microbial consortia for sustainable bioproduction.

## Supporting information

S1 Text Supporting information for model robustness analyses and additional operating-regime assessment. This file contains additional analyses supporting the model robustness evaluation and additional operating-regime assessment.
(PDF)
