# Supplementary material for: From observable fermentation data to hidden cell states: A modeling study of a mixotrophic Clostridium coculture under perfusion mode
Source: PLoS Comput Biol. 2026 Sep 28;22(9):e1014759. doi: 10.1371/journal.pcbi.1014759 (PMC13619069; doi:10.1371/journal.pcbi.1014759)
Supplement: Supplementary file 1 [file pcbi.1014759.s001.pdf]

Supporting Information for:

From observable fermentation data to hidden cell states: A modeling study of a mixotrophic Clostridium coculture under perfusion mode

1. Model robustness

To validate model robustness, additional analyses have been performed.

1.1. Trajectory-level performance metrics

For experimentally measured or experimentally constrained quantities, trajectory-level metrics are calculated to further quantify model performance. The evaluated variables include active C_{ac} and active C_{lj}, as well as the six extracellular metabolites identified by the final SINDy model. For each trajectory, MSE, RMSE, MAE, R^2, and relative AUC (rAUC) error were calculated. The rAUC error was defined as the absolute difference between the model-predicted and experimental trajectory areas, normalized by the experimental trajectory area. This metric was included since several metabolite profiles contain sharp transient regions, where pointwise metrics such as R^2 may appear lower even when the overall time-integrated trend is captured well. Therefore, R^2 and rAUC error were interpreted together.

Table A: Trajectory-level performance metrics for experimentally measured or experimentally constrained variables.

**Variable**	**MSE**	**RMSE**	**MAE**	$\mathbf{R^2}$	**rAUC error (%)**
Active C_{ac}	64.11	8.01	5.31	0.9950	2.81
Active C_{lj}	51.32	7.16	5.39	0.9995	0.09
Acetate	61.49	7.84	5.59	0.9726	0.15
Ethanol	78.30	8.85	5.57	0.9422	3.24
Isopropanol	56.24	7.50	5.70	0.9768	2.39
Butyrate	5.13	2.27	1.43	0.7816	0.07
Acetone	3.30	1.82	1.04	0.9610	0.07
Butanol	12.47	3.53	2.70	0.6063	0.62

As summarized in Table A, the model captured the major active biomass and extracellular metabolite trajectories. The active C_{ac} and C_{lj} trajectories showed high R^2 values of 0.9950 and 0.9995, respectively, with small relative AUC errors, indicating that the reconstructed active-population dynamics were consistent with the experimentally constrained population trends. For the extracellular metabolites profiles, acetate, ethanol, isopropanol, and acetone were reconstructed with high trajectory-level agreement, with R^2 values above 0.94. The butyrate and butanol trajectories showed lower pointwise R^2 values compared with the other metabolites.

However, their rAUC errors remained small, 0.07% for butyrate and 0.62% for butanol. This indicates that the model captured the overall time-integrated metabolite behavior, although local transient deviations reduced the pointwise R^2. This interpretation is consistent with the observed trajectory shapes, where metabolites with relatively low concentrations ranges or sharper local changes are more sensitive to small temporal mismatches in pointwise metrics.

Overall, all evaluated variables showed relative AUC errors below 3.3%, supporting that the final model reproduced the dominant time-dependent trends of the experimentally measured or constrained trajectories. These results highlight that the model reproduced the overall temporal behavior of the measured or experimentally constrained trajectories.

1.2. Noise-injection experiment

To evaluate the robustness of the identified SINDy model against measurement uncertainty, Gaussian noise was directly added to the extracellular metabolite measurements at 5% and 10% of each metabolite's dynamic range. For each noise level, 100 independent noisy datasets were generated, and the full identification workflow, including smoothing, derivative estimation, transport-term subtraction, candidate-library construction, and STLS-Ridge sparse identification, was repeated.

Figs. A–B visualize the resulting perturbations for the six extracellular metabolites used in the final model. The red curves indicate the original experimental trajectories, the dark gray lines show representative noisy realizations, the gray shaded regions indicate the 5th–95th percentile range across all 100 trials, and the green curves represent the median noisy trajectory.

As expected, the perturbation range increased from the 5% to the 10% noise case, but the noisy trajectories remained centered around the original measurements. The noise-trained models were then evaluated against the original clean trajectory dataset using trajectory-level performance and robustness metrics, including RMSE, R^2, and SINDy-term retention. The performance metrics are summerized in Table B. These results indicate that the identified model is robust to moderate measurement noises, with only gradual degradation in trajectory-level accuracy and minimal changes in the selected sparse structure.

Table B: Summary of noise-injection robustness metrics.

**Model**	**RMSE**	$\mathbf{R^2}$	**SINDy-term retention**
Full original model	5.3004	0.8734	1.0000
5% noise injection	6.0275 ± 0.0873	0.8605 ± 0.0029	0.9943
10% noise injection	7.0823 ± 0.1735	0.8318 ± 0.0073	0.9908

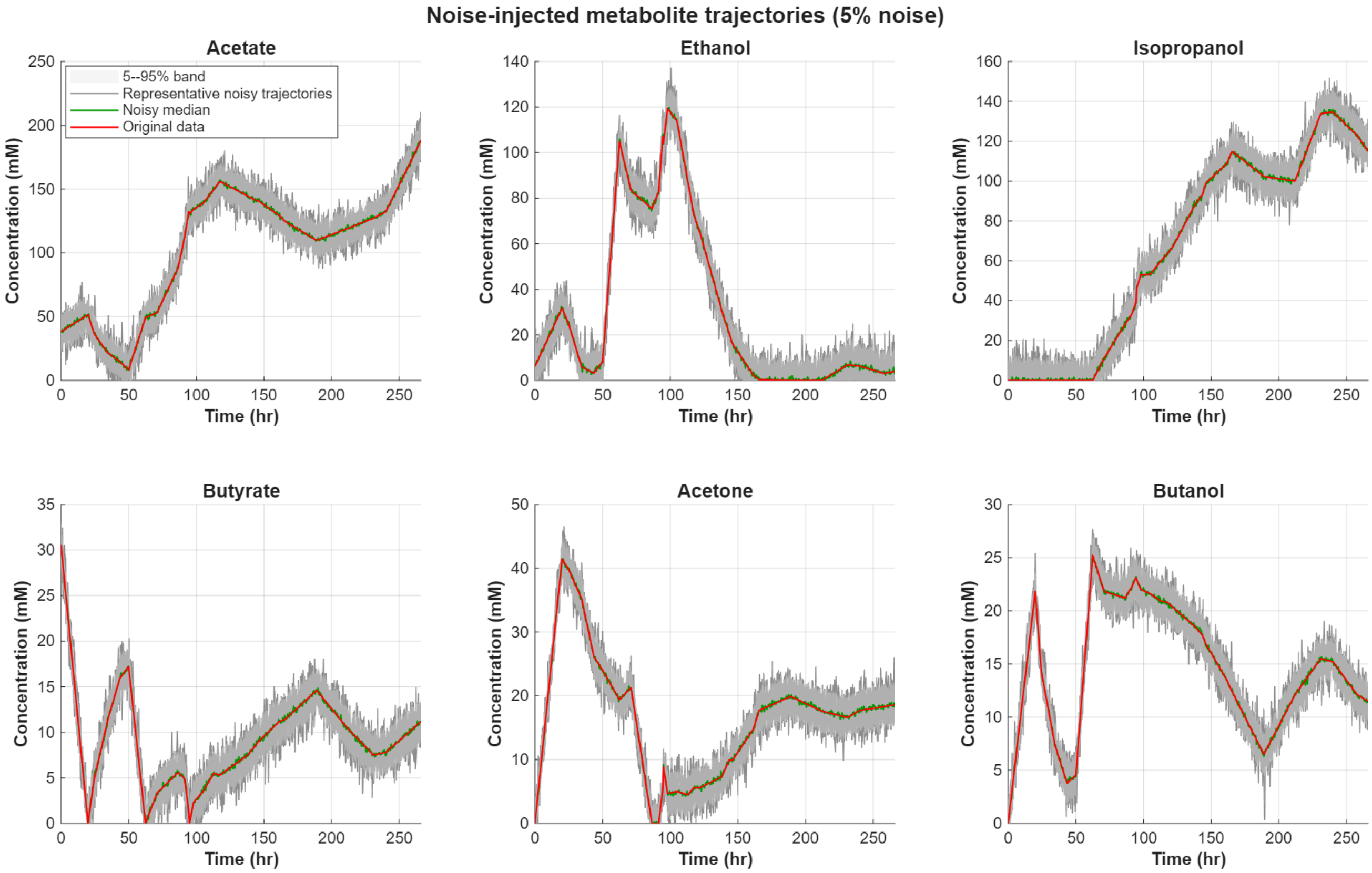

Figure A: Visualization of noise-injected metabolite trajectories under 5% Gaussian measurement noise. The blue curves denote the original experimental trajectories, gray lines show representative noisy realizations, gray shaded regions indicate the 5th–95th percentile range across 100 trials, and green curves represent the median noisy trajectory.

1.3. Missing-data subset robustness analysis

To evaluate whether the identified SINDy equations were overly dependent on a small subset of regression points, we performed a missing-data subset robustness analysis. Unlike the noise-injection experiment, the original measured metabolite values were not perturbed. Instead, the full dataset was first processed using the same smoothing, derivative estimation, transport-term subtraction, and candidate-library construction procedure used for the final model. Then, 10% and 20% of the resulting regression rows were randomly removed before STLS-Ridge identification. For each removal fraction, the model identification was repeated for 100 independent trials.

For each subset-trained model, the identified equations were evaluated on the original full trajectory dataset. Figs. C–D show the resulting trajectory-level predictions for the six extracellular metabolites. The red curves denote the original experimental trajectories, gray lines show representative subset-trained model predictions, and green curves represent the median prediction.

The robustness of the subset-trained models was quantified using trajectory-level performance

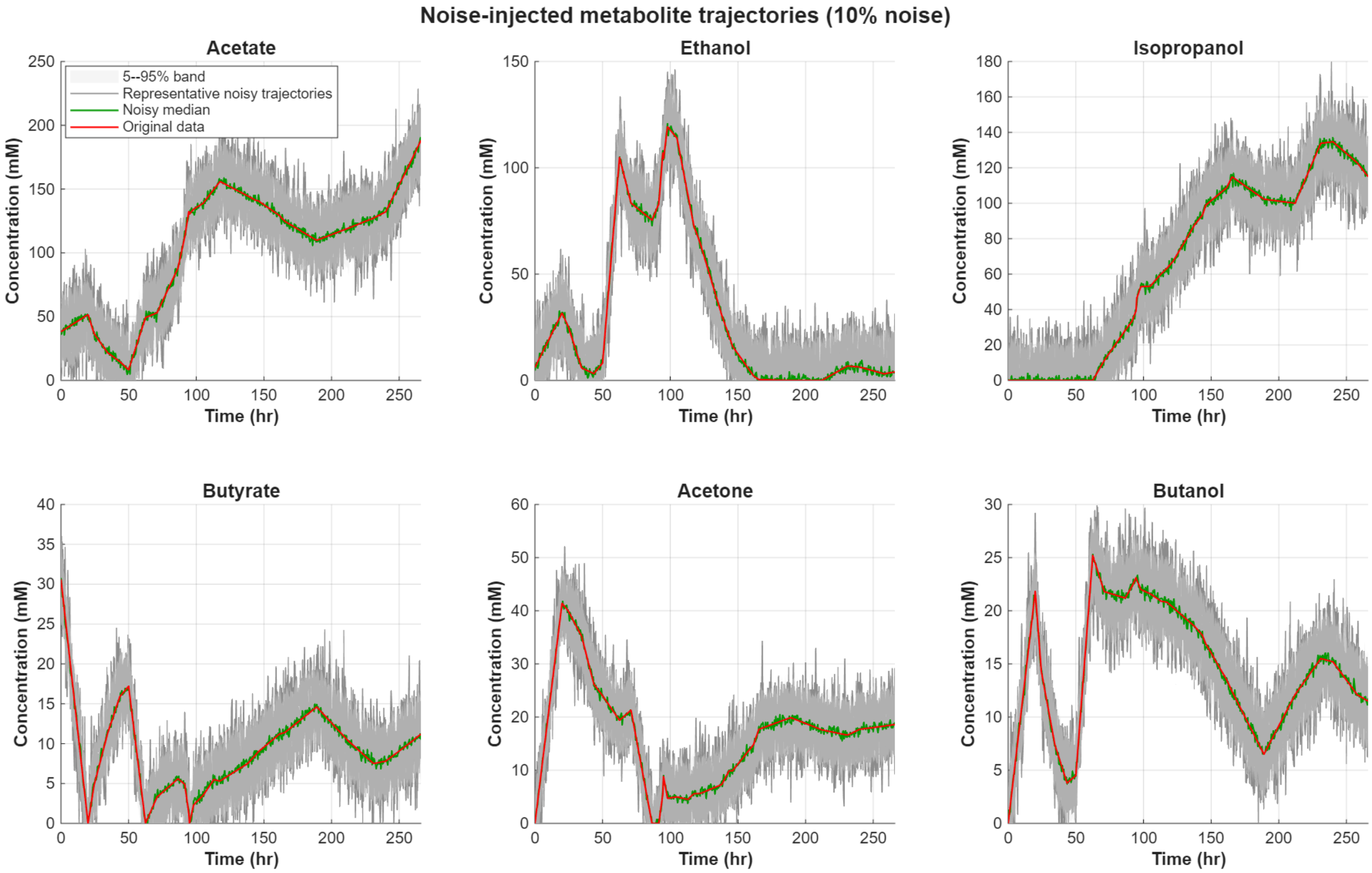

Figure B: Visualization of noise-injected metabolite trajectories under 10% Gaussian measurement noise. The blue curves denote the original experimental trajectories, gray lines show representative noisy realizations, gray shaded regions indicate the 5th–95th percentile range across 100 trials, and green curves represent the median noisy trajectory.

metrics, including RMSE, R^2, and SINDy-term retention. The detailed performance metrics are summarized in Table C. These results indicate that the identified equations were not driven by a small number of individual regression rows and remained robust to moderate missing-data perturbations.

Table C: Summary of missing-data subset robustness metrics.

**Model**	**RMSE**	$\mathbf{R^2}$	**SINDy-term retention**
Full original model	5.3004	0.8734	1.0000
10% row removal	5.3081 ± 0.0350	0.8735 ± 0.0025	0.9928
20% row removal	5.3278 ± 0.0534	0.8721 ± 0.0040	0.9834

1.4. Hyperparameter sensitivity analysis

To evaluate whether the identified SINDy structure was sensitive to the sparsity threshold, we performed a hyperparameter sensitivity analysis for the STLS-Ridge threshold parameter λ. The same preprocessing, smoothing, derivative estimation, transport-term subtraction, candidate

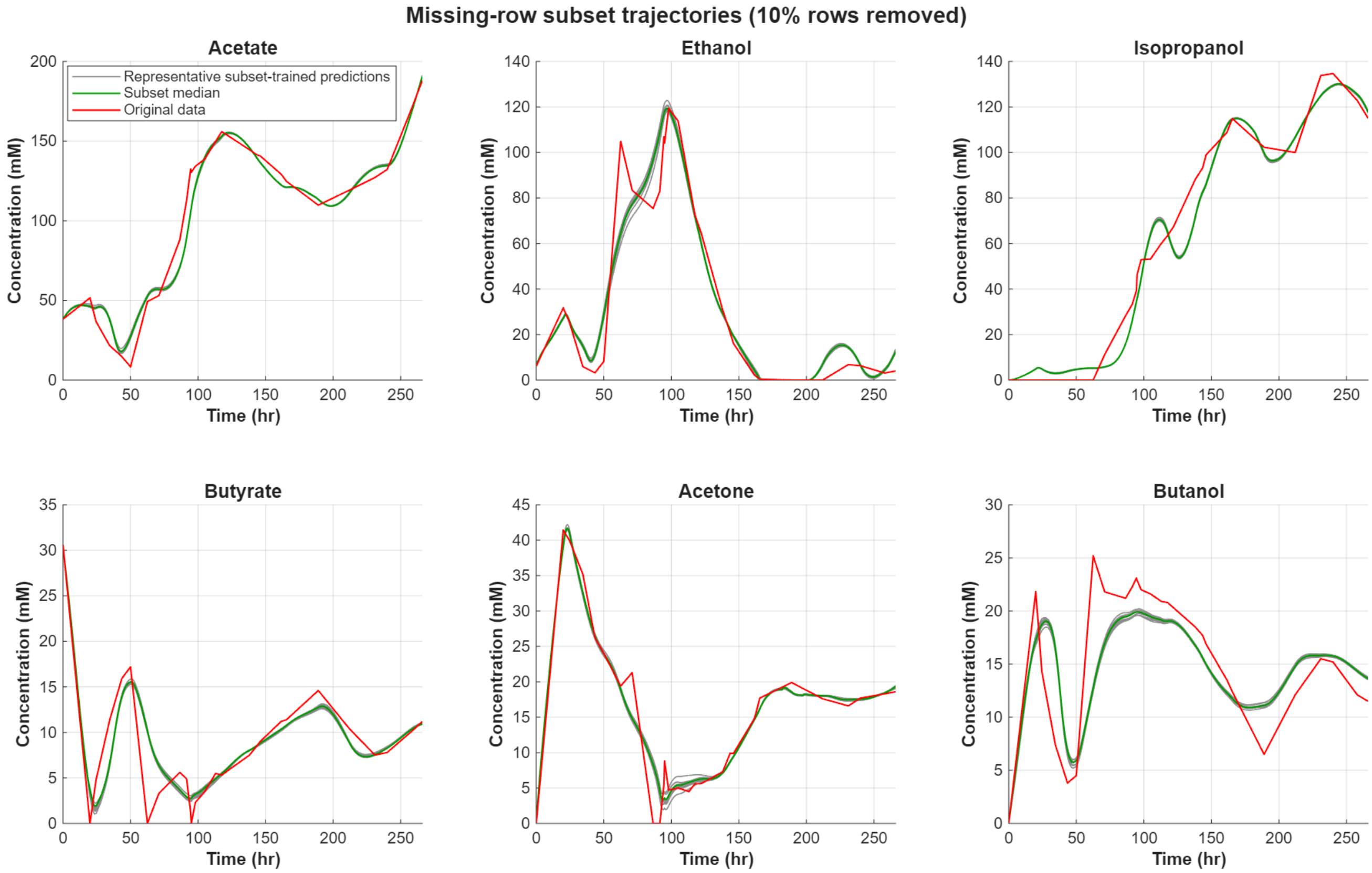

Figure C: Trajectory-level predictions from subset-trained models after randomly removing 10% of the regression rows. The red curves denote the original experimental trajectories, gray lines show representative subset-trained predictions, gray shaded regions indicate the 5th–95th percentile range across 100 trials, and green curves represent the median prediction.

library construction, and equation-specific structural masks used in the final model were applied across the tested λ values.

The selected value of $\lambda = 0.25$ was not chosen as an isolated optimum. Instead, it was selected from a stable threshold region where the identified sparse structure and trajectory-level performance remained nearly unchanged. Across $\lambda = 0.001$–0.25, the identified equations retained the same set of SINDy terms with negligible differences in the coefficients.

Even at stricter thresholds, $\lambda = 0.5$ and 1.0, the SINDy term retention rate was 96.77%. The trajectory-level metrics were also computed for the comparison. For $\lambda = 0.5$ and 1.0, the mean RMSE was 5.2948 and the mean R^2 was 0.8747, compared with 5.3004 and 0.8734 for the final $\lambda = 0.25$ model.

These results indicate that the final SINDy structure was not highly sensitive to the exact choice of λ. Therefore, the selected threshold was interpreted as a representative value within a structurally stable sparsity regime rather than as an over-tuned hyperparameter.

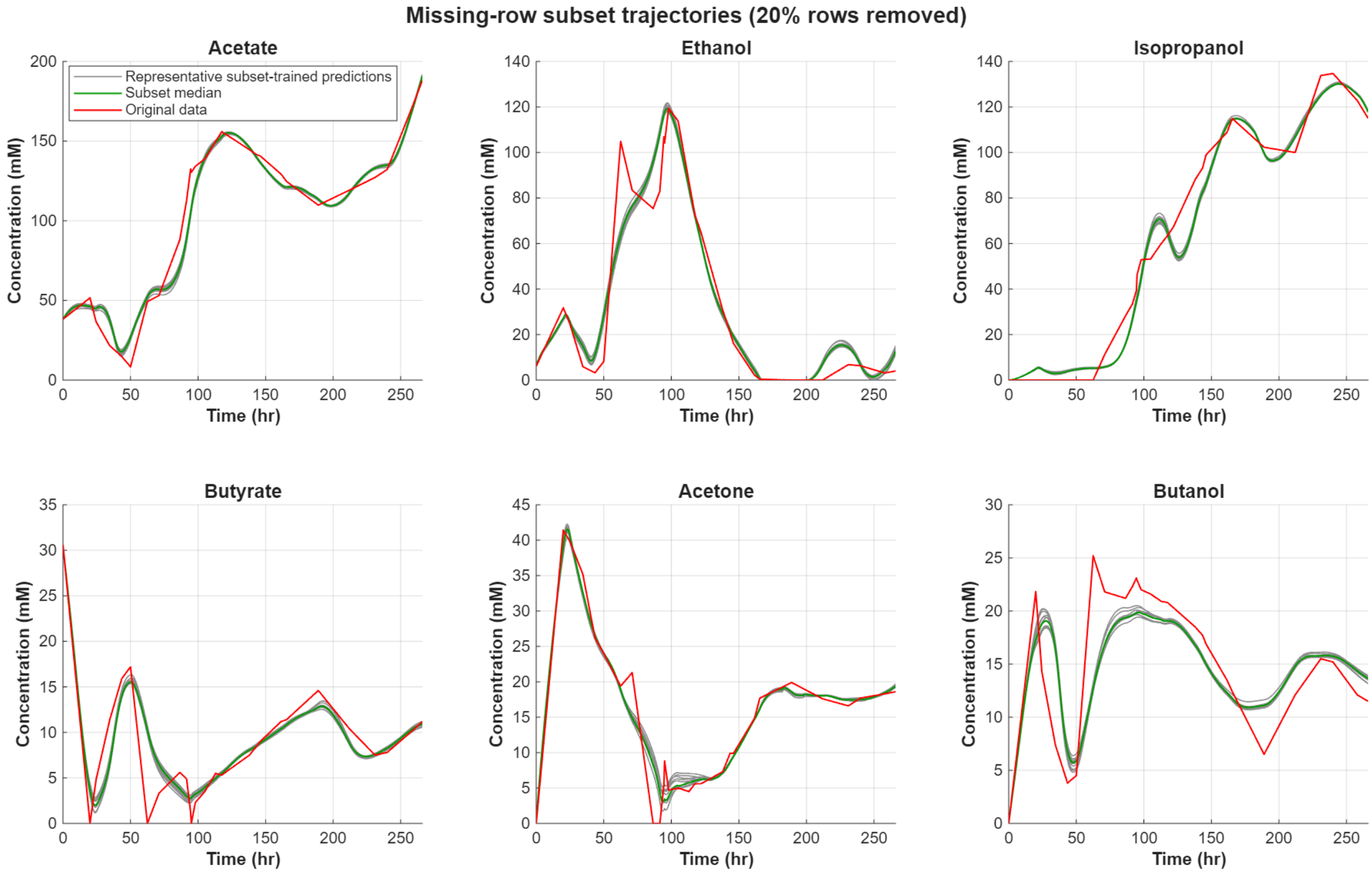

Figure D: Trajectory-level predictions from subset-trained models after randomly removing 20% of the regression rows. The red curves denote the original experimental trajectories, gray lines show representative subset-trained predictions, gray shaded regions indicate the 5th–95th percentile range across 100 trials, and green curves represent the median prediction.

1.5. Comparison with reduced control models

To evaluate whether the full physiology-informed SINDy structure is necessary, we compared the proposed model with a set of reduced control models, M0–M3. These models were constructed with progressively increasing levels of biological information while using the same preprocessing, transport-term subtraction, smoothing, derivative estimation, and STLS-Ridge identification procedure. Each model has the following configuration:

- M0: the simplest baseline model with transport-term and constants,
- M1: extracellular metabolite and pH-related variables along with M0 terms,
- M2: active-biomass-associated terms with M1 terms,
- M3: the full proposed model including active, dormant, and stress-related terms.

Note that M0 does not correspond to $\Theta(\mathbf{x}) = 0$. In M0, the unknown biological reaction term R_j is approximated by a constant offset. The overall comparison procedure is also illustrated

in Fig. E, and Table D summarizes which candidate-feature groups were included in the SINDy library for M0–M3. The fixed first-principles transport term, $D_e\,(S_{j,in} - S_j)$, was applied to all models before sparse regression and was not counted as a fitted SINDy candidate.

Table D: Candidate-feature groups included in the reduced SINDy models M0–M3.

Feature group	Representative terms	M0	M1	M2	M3
Constant offset in $R_j(\mathbf{x})$	1	✓	✓	✓	✓
Extracellular metabolites	S_{glu}, S_{fru}, S_{ace}, S_{acn}, S_{EtOH}, S_{IPA}, S_{BuOH}, S_{buty}	×	✓	✓	✓
pH-derived acidity term	$[H^+] = 10^{-pH+6}$	×	✓	✓	✓
Active biomass terms	$X_{C_{ac}}$, $X_{C_{lj}}$	×	×	✓	✓
Biomass-coupled active growth terms	$\mu_{C_{ac}} X_{C_{ac}}$, $\mu_{C_{lj}} X_{C_{lj}}$	×	×	✓	✓
Dormant biomass terms	$X_{C^*_{ac}}$, $X_{C^*_{lj}}$	×	×	×	✓
Stress-modulated metabolic-shift terms	$S_j X/(1 + P_{mix})$, $S_j X P_{mix}/(1 + P_{mix})$	×	×	×	✓

✓: included / ×: excluded

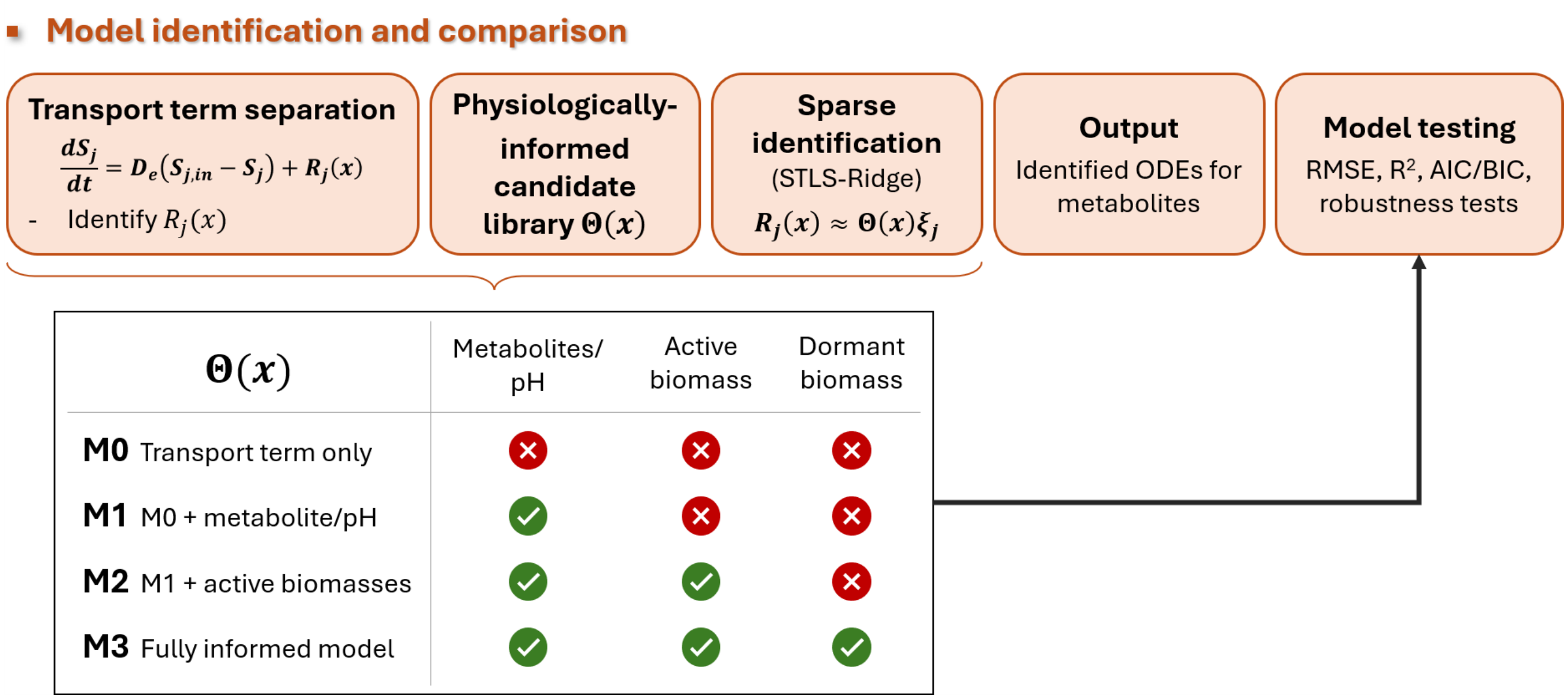

Figure E: The comparison between the proposed SINDy-identified models, from M0 to M3.

Fig. F shows the trajectory-level comparison of the reduced models for all metabolites. The simpler models captured some broad trends but showed larger deviations from the experimental trajectories, particularly for metabolites with phase-dependent production and consumption patterns. The inclusion of active-biomass terms improved the agreement substantially, while the full M3 model provided the most consistent reconstruction across the six extracellular metabolites.

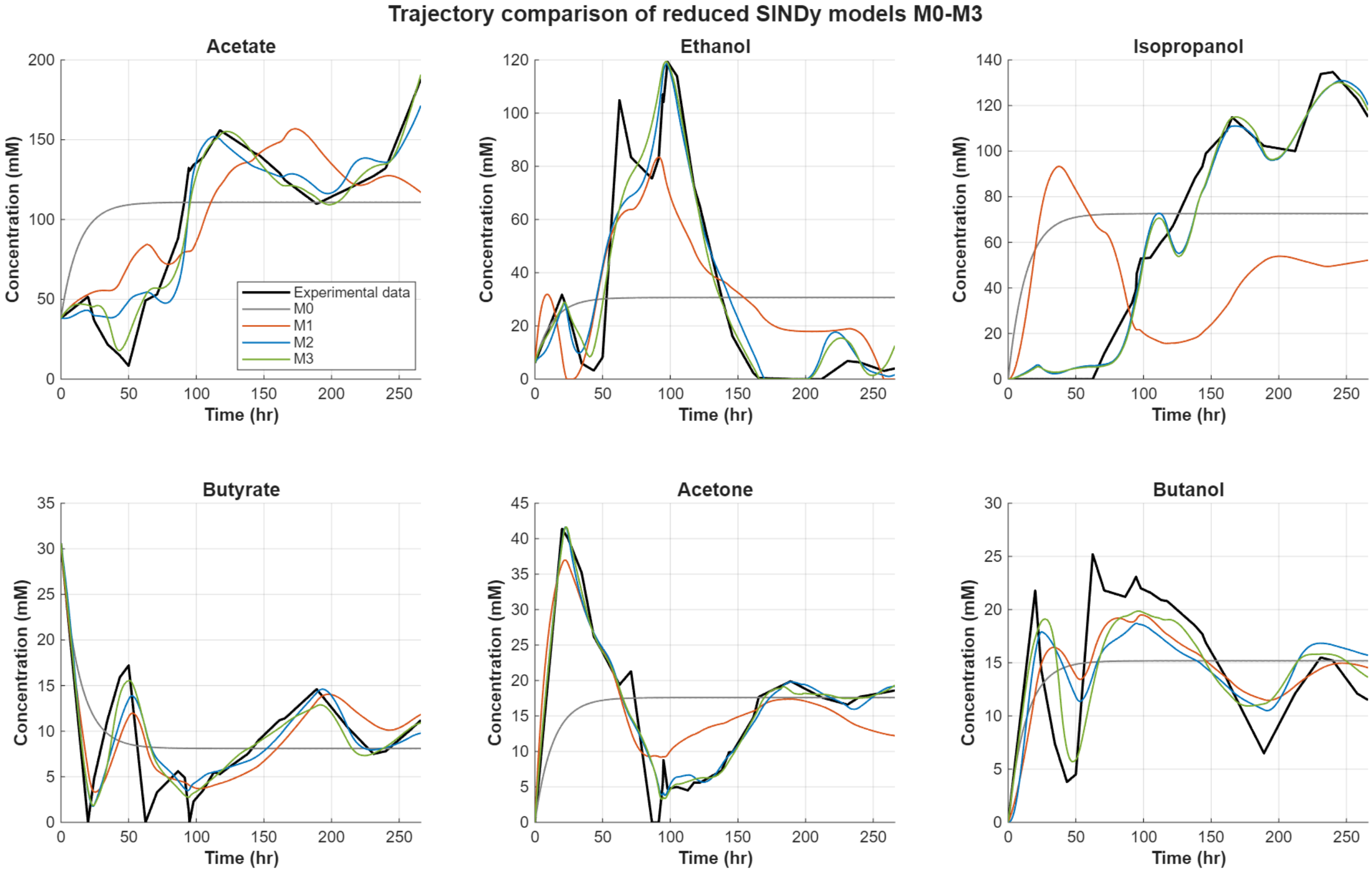

Figure F: Trajectory-level visual comparison of reduced SINDy models M0–M3. Black solid curves denote the experimental metabolite trajectories, while the colored solid curves represent model reconstructions from M0, M1, M2, and M3. Quantitative RMSE, R^2, AIC, and BIC comparisons are summarized separately in Table E.

Table E: Summary of reduced-model comparison metrics for M0–M3.

**Model**	**Description**	**Mean RMSE**	**Mean R^2**	**Total AIC**	**Total BIC**
M0	Baseline model	24.22	0.0844	23216.39	23216.39
M1	Metabolite/pH	20.82	0.417	15530.58	15590.48
M2	Active-biomass-informed	6.77	0.817	10753.72	10856.41
M3	Full physiology-informed	5.30	0.873	9582.11	9714.75

The quantitative comparison is summarized in Table E. The models were evaluated using trajectory-level RMSE and R^2, together with AIC and BIC to account for model complexity. The fixed first-principles transport terms were not counted as fitted SINDy parameters as they were specified from the perfusion mass balance. Overall, M3 achieved the best balance between trajectory agreement and complexity-penalized performance, supporting the inclusion of the full physiology-informed library in the final model.

These results indicate that the added biological structure is not merely increasing model flexibility, but improves the representation of the observed coculture metabolite dynamics. In particular, the improvement from M1 to M2 supports the importance of biomass-associated

terms, while the further improvement from M2 to M3 supports the practical contribution of dormant- and stress-related terms at the overall model level.

From a practical modeling perspective, the M2 model suggests that an active-biomass-informed SINDy library can provide a useful reduced alternative when direct dormant-state information is not included in the candidate library. However, M2 should not be interpreted as an OD_{600}-only model in the present analysis. The active biomass trajectories, $X_{C_{ac}}$ and $X_{C_{lj}}$, were still obtained using the rRNA-FISH/flow cytometry-constrained population estimates. Thus, when rRNA-FISH/flow cytometry data are unavailable, applying an M2-type library would require an additional approximation of the active biomass fractions from total OD_{600}, growth-phase information, or another population-state estimation method. Without such an approximation, the model would be closer to the M1 metabolite/pH-informed case than to the M2 active-biomass-informed case.

1.6. Leave-one-term-out analysis for Eqs. 15–16

To further investigate whether the retained variables in the final SINDy equations were justified, we performed a focused leave-one-term-out analysis for Eqs. 15–16. These equations correspond to the acetone and isopropanol dynamics, which have the largest number of SINDy-identified terms. Starting from the final M3 candidate sets, each retained term was removed one at a time, and the reduced model was refitted using the same preprocessing, transport-term subtraction, candidate-library construction, equation-specific structural masks, and STLS-Ridge procedure.

Each leave-one-term-out model was compared with the full selected equation using RMSE, AIC, and BIC. The fixed first-principles transport terms were not counted as fitted SINDy parameters because they were specified directly from the perfusion mass balance. Thus, this analysis tested whether individual SINDy-selected biological reaction terms contributed meaningfully to the trajectory fit and complexity-penalized model quality. The performance metrics are summarized in Tables F–G.

These results highlight that removing individual retained terms from Eqs. 15–16 consistently degraded the trajectory fit and worsened the complexity-penalized criteria. Therefore, the retained variables in the acetone and isopropanol equations were not included solely as redundant complexity, but they provided measurable contributions to the identified dynamics. This focused analysis supports the inclusion of the retained terms and provides an equation-level parsimony check for the final SINDy structures.

2. Additional operating-regime assessment using an additional dataset

We additionally examined an independent perfusion dataset as a possible external assessment case [1]. This dataset was obtained under a different operating regime from the primary

Table F: Summary of leave-one-term-out analysis for Eq. 15.

**Term**	**RMSE**	**AIC**	**BIC**
Eq. 15	1.82	650.24	680.19
$[H^+]$	4.21	1538.40	1551.24
$\mu_{C_{ac}} X_{C_{ac}}$	7.62	2174.36	2195.75
$\mu_{C_{lj}} X_{C_{lj}}$	4.15	1526.25	1547.64
$X_{C_{ac}}$	6.22	1955.61	1972.73
$X_{C^*_{lj}}$	4.34	1570.40	1583.24
Inhibition	4.21	1541.19	1558.30

Table G: Summary of leave-one-term-out analysis for Eq. 16.

**Term**	**RMSE**	**AIC**	**BIC**
Eq. 16	7.50	2157.84	2179.23
$\mu_{C_{lj}} X_{C_{lj}}$	11.75	2632.32	2645.16
$X_{C_{lj}}$	22.49	3326.51	3343.62
$X_{C^*_{lj}}$	9.58	2417.09	2434.20
Inhibition	10.37	2501.44	2518.55

manuscript dataset, including higher glucose loading, an early C_{ac}-dominant phase, a pronounced ethanol accumulation phase, and operational discontinuities associated with changes in the process configuration. Therefore, this dataset was not treated as a strict parameter-free external validation of the original M3 coefficients. Instead, it was used as an additional operating-regime assessment to determine whether the same physiologically informed SINDy library and equation-specific structural masks could identify comparable model structures under a distinct coculture condition.

As the dataset contained clear discontinuities, the available trajectory was divided into three continuous segments before SINDy identification: 0.0–29.0 h, 29.5–41.0 h, and 41.1–64.5 h. The same preprocessing, transport-term subtraction, candidate library construction, and STLS-Ridge sparse regression procedure used for the primary manuscript dataset were then applied within each segment. Only the SINDy coefficients were reestimated for this additional dataset, while the candidate library and equation-specific masks were kept unchanged.

The equation obtained from the independent perfusion operation is given below:

$$\begin{aligned}
\frac{dS_{ace}}{dt} =& -0.075S_{ace} + 3.898 + 0.4523[H^+] - 0.0986\mu_{C_{ac}}X_{C_{ac}} \\
& -0.2075X_{C_{ac}} - 0.0702X_{C^*_{ac}} \qquad \text{(M3 model)} \\
\frac{dS_{ace}}{dt} =& -0.083S_{ace} + 6.689 + 2.490[H^+] - \underbrace{20.365\mu_{C_{ac}}X_{C_{ac}}}_{\text{High initial } C_{ac}} \\
& -0.3396X_{C_{ac}} - 0.3114X_{C^*_{ac}} \qquad \text{(New experiment)}
\end{aligned} \tag{A}$$

$$\begin{aligned}
\frac{dS_{buty}}{dt} =& \; 0.075\,(30 - S_{buty}) - 1.127 + 0.0228[H^+] - 0.196\mu_{C_{ac}}X_{C_{ac}} \\
& -0.0425S_{BuOH} \qquad \text{(M3 model)} \\
\frac{dS_{buty}}{dt} =& \; 0.083\,(30 - S_{buty}) + 8.777 + 2.186[H^+] + 0.3391\mu_{C_{ac}}X_{C_{ac}} \\
& -0.2446S_{BuOH} - \underbrace{0.0342S_{glu}}_{\text{High initial } S_{glu}} \qquad \text{(New experiment)}
\end{aligned} \tag{B}$$

$$\begin{aligned}
\frac{dS_{EtOH}}{dt} =& -0.075S_{EtOH} + 16.71 + 0.241\mu_{C_{ac}}X_{C_{ac}} - 0.0376X_{C_{ac}} \\
& -0.0557X_{C^*_{ac}} - 0.0262S_{glu} - 0.108S_{ace} - 0.219S_{acn} \qquad \text{(M3 model)} \\
\frac{dS_{EtOH}}{dt} =& -0.083S_{EtOH} + \underbrace{129.7}_{\text{High initial } S_{EtOH}} + 0.888\mu_{C_{ac}}X_{C_{ac}} + 0.0929X_{C_{ac}} \\
& -2.707X_{C^*_{ac}} - 0.0470S_{glu} - 0.660S_{ace} + 1.171S_{acn} \qquad \text{(New experiment)}
\end{aligned} \tag{C}$$

$$\begin{aligned}
\frac{dS_{BuOH}}{dt} =& -0.075S_{BuOH} + 1.749 - 0.0227S_{buty} - 0.0502\left[H^+\right] \qquad \text{(M3 model)} \\
\frac{dS_{BuOH}}{dt} =& -0.083S_{BuOH} + \underbrace{11.44}_{\text{High initial } S_{EtOH}} - 0.240S_{buty} - 2.668\left[H^+\right] \qquad \text{(New experiment)}
\end{aligned} \tag{D}$$

$$\begin{aligned}
\frac{dS_{acn}}{dt} =& -0.075S_{acn} + 0.892 + 0.0250\left[H^+\right] - 0.128\mu_{C_{ac}}X_{C_{ac}} - 0.031\mu_{C_{lj}}X_{C_{lj}} \\
& + 0.0103X_{C_{ac}} - 0.226X_{C^*_{lj}} - 0.0404X_{C^*_{lj}}\frac{S_{acn}}{1 + P_{mix,C_{lj}}} \qquad \text{(M3 model)} \\
\frac{dS_{acn}}{dt} =& -0.083S_{acn} - 4.315 + 9.277\left[H^+\right] - 0.941\mu_{C_{ac}}X_{C_{ac}} - \underbrace{28.47\mu_{C_{lj}}X_{C_{lj}}}_{\text{Low } C_{lj} \text{ level}} \\
& -0.0867X_{C_{ac}} + 0.270X_{C^*_{lj}} + \underbrace{0.496X_{C^*_{lj}}\frac{S_{acn}}{1 + P_{mix,C_{lj}}}}_{\text{Low } C_{lj} \text{ level}} \qquad \text{(New experiment)}
\end{aligned} \tag{E}$$

$$
\begin{aligned}
\frac{dS_{IPA}}{dt} =& -0.075S_{IPA} + 0.0192S_{acn} + 0.0196\mu_{C_{lj}}X_{C_{lj}} + 0.0291X_{C_{lj}} \\
& + 0.171X_{C^*_{lj}} - 0.0396X_{C^*_{lj}}\frac{S_{acn}}{1+P_{mix,C_{lj}}} \qquad \text{(M3 model)} \\
\frac{dS_{IPA}}{dt} =& -0.083S_{IPA} + 0.0170S_{acn} - 2.571\mu_{C_{lj}}X_{C_{lj}} - 0.0806X_{C_{lj}} \\
& \underbrace{+ 0.722X_{C^*_{lj}} + 0.511X_{C^*_{lj}}\frac{S_{acn}}{1+P_{mix,C_{lj}}}}_{\text{Low } C_{lj} \text{ level}} \qquad \text{(New experiment)}
\end{aligned} \tag{F}
$$

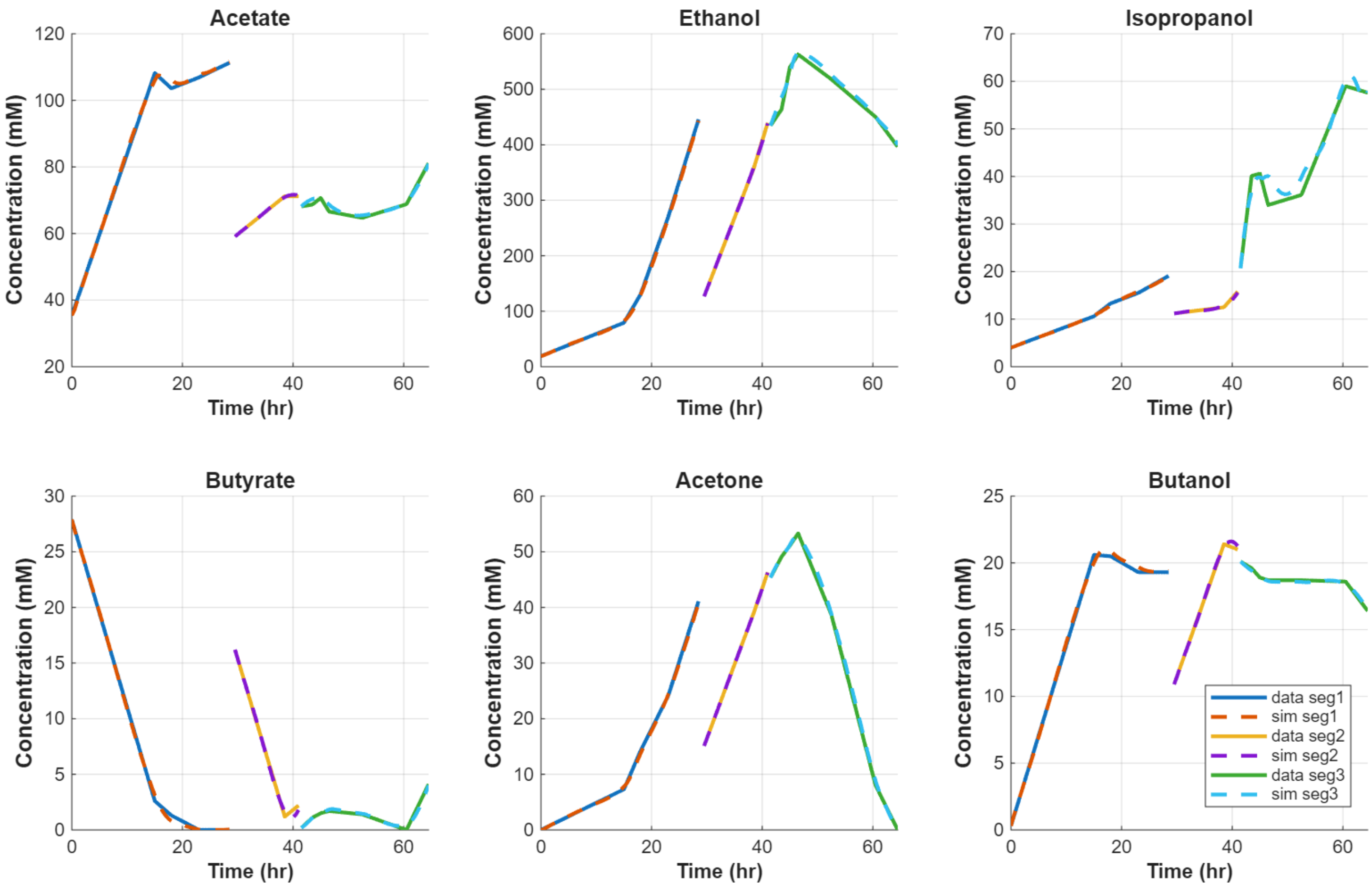

Figure G: Segment-wise reconstruction of the additional perfusion dataset using the same SINDy candidate library and equation-specific structural masks as the primary manuscript model. The dataset was divided into three continuous segments to exclude operational discontinuities before coefficient reestimation.

The segment-wise reestimated models showed that the same library and structural masks could generate models that qualitatively reproduced the local metabolite trends in the additional dataset, as presented in Fig. G. The equation-level comparison shows that the additional dataset retained many of the same functional forms selected in the original M3 model, including dilution terms, pH-related terms, biomass-associated terms, and metabolite-interaction terms, despite the substantial differences in operating conditions. This suggests that the proposed candidate library captures physiologically relevant relationships that can also be selected under a distinct coculture regime. However, the reestimated coefficients differed from those obtained from the

primary manuscript dataset, particularly for the ethanol-, isopropanol-, and acetone-related dynamics. In some cases, the magnitudes and the signs of the coefficients changed, reflecting the distinct operating condition rather than direct coefficient transferability.

These results indicate that the proposed SINDy library structure is applicable to a distinct operating regime, but the original coefficient values should not be interpreted as universal kinetic constants transferable across all process conditions. Rather, the fitted coefficients should be interpreted as regime-specific effective kinetic closures. Accordingly, this additional dataset supports the structural consistency of the proposed modeling framework, while strict external validation of the original coefficients will require additional independent coculture runs collected under comparable or systematically varied operating conditions with complete raw measurements.

References

[1] Otten JK. Synthetic and Syntrophic Cocultures of Clostridia Enable Enhanced Production of Three-to-Six Carbon Chemicals Through Carbon Capture, Cell Fusion, and Metabolite Sharing Across Fermentation Scales. University of Delaware; 2025.
