# Supplementary material for: From observable fermentation data to hidden cell states: A modeling study of a mixotrophic Clostridium coculture under perfusion mode
Source: PLoS Comput Biol. 2026 Sep 28;22(9):e1014759. doi: 10.1371/journal.pcbi.1014759 (PMC13619069; doi:10.1371/journal.pcbi.1014759)
Supplement: Supplementary file 2 [file pcbi.1014759.s003.pdf]

List of changes

The items that have been changed are:

1. We revised the manuscript to tone down the claim level and clarify the scope of the model-supported interpretations.
2. The Abstract was revised.
3. A schematic diagram was included as new Fig. 1.
4. Captions were revised: Table 1, Figure 3 (previously Figure 2), and Figure 4 (previously Figure 3).
5. Line 109-110: An additional statement is added regarding the replicate analysis.
6. Line 182-187: A brief clarifying sentences for the dormant state were added.
7. Line 191: We added 'reactivation' within the mechanism explanation.
8. Line 197-203: The description of population measurement and conversion was added.
9. Line 223-249: We relocated the detailed explanation about P_{mix} term near Eq. 4.
10. Line 258-259: We added the discussion regarding Hill coefficient.
11. Line 265-271: We added the clarifying statement regarding cell death.
12. Line 327-339: We added the explanation for the STLS-Ridge method.
13. Line 374-380: The discussion regarding the dormant population was added.
14. Line 467-473: The description of first-principles terms was added.
15. Line 612-616: Discussion was extended regarding the model performance.
16. Line 444-449; Line 581-585: We added a guiding sentence to SI for interested readers.
17. Supporting Information: An additional PDF file is attached for the detailed analysis results, including:
    (a) Trajectory-level performance metrics
    (b) Model robustness tests
        i. Noise-injection experiments
        ii. Missing-data subset robustness analysis
        iii. Hyperparameter sensitivity analysis
        iv. Comparison with reduced control models
        v. Leave-one-term-out analysis
    (c) Additional operating-regime assessment

Reply to Reviewer 1

A detailed reply to the reviewer's comments is given below:

1. **The current analysis lacks a thorough investigation into data sensitivity. The authors should perform noise-injection experiments and sensitivity analyses to determine how measurement noise impacts model predictions.**

   We thank the reviewer for the suggestion. To examine how measurement uncertainty affects the identified model, we have added a noise-injection robustness analysis. The results are summarized below, and the detailed discussion including graphics are included in Section 1.2 of the SI.

   In this analysis, Gaussian noise was added directly into the extracellular metabolite measurements at 5% and 10% of each metabolite's dynamic range, and the full identification workflow was repeated for 100 trials at each noise level.

   The resulting noise-trained models were evaluated against the original full trajectory dataset using RMSE, R^2, the number of selected terms, and the retention rate of the terms selected in the full-data model. The full-data model gave a mean RMSE of 5.3004 and a mean R^2 of 0.8734. With 5% injected noise, the mean RMSE was 6.0275 $\pm$ 0.0873 and the mean R^2 was 0.8605 $\pm$ 0.0029. With 10% injected noise, the mean RMSE was 7.0823 $\pm$ 0.1735 and the mean R^2 was 0.8318 $\pm$ 0.0073.

   Although the trajectory error increased as expected when measurement noise was included, the selected SINDy structure was largely preserved, with selected-term retention rates of 99.43% and 99.08% for the 5% and 10% noise cases, respectively. These results indicate that the identified model is robust to moderate measurement noise.

2. **Address Missing Data: Study the effect of missing values and outliers by sampling subsets of the data.**

   We thank the reviewer for the comment. To test whether the identified equations depend strongly on a small subset of time points, we have added a missing-data subset analysis. Unlike the noise-injection analysis, this analysis kept the original measured values unchanged but randomly removed portions of the regression rows before model identification.

   To keep the preprocessing consistent with the final model, the full dataset was first processed using the same smoothing, derivative-estimation, and library-construction procedure. Random subsets were then generated by removing 10% and 20% of the regression rows before STLS-Ridge identification, while keeping the same candidate libraries and hyperparameter settings. Each subset-trained model from the 100 repeated random-removal trials was evaluated against the full trajectory dataset for the extracellular metabolites.

To sum up the result, the full-data model gave a mean RMSE of 5.3004 and a mean R^2 of 0.8734. After random removal of 10% of the regression rows, the mean RMSE was increased to 5.3081 ± 0.0350 and the mean R^2 became 0.8735 ± 0.0025. After random removal of 20% of the regression rows, the mean RMSE was 5.3278 ± 0.0534 and the mean R^2 was 0.8721 ± 0.0040. The selected-term retention rates were 99.28% and 98.34% for the 10% and 20% removal cases, respectively. These results highlight that the identified equations were not driven by a small number of individual regression rows. The corresponding results are summarized in Section 1.3 of the SI.

3. **Evaluate Parameter Sensitivity: Provide a sensitivity analysis for the parameters in Table 1 (e.g., how a 10% variation impacts the fit, feature selection, or error rates).**

   We thank the reviewer for the considerate comment. The parameters listed in Table 1 are fitted parameter estimates obtained from the experimental dataset, rather than independently prescribed constants. Thus, perturbing one fitted parameter while holding all other fitted quantities fixed would not represent a consistent re-identification analysis, because these parameters were jointly estimated and may compensate for one another.

   For this reason, we addressed the underlying sensitivity concern through perturbations that are directly connected to the identification workflow. Specifically, the noise-injection analysis in the Comment 1 tests the effect of measurement uncertainty, while the missing-data subset analysis in the Comment 2 evaluates the dependence of the identified equations on individual time points. These analyses quantify the effects of data perturbations on trajectory-level RMSE, R^2, selected-term retention, and the number of selected terms.

   In addition, the reduced-library comparison, AIC/BIC analysis, and leave-one-term-out analysis provide complementary checks on model complexity and variable inclusion. Together, these analyses provide a practical sensitivity assessment of fit quality, feature selection, and error metrics without introducing an inconsistent one-at-a-time perturbation of jointly fitted parameters. For the detail regarding these, please refer to our responses to the Comments 1, 2, 5, 6, and 11.

4. **Replicate Analysis: Clarify whether the experimental data used are biological or technical replicates, and how many were used. The model should be evaluated using individual replicates rather than just averaged data to account for experimental variance.**

   We thank the reviewer for the kind suggestion. The experimental data were obtained from single bioreactor operation without biological replicates. The information has been added to the Materials and Methods section as follows:

"The dataset used in this modeling study was obtained from a single perfusion bioreactor operation and did not include independent biological replicate runs."

It should be noted that controlling the mixotrophic perfusion fermentation mode (continuous + cell retention + CO_2/H_2 gas injection) for the C_{ac}-C_{lj} coculture at the 3.6-L scale is not trivial, which demands well-established process control strategies for obtaining precise fermentation data. We anticipate that this study will help develop the control strategies for precise coculture fermentation.

5. **Benchmark Against Baseline and Other models: Compare the proposed model with purely data-driven methods and hybrid models.**

We thank the reviewer for this insightful comment. In order to address this point, we have added a stepwise comparison of reduced SINDy libraries to evaluate the proposed model against simpler baseline formulations. Rather than constructing an entirely separate population-balance model, which would introduce a different set of kinetic assumptions, we have compared the models at the SINDy identification layer using the same preprocessing, transport term, and STLS-Ridge procedure.

Specifically, we considered four models: M0, a transport-only model without a learned biological reaction term; M1, a metabolite/pH-only SINDy model using only extracellular observable variables; M2, a reduced SINDy model including extracellular variables and active-biomass terms but excluding all dormant-state terms; and M3, the proposed full physiologically informed library including active, dormant, and stress-related terms. For each reduced library, the STLS-Ridge regression was rerun.

The comparison showed that the transport-only model and metabolite/pH-only model were insufficient to reproduce the observed metabolite trajectories. Adding active-biomass terms substantially improved the model performance, reducing the mean RMSE from 20.82 in M1 to 6.77 in M2 and increasing the mean R^2 from 0.417 to 0.817. The full physiologically informed library further improved the overall performance, reducing the mean RMSE to 5.30 and increasing the mean R^2 to 0.873. These results indicate that biomass-informed terms are necessary to capture the extracellular metabolite dynamics and that the full physiologically informed library provides the best overall trajectory-level performance. We have added this comparison to Section 1.5 of the SI.

6. **Complexity Penalization: Use information criteria such as AIC or BIC to justify model complexity and evaluate stepwise models.**

We thank the reviewer for clarifying questions. To account for model complexity, we evaluated the stepwise SINDy models using AIC and BIC. For each model, AIC and BIC were calculated from the residual sum of squares using the number of retained nonzero

SINDy terms as the complexity penalty. Please note that the fixed first-principles transport terms were not counted as fitted parameters as they were determined directly from the perfusion mass balance.

The total AIC and BIC values were obtained by summing the values across the metabolite equations. The full physiologically informed library (M3) achieved the lowest total AIC and BIC values despite retaining the largest number of terms. The total AIC decreased from 23216.39 (M0) to 15530.58 (M1), 10753.72 (M2), and 9582.11 (M3), while the total BIC decreased from 23216.39 (M0), to 15590.48 (M1), 10856.41 (M2), and 9714.75 (M3). Therefore, the additional physiological terms in M3 were supported even after penalizing model complexity. We have added this analysis to Section 1.5 of the SI.

7. **Report standard performance metrics, including MSE, R2, and AUC, particularly for hidden parameters such as dormant vs. active population ratios.**

We thank the reviewer for raising this important point. We have added trajectory-level performance metrics, including MSE, RMSE, MAE, R^2, and relative AUC error, for experimentally measured metabolites and experimentally constrained active-biomass trajectories. Because the model predicts continuous time-series trajectories rather than binary classes, ROC-AUC may not be directly applicable. Therefore, AUC was interpreted as the time-integrated area under each trajectory, and the relative AUC error was calculated as:

$$\text{Relative AUC error} = \frac{|\text{AUC}_{\text{model}} - \text{AUC}_{\text{exp}}|}{|\text{AUC}_{\text{exp}}|} \times 100\%.$$

For the final M3 model, the relative AUC errors were 2.81%, 0.09%, 0.15%, 3.24%, 2.39%, 0.07%, 0.07%, and 0.62% for active C_{ac}, active C_{lj}, acetate, ethanol, isopropanol, butyrate, acetone, and butanol, respectively.

We have summarized the corresponding MSE, RMSE, MAE, and R^2 values in Section 1.1 of the SI (For RMSE and R^2, please refer to our response to the comment 5), particularly in Table A. These metrics showed strong agreement for the experimentally measured or experimentally constrained trajectories, especially for active C_{ac}, active C_{lj}, acetate, ethanol, isopropanol, and acetone.

Although the pointwise R^2 values were lower for butyrate and butanol than for the other metabolites, the model still captured their overall temporal trends, including the depletion-reaccumulation pattern of butyrate and the rise-decline behavior of butanol. The lower R^2 values mainly reflect local discrepancies in sharp transient regions and peak amplitudes, rather than a failure to reproduce the overall trajectory behavior. This interpretation is supported by the small relative AUC errors for these metabolites, which were 0.07% for butyrate and 0.62% for butanol.

Overall, the combined metric analysis shows that the model captures the major coupled

metabolite dynamics while still reporting metabolite-specific differences in pointwise accuracy. In particular, the lower pointwise R^2 values for butyrate and butanol are interpreted together with their small relative AUC errors, rather than as a failure to capture the overall trajectory behavior.

For the active-dormant population analysis, performance metrics were reported only for experimentally measured or experimentally constrained quantities. Because dormant biomass was reconstructed as a latent model state, not as values that were measured independently, we did not report dormant-state error metrics against experimental dormant-biomass data. This distinction has been clarified in the revised manuscript:

"Accordingly, trajectory-level performance metrics were reported only for experimentally measured metabolites and experimentally constrained active-biomass trajectories."

8. **The most significant weakness is that the model is currently trained and assessed on the same dataset. The performance must be assessed on independent datasets or runs that include novel variations (e.g., differences in growth rates or initial compositions).**

We thank the reviewer for pointing this out. We examined an additional perfusion dataset as a possible independent assessment case. However, this dataset was obtained under a substantially different operating regime, including higher glucose loading, and early C_{ac}-dominant phase, a large ethanol spike, and operational discontinuities associated with process configuration changes. Therefore, we do not present it as a strict parameter-free validation of the original M3 coefficients.

The detailed results can be found in Section 2 of the SI. We used this dataset as an additional operating-regime assessment. Using the same physiologically informed SINDy library and equation-specific masks, we performed segment-wise reestimation after excluding discontinuous transitions. This analysis showed that the model structure could represent the local metabolite dynamics of the additional dataset, but the reestimated coefficients differed from those obtained from the primary manuscript dataset. These results suggest that the proposed library structure is transferable across operating regimes, whereas the fitted coefficients should be interpreted as regime-specific effective kinetic closures.

We have revised the manuscript to clarify this validation scope and to tone down claims about generalizability. The revised discussion now states that strict external validation will require additional independent coculture runs collected under comparable or systematically varied operating conditions with complete raw measurements.

We also respectfully note that the experimental dataset was generated as part of a prior experimental effort, and the Ph.D. student who primarily conducted the corresponding experiments has recently graduated. As a result, generating additional replicate or follow-up

perfusion datasets at this revision stage would be practically challenging. We therefore used the available additional dataset to provide the most rigorous assessment currently feasible. We hope the reviewers will consider this circumstance favorably.

9. **Hyperparameters (such as lambda) should be determined using a dedicated training set.**

   We thank the reviewer for the comment. We agree that, in general, hyperparameters should ideally be selected using a dedicated training/validation set. In the present study, however, the available primary dataset consists of a single continuous coculture perfusion experiment, and aggressive temporal splitting can remove important phase-dependent dynamics from the identification dataset. Therefore, rather than selecting λ by optimizing performance on a small held-out subset, we evaluated the sensitivity of the identified SINDy structure over a broad range of λ values.

   Specifically, we have performed a hyperparameter-sensitivity analysis for the STLS-Ridge threshold parameter λ and added the results to Section 1.4 of the SI. The same preprocessing, candidate libraries, and model-evaluation procedure were used while sweeping λ over 0.001–1.0. The analysis was performed for Eqs. 11-16, corresponding to acetate, butyrate, ethanol, butanol, acetone, and isopropanol.

   The final value $\lambda = 0.25$ lies within a stable threshold region. Across $\lambda = 0.001 - 0.25$, the identified equations were unchanged with all retained terms. That being said, $\lambda = 0.25$ was used as the final threshold value within this stable region, rather than as an isolated point estimate.

10. **Demonstrate model stability by showing whether the selected features or equations remain consistent when hyperparameters are varied.**

    We thank the reviewer for the comment. We evaluated the stability of the selected SINDy terms across the same λ-sweep analysis reported in response to Comment 9.

    Beyond we described in Comment 9, at stricter thresholds, $\lambda = 0.5$ and 1.0, the selected structures remained highly similar to the final model. In both cases, the SINDy term-retention rate was 96.77%.

    The trajectory-level metrics also changed only slightly across the tested range. For $\lambda = 0.5$ and 1.0, the mean RMSE was 5.2948 and the mean R^2 was 0.8747, compared with 5.3004 and 0.8734 for the final $\lambda = 0.25$ model. These results show that the identified SINDy structures are stable with respect to moderate changes in the STLS-Ridge threshold.

11. **The authors should justify the inclusion of each variable, particularly in equations 15 and 16, in terms of improvement in fit. Provide a series of fits with**

fewer variables to show the marginal gain of each additional parameter. If removing the least important variable reduces performance by only 1%, a simpler, more parsimonious model maybe preferable for generalization.

We thank the reviewer for the insightful comment. To evaluate the marginal contribution of variables in Eqs. 15-16, we performed a focused leave-one-term-out analysis and added the results to Section 1.6 of the SI.

Starting from the final M3 candidate sets for Eqs. 15-16, each retained term was removed one at a time, and the reduced model was refitted using the same preprocessing and STLS-Ridge procedure. Each model was then compared with the full selected equation using RMSE, AIC, and BIC.

For the acetone equation (Eq. 15), the full selected equation gave an RMSE of 1.82, AIC of 650.24, and BIC of 680.19. When one candidate was removed at a time, the RMSE increased to 4.15–7.62, while AIC and BIC increased to 1526.25–2174.36 and 1547.64–2195.75, respectively.

For the isopropanol equation (Eq. 16), the full selected equation gave an RMSE of 7.50, AIC of 2157.84, and BIC of 2179.23. The leave-one-term-out alternatives increased the RMSE to 9.48–22.49, AIC to 2347.09–3236.51, and BIC to 2423.12–3343.62.

Therefore, removing individual retained terms from Eqs. 15-16 consistently degraded the fit and worsened the complexity-penalized criteria. This focused analysis supports the inclusion of the retained variables in Eqs. 15-16 and provides an equation-level parsimony check for the identified SINDy structures. We have summarized this result in the SI for the readers' further reference.

12. **The "regulatory logic" described in the paper lacks prospective or causal validation. It is unclear if the identified relationships are causal or merely correlative. The authors should perform temporal or perturbation analyses to demonstrate that these learned relationships reflect actual regulatory mechanisms.**

We thank the reviewer for the comment. We have revised the manuscript to clarify the scope of the SINDy-based interpretation and to distinguish the present model-supported kinetic interpretation from prospective causal validation. This revision is closely linked to the broader claim-level adjustment described in our response to the Comment 14.

In the revised manuscript, the SINDy-identified relationships are presented as model-supported temporal associations rather than prospectively validated causal regulatory mechanisms. Accordingly, expressions such as 'regulatory logic' and 'governed by' were replaced where appropriate with more precise wording, including 'identified kinetic structure', 'model-supported association', and 'is consistent with'.

To address the temporal aspect of the reviewer's concern, the revised Discussion now emphasizes the time-resolved consistency among organic acid accumulation, active-dormant

population shifts, acetone turnover, and isopropanol formation. This temporal ordering supports the proposed model-based interpretation of stress-associated state transitions and interspecies metabolic exchange within the current dataset.

We have also added a statement that targeted perturbation experiments, such as controlled changes in organic acid loading, gas availability, or initial species composition, would provide a direct experimental route to test the causal roles of the identified interactions. Thus, the revised manuscript now more clearly defines the evidential basis of the inferred interactions while outlining how these hypotheses can be tested experimentally.

At the end of Results and Discussion section, the following statement has been added:

"The SINDy-identified structures provide a data-constrained kinetic interpretation of the observed coculture dynamics under perfusion operation. In particular, the temporal ordering of organic acid accumulation, active-dormant population shifts, acetone turnover, and isopropanol formation is consistent with a sequential interpretation of stress response and interspecies metabolic exchange. Future perturbation studies that vary organic acid loading, gas availability, or initial species composition would provide a direct experimental route to further test these inferred interactions and extend the present model-based interpretation."

13. **The abstract needs more specificity regarding exactly what is being predicted. The results are currently described at a superficial level.**

We thank the reviewer helpful comment. We have revised the abstract to more clearly specify what is predicted and reconstructed by the model. The revised abstract now explicitly states that the framework estimates active C_{ac} and C_{lj} biomass-equivalent trajectories, reconstructs dormant biomass as model-constrained latent states, predicts extracellular metabolite concentration profiles, and identifies sparse biological reaction terms through SINDy. We also revised the result description to focus on specific model-supported findings, including acid-associated active-dormant transitions and acetone-to-isopropanol conversion, rather than presenting the results at a broad or superficial level.

The revised abstract is as follows:

"Microbial cocultures exhibit complex population dynamics that are difficult to interpret because internal physiological states are only partially observable. In particular, active and dormant cell states can influence system-level behavior but are rarely resolved from standard fermentation measurements. In this study, we present a hybrid modeling framework that combines structured population-state reconstruction with sparse identification of nonlinear dynamics (SINDy) to analyze a Clostridium acetobutylicum-Clostridium ljungdahlii coculture under perfusion mode. The framework estimates active C_{ac} and C_{lj} biomass-equivalent trajectories from observable biomass and activity measurements, reconstructs dormant populations as model-constrained latent states, and uses these states to identify

extracellular metabolite dynamics. After accounting for first-principles perfusion transport, SINDy identified sparse biological reaction terms associated with organic acid turnover, solvent formation, and acetone-to-isopropanol conversion. The resulting model captured active-biomass and metabolite trajectories and suggested that the coculture dynamics are consistent with acid-associated state transitions and sequential metabolic exchange. This framework provides a transparent strategy for interpreting partially observed microbial co-cultures while explicitly treating dormant biomass as a latent reconstructed state."

14. **Given that the model has not been rigorously evaluated for generalizability, the claims made throughout the paper should be significantly toned down. This applies to both the abstract and the conclusion; the latter should focus more on current limitations than broad potential.**

    We thank the reviewer for this important comment. We have revised the manuscript to make the scope of the conclusions more precise and consistent with the validation performed in this study. Please note that this revision is linked to the causal-interpretation issue raised in Comment 12.

    Across the abstract, Results and Discussion, and Conclusion, we revised stronger mechanistic wording such as 'reveals', 'governed by', and 'regulatory logic' to more precise expressions such as 'suggests', 'is consistent with', and 'model-supported association'.

    The revised Conclusion now focuses on the model-supported findings, including active-biomass reconstruction, extracellular metabolite prediction, and SINDy-identified kinetic structures under the present operating conditions. It also states the remaining validation needs, including the need for independent coculture experiments, replicate-level datasets, and targeted perturbation studies to further assess generalizability and causal interpretation.

15. **Figures should include simple schematics explaining the models, illustrating the relationship between input data, the governing equations, and the resulting outputs. Visual comparisons with simpler control models should also be included.**

    We thank the reviewer. To help readers understand the overall workflow without an extra effort, we have included a simple schematic explaining the model identification process. Please see the attached schematic below: This figure is included as Fig. 1 in the revised manuscript.

    In addition, to provide a visual comparison with our simpler models, we have added the following illustration with the SI, as shown below: This figure is included as Fig. E in the SI.

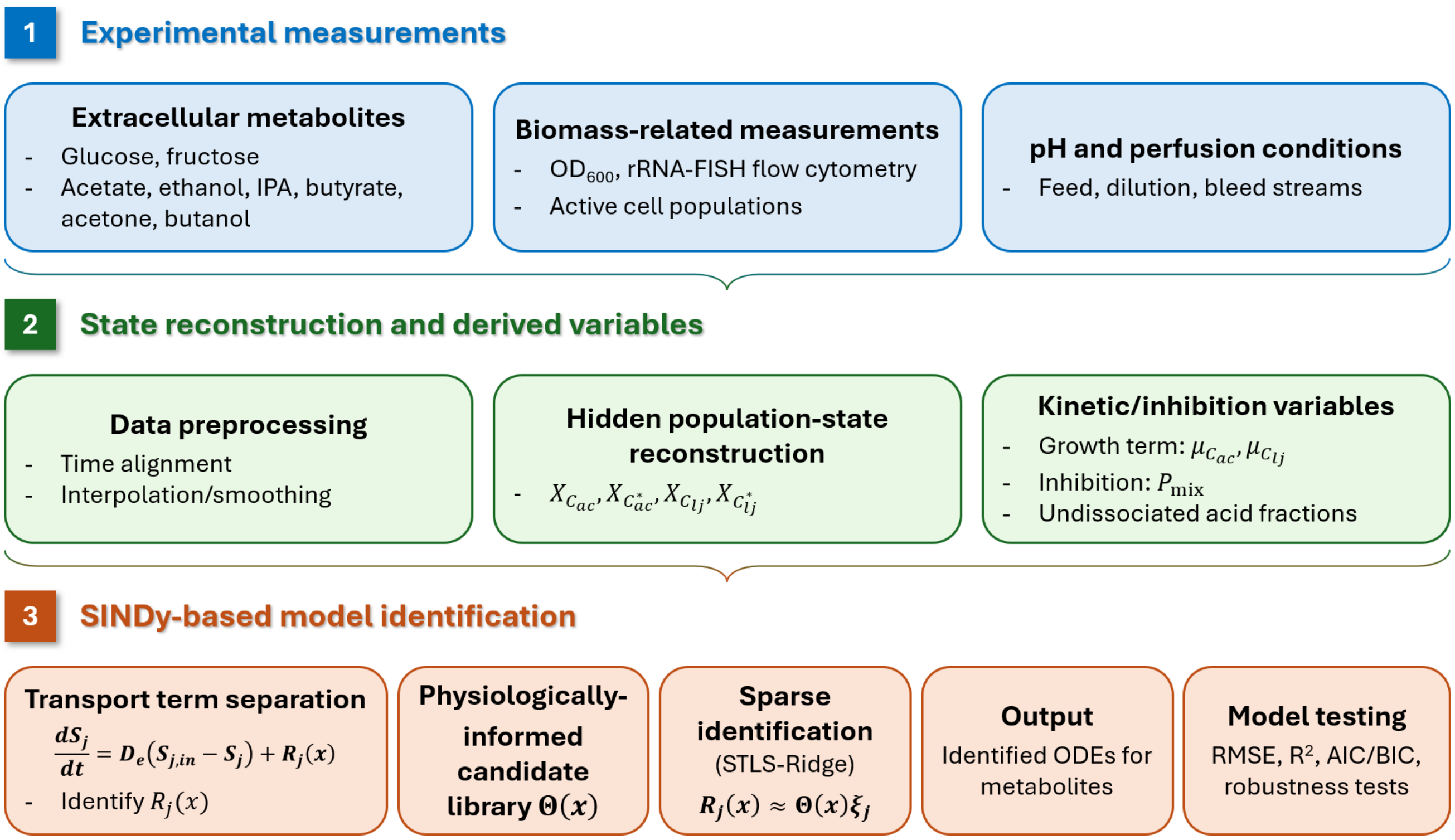

Figure 1: Overview of the proposed modeling framework.

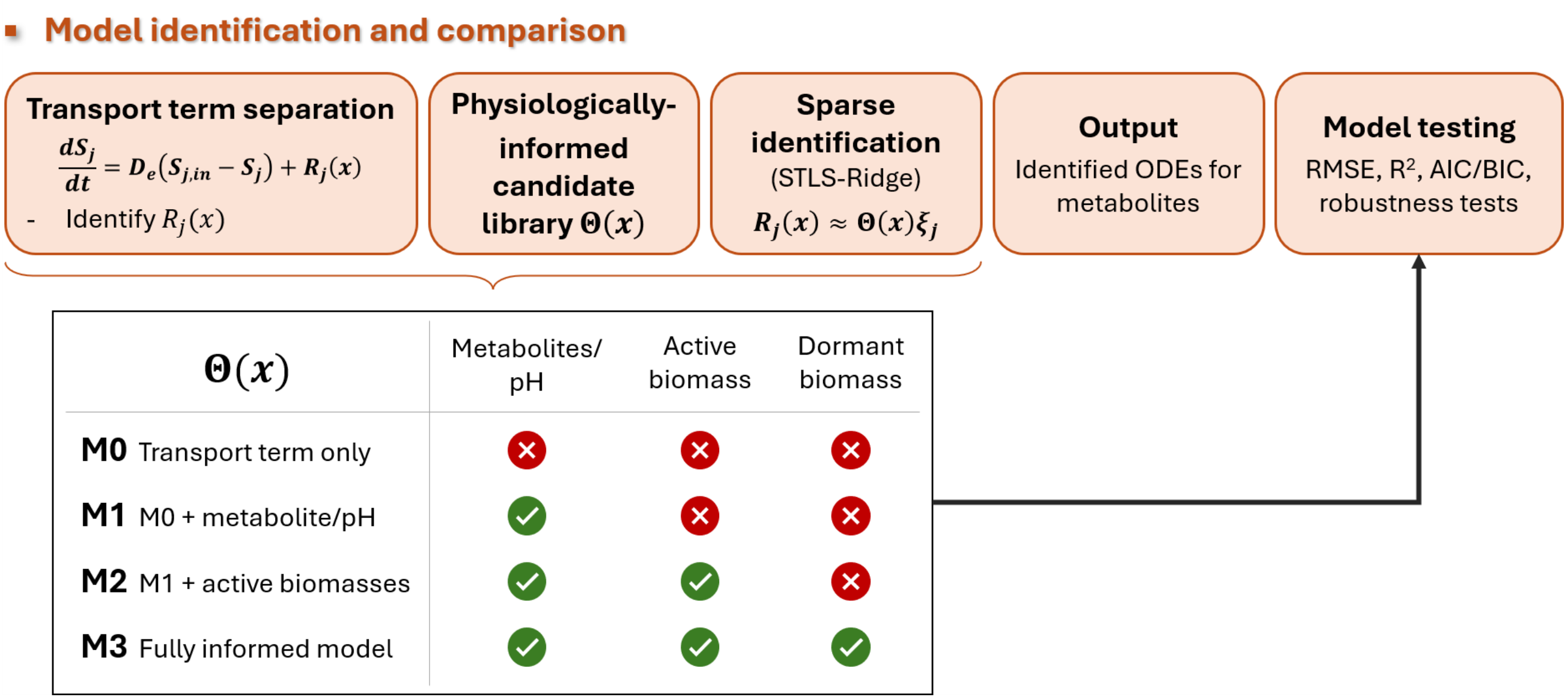

Figure 2: The comparison between the proposed SINDy-identified models, from M0 to M3.

Reply to Reviewer 2

A detailed reply to the reviewer's comments is given below:

Major Concerns

1. **Identifiability of active vs. dormant cell states The manuscript introduces dormant biomass states for both species and reconstructs their dynamics using a structured kinetic model, despite the fact that dormant populations are not directly measured. I would like to ask the authors to clarify, first, whether any experimentally measured data provide independent information that makes the active–dormant partition identifiable. Specifically:**

   (a) *The manuscript notes that rRNA FISH detects ribosome rich, translationally active cells rather than the entire community, while dormant populations are "experimentally inaccessible".*

   - Is rRNA FISH used in a quantitatively calibrated way to provide absolute active biomass (e.g., dry weight or cell carbon equivalents), or is it used only as a relative activity proxy?

   - If the latter, it is unclear how the scaling between fluorescence and biomass is fixed, and whether this introduces additional degrees of freedom.

   We thank the reviewer for the comment. The rRNA-FISH/flow cytometry measurements were used as active cell population information, not as independent dry cell weight measurements. The absolute biomass scale was fixed using OD_{600} measurements converted to biomass-equivalent concentration using OD-to-biomass conversion factor (Refer to our response to Comment 7 from Reviewer 3). The active cell information was then used to constrain the active fractions of C_{ac} and C_{lj} on this OD_{600}-derived biomass-equivalent scale. Therefore, $X_{C_{ac}}$ and $X_{C_{lj}}$ represent experimentally constrained active biomass-equivalent concentrations, while $X_{C^*_{ac}}$ and $X_{C^*_{lj}}$ are reconstructed dormant biomass-equivalent latent states on the same scale.

   (b) *From a systems modeling and identification perspective, the model appears to admit a scaling invariance: active and dormant biomasses enter the observable equations only through products with kinetic parameters (e.g., growth associated or maintenance like terms). As a result, different scalings of active vs. dormant biomass, combined with compensating kinetic rates, could yield identical metabolite concentration trajectories. If this interpretation is incorrect, it would be helpful for the authors to explain explicitly which measurements or constraints break this invariance (and therefore the non-identifiability of parameters).*

We thank the reviewer for raising this systems-identification point. The scaling invariance described by the reviewer would arise if the active/dormant biomass trajectories and the kinetic coefficients were estimated simultaneously from metabolite concentration trajectories alone. In the present workflow, however, the biomass-state trajectories are not freely rescaled during the SINDy regression.

The population states are first reconstructed on an OD_{600}-derived biomass-equivalent scale, with the total biomass constrained by OD_{600} measurements and the active populations constrained by rRNA-FISH/flow cytometry-derived active cell information. The active and dormant compartments are also constrained by the structured population-balance equations, including non-negativity and reversible active-dormant transition dynamics. After this reconstruction step, the active and dormant biomass trajectories are treated as fixed regressors in the SINDy identification of extracellular metabolic dynamics.

Therefore, the SINDy coefficients are identified conditional on the fixed biomass-equivalent population trajectories, not through an unconstrained joint rescaling of biomass states and kinetic coefficients.

2. **As a follow up point, even if the authors argue that the active–dormant decomposition is biologically plausible, the current formulation raises a model selection question. Given the available observables, a simpler model without explicit dormant states, in which all biomass is treated as active with effective kinetics, may fit and explain all the experimental data equally well. By standard parsimony and model selection criteria, such a model would be preferable unless the more complex formulation demonstrably improves identifiability or predictive performance. Currently, there is no such comparison provided in the results. I encourage the authors to:**

   (a) *Compare the proposed model against simpler alternatives (e.g., models without dormant states but retaining the SINDy learned metabolite dynamics)*

   We thank the reviewer for raising this important model-selection concern. In the revised manuscript, we have added a stepwise model comparison to evaluate whether the active-dormant formulation improves the identification of metabolite dynamics compared with simpler alternatives.

   We compared four models with increasing levels of physiological information. M0 included only the first-principles transport term. M1 used a SINDy library constructed from extracellular metabolite and pH variables only. M2 additionally included active-biomass terms but excluded all dormant-state terms. M3 corresponded to the full physiologically informed library, including active, dormant, and stress-

related terms. For each model, the SINDy regression was performed independently using the corresponding candidate library, with the same preprocessing, smoothing, and STLS-Ridge settings.

This comparison showed a clear improvement as physiological information was added to the library. M1 model gave a mean RMSE of 20.82 and a mean R^2 of 0.417. Including active-biomass terms (M2) reduced the mean RMSE to 6.77 and increased the mean R^2 to 0.817. The full physiologically informed model, M3, further reduced the mean RMSE to 5.30 and increased the mean R^2 to 0.873. These results show that the proposed full library improves the representation of extracellular metabolite dynamics compared with reduced alternatives, including the active-biomass-only model that excludes dormant-state terms. This result is also discussed in Reviewer 1 Comment 5, and the corresponding revision can be found in Section 1.5 of the SI.

(b) *Evaluate these alternatives using formal criteria such as AIC/BIC or cross validated prediction error. This comparison would clarify whether the introduction of dormant states is truly supported by the data or functions primarily as an internal explanatory construct.*

We have also evaluated these models using AIC and BIC to account for model complexity. For each metabolite equation, AIC and BIC were computed from the residual sum of squares (RSS) using the number of retained nonzero SINDy terms (k) as the complexity parameter:

$$\mathrm{AIC} = n \ln\left(\frac{\mathrm{RSS}}{n}\right) + 2k$$

$$\mathrm{BIC} = n \ln\left(\frac{\mathrm{RSS}}{n}\right) + k \ln(n),$$

where n is the number of time points and $RSS = MSE \times n$. Please note that the fixed first-principles transport terms were not counted in k, as these terms were specified from the perfusion mass balance and were not estimated by SINDy.

The full M3 library achieved the lowest total AIC and BIC values among the tested models in our additional investigation. The total AIC values were 23216.39, 15530.58, 10753.72, and 9582.11 for M0, M1, M2, and M3, respectively. The corresponding total BIC values were 23216.39, 15590.48, 10856.41, and 9714.75. Thus, even after penalizing the additional retained terms, M3 provided the best overall balance between model fit and complexity.

The improvement from M2 to M3 was metabolite-dependent, which we now state explicitly in the revised manuscript. This result supports the use of dormant- and stress-related terms in the full library at the overall model level, while avoiding the

stronger claim that such terms are equally necessary for every metabolite equation. The summarized table for such metrics have been inserted in Section 1.5 of the SI.

3. **The manuscript states that the full dataset was used for model identification and SINDy regression, without any splitting for validation. While this maximizes data usage, it makes it difficult to assess model generalizability, especially given the presence of unobserved latent states, the large number of kinetic parameters, and the high collinearity typical of fermentation time series data (for which the authors rightfully acknowledge and address by SINDy). It would strengthen the work to include for example, a temporal hold out (e.g., training on early/mid fermentation, testing on later phases), sensitivity analysis of the SINDy structure to perturbations in the reconstructed latent states, and a discussion of how overfitting risk was evaluated. Without such analysis, it is unclear whether the identified kinetic structures are robust features of the system or artifacts of fitting a flexible model to a single dataset.**

   We thank the reviewer for this important comment. It is crucial to evaluate generalizability for a flexible identification framework involving latent reconstructed states and correlated fermentation time-series data. In the revised manuscript and SI, we have therefore expanded the model-assessment section to clarify how overfitting risk and structural robustness were evaluated.

   First, we added several perturbation- and complexity-based analyses using the parimary manuscript dataset. These include noise-injection analysis (See Reviewer 1 Comment 1), missing-data subset analysis (See Reviewer 1 Comment 2), reduced-library comparison from M0 to M3 (See Reviewer 1 Comment 5), AIC/BIC evaluation (See Reviewer 1 Comment 6), λ-sensitivity analysis (See Reviewer 1 Comment 9 and 10), and leave-one-term-out analysis (See Reviewer 1 Comment 11) for the acetone and isopropanol equation. Together, these analyses examine whether the identified structures are sensitive to measurement noise, individual time points, threshold selection, or unnecessary model complexity.

   Second, as also discussed in our response to Reviewer 1 Comment 8, we examined an additional perfusion dataset as a possible external assessment case. This dataset was obtained under a substantially different operating regime, including higher glucose loading, an early C_{ac}-dominant phase, a large ethanol spike, and operational discontinuities associated with process configuration changes. Therefore, we did not present it as a strict parameter-free validation of the original M3 coefficients. Instead, we used it as an additional operating-regime assessment by applying the same physiologically informed SINDy library and equation-specific masks with segment-wise reestimation after excluding discontinuous transitions.

This analysis showed that the proposed library structure could represent the local metabolite dynamics of the additional dataset, while the reestimated coefficients differed from those obtained from the primary manuscript dataset. These results suggest that the SINDy structure is useful across distinct operating regimes, but that the fitted coefficients should be interpreted as regime-specific effective kinetic closures rather than universal kinetic constants.

Accordingly, we have revised the manuscript to clarify the validation scope and to avoid overstating generalizability. The revised discussion now states that strict external validation of the original coefficients will require additional independent coculture runs collected under comparable or systematically varied operating conditions with complete raw measurements, including OD_{600}, pH, metabolite profiles, and population-state measurements.

4. **The manuscript states that code is available upon reasonable request. For a study centered on data driven discovery, depositing the code in a public repository would greatly enhance reproducibility.**

   We thank the reviewer for raising this point. To improve transparency and reproducibility, we have created a public GitHub repository containing the MATLAB scripts and processed modeling dataset required to reproduce the M3 SINDy metabolite model reported in this manuscript.

   The repository includes the processed datasheet, the main reproduction script, and the helper functions used for candidate-library construction, STLS-Ridge sparse identification, and trajectory reconstruction. The released workflow starts from the processed modeling dataset and reproduces the transport-term subtraction, physiologically informed feature-library construction, equation-specific structural masking, sparse SINDy identification, and metabolite trajectory reconstruction for the reported metabolite equations.
   The repository is publicly available at: `https://github.com/cmst9469/sindy_coculture`.
   Accordingly, we have also revised the Data Availability statement.

Minor Comments and Clarifications

1. Several plots label quantities in "(a.u.)". I could not find a clear definition in the figure captions or main text. Please clarify explicitly whether this denotes arbitrary units. What exactly is being normalized or scaled? Is the same scaling used across time and across species?

   We thank the reviewer for the comment. Here, 'a.u.' denotes arbitrary units on the common biomass-derived scale used for the population-state reconstruction. The biomass scale was obtained from OD_{600} measurements using an OD-to-biomass conversion factor, which is a standard approach for representing microbial biomass in DCW-related units.

We kept the figure axis in arbitrary units since Figs. 2-3 show reconstructed active, dormant, and total population-state profile rather than independently measured species-specific CFU or DCW measurements. The same scaling convention was applied across all time points, species, and active/dormant compartments. Thus, the plotted profiles are comparable in relative magnitude and temporal trend within the population-reconstruction analysis. For the revised Figure captions, please refer to our response to Comment 2 from Reviewer 3.

2. **Dormant cells are defined as metabolically active but non growing. It may help readers if the authors briefly clarify how this definition differs from other commonly used terms (e.g., resting cells, maintenance state), and whether alternative interpretations would lead to equivalent model formulations.**

   We thank the reviewer for this helpful suggestion. In this work, the term 'dormant' is used as a lumped modeling descriptor for a viable, non-growing physiological state that retains low-level metabolic activity but does not actively contribute to growth-associated sugar consumption. This definition overlaps conceptually with resting or maintenance-state cells, but it is not intended to distinguish among all possible non-growing physiological states, such as resting sporulating, or other low-activity phenotypes. Given the available observables, these subcategories cannot be separately resolved yet.

   Thus, the dormant compartment in our model should be interpreted as an effective latent state representing the non-growing but metabolically retained fraction of the population. Alternative terminology, such as 'resting' or 'maintenance-state' cells, would lead to an equivalent model formulation as long as the state is treated as reversible, non-growing, and capable of low-level metabolic contribution. We have clarified this interpretation in the revised manuscript as follows:

   "Note that 'dormant' here is used as an effective latent-state descriptor for viable, non-growing cells that retain low-level metabolic activity. This definition overlaps with resting or maintenance-state cells, and the present data do not allow these non-growing phenotypes to be resolved separately. Therefore, the dormant compartment should be interpreted as a lumped non-growing but metabolically retained state. Alternative terms such as 'resting' or 'maintenance-state' would lead to an equivalent formulation under the same assumptions of reversibility and low-level metabolic activity."

3. **Related to the first two major concerns, given the model complexity, a short discussion acknowledging potential structural or practical identifiability limitations (even qualitatively) would improve transparency and help guide future experimental work.**

We thank the reviewer for the comment. This point is addressed together with the identifiability discussion in Major Concern 1 and the model selection analysis in Major Concern 2.

In response to Major Concern 1, we clarified how the active-dormant reconstruction is constrained by OD_{600}-derived biomass scale, active cell information, and the structured population-balance model. In response to Major Concern 2, we added reduced-library comparisons and AIC/BIC analyses to evaluate the contribution of dormant- and stress-related terms relative to simpler alternatives.

To connect these points in the revised manuscript, the following statement has been added to the discussion of model interpretation and validation scope:

"In addition, the reduced-library and AIC/BIC analyses support the practical usefulness of dormant- and stress-related terms at the overall model level, while independent perturbation experiments and additional state-specific measurements would further strengthen the evaluation of identifiability and generalizability."

Reply to Reviewer 3

A detailed reply to the reviewer's comments is given below:

Major Concerns

1. **This framework has distinct terms for "death" and "inactivation" (e.g. entering metabolic dormancy), but this statement blurs the distinction. There is a key difference however, a "dead" cell can't reactivate, whereas a dormant one can. The framework outlined in equation sets (1) and (2) seem to treat a "dead" cell as being removed from the population (e.g. it is not added to the inactivated cell population, and cells die in both populations). The definition of "death" as losing metabolic activity triggered by metabolite toxicity is confusing here.**

   We thank the reviewer for pointing out this important distinction. In the revised manuscript, we have clarified the distinction between inactivation and death. Inactivation refers to the reversible transition from the active state to the dormant state, whereas death represents an irreversible loss from the viable modeled population. Therefore, dead cells are not transferred to the dormant population and cannot reactivate. Both active and dormant cells can be removed through the death term, while only dormant cells can return to the active state through the reactivation term. We have revised the text to clarify this distinction and removed the previous wording that described death simply as a loss of metabolic activity.

   The following sentence (after Eq. 8) has been removed: *"In this framework, 'death' represents a loss of metabolic activity predominantly triggered by metabolite toxicity."*

   Instead, the following description has been added at the beginning of the same paragraph: *"In this framework, cell death is distinguished from inactivation. Cell inactivation denotes the reversible transition from active cells to dormant cells, and reactivation denotes the reverse transition from dormant cells back to active cells. By contrast, cell death is treated as an irreversible sink from the viable population. Cells undergoing death are removed from the modeled active or dormant populations and are not eligible for reactivation. The death term therefore acts as a sink from both active and dormant populations in Eqs. 1-2."*

2. **The author summary states "important cellular states, such as whether they are actively growing or temporarily inactive, cannot be directly measured." Yet, Figure 3 show experimental total dormancy measurements. How is this thing that is unmeasurable experimentally determined?**

We thank the reviewer the pointing out this ambiguity. The dormant population was not directly measured. The experimentally accessible quantities were the OD_{600}-derived total biomass and the rRNA-FISH/flow cytometry-based active-cell proxy. Dormant biomass was therefore inferred as a latent state constrained by these measurements and the structured population-balance model. The species-specific dormant populations, $X^*_{C_{ac}}$ and $X^*_{C_{lj}}$, are model-reconstructed quantities rather than independently measured observables. We have revised the figure caption and text to make this distinction explicit.

Figure 2 caption: *"Fig 2. Experimentally constrained population information used for latent-state reconstruction. Total biomass was estimated from OD_{600}, whereas the active cell population was obtained from rRNA-FISH/flow cytometry measurements. Dormant biomass was not directly measured, but was inferred as a latent state in the structured population model."*

Figure 3 caption: *"Fig 3. Comparison between experimentally constrained active population data and model-reconstructed population states. A: Active biomass concentrations. B: Model-reconstructed dormant biomass concentrations."*

Additional explanation has been added before the description of Fig. 3 as follows:

"Here, the dormant biomass trajectories should be interpreted as model-reconstructed latent states. Experimentally, OD_{600} provided total biomass information, while rRNA-FISH/flow cytometry measurements provided active cell population information; the dormant fractions were inferred through the structured population model rather than measured directly."

3. **According to the methods (line 93), Cac should lack butyrate formation due to the knockout of hbd. Yet in equation set (5), there is a butyrate term for Cac. Furthermore, butyrate is treated similarly to other products in all equation sets, but is clearly not a possible product. It is also not listed as part of the media definition. Futher, butyrate is shown as an input to Cac in Figure 1, but an output. Finally, figure 4d shows both gain and loss in butyrate concentration, suggesting that butyrate must be produced somehow within the coculture (otherwise a gain is not possible).**

We thank the reviewer for pointing out this important inconsistency. We agree that, because the engineered C_{ac} strain contains the Δhbd deletion, butyrate should not be described as a de novo product of C_{ac}. In the revised manuscript, we have corrected the butyrate narrative throughout the text.

Butyrate is treated as an externally supplied acid/substrate introduced through the feed stream, not as a product formed by the engineered C_{ac} strain. The butyrate term in Eq. 5 represents the contribution of feed-supplied butyrate to stress-induced inactivation,

rather than butyrate production. Similarly, in the metabolite balance, changes in butyrate concentration arise from the balance between feed replenishment, dilution, and biological consumption/reassimilation.

Specifically, in Eq. 12, the butyrate transport term is $0.075(S_{buty,in} - S_{buty})$ with $S_{buty,in} =$ 30 mM. Hence, butyrate concentration can increase when feed replenishment exceed biological consumption, even without cellular butyrate production. Conversely, butyrate decreases when biological consumption/reassimilation exceed the feed contribution. We have revised the text, equations, and figure annotation to remove any implication that butyrate is produced by C_{ac}, and to clarify that the observed butyrate profile reflects feed-dilution-consumption dynamics.

4. **How are the coefficients for the data-driven components arrived at? Is this an optimization-driven approach (like minimizing sum of squared residuals), or some other method?**

We thank the reviewer for the important question. The coefficients of the data-driven components in Eqs. 11-16 were obtained using an optimization-driven sparse regression procedure, SINDy. Specifically, after subtracting the first-principles transport contribution from the metabolite mass balance, the remaining biological reaction rate was regressed against the physiologically informed candidate library using sequential threshold least squares with ridge regularization (STLS-Ridge). At each iteration, a ridge-regularized least-squared problem was solved, coefficients below the threshold value were removed, and the regression was repeated on the remaining active terms. Thus, the reported coefficients correspond to the sparse regression coefficients selected by the STLS-Ridge procedure, rather than manually assigned values. We have revised the Method section ('Structural constraints and robust identification' section, particularly) to make this coefficient estimation procedure more explicit, as indicated below:

"After the first-principles transport contribution was specified, the unknown biological reaction term was isolated from the metabolite mass balance (See Eq. 10). For each metabolite j, the regression target was defined as:

$$R_j = \frac{dS_j}{dt} - D_e(S_{j,\text{in}} - S_j)$$

such that R_j represents the net biological contribution to the extracellular metabolite dynamics. This term was then approximated as a sparse linear combination of physiologically informed candidate functions,

$$R_j \approx \Theta_j \xi_j$$

where Θ_j is the candidate library for metabolite j and ξ_j is the corresponding coefficient

vector. The coefficient vector was estimated using the STLS-Ridge procedure. Specifically, a ridge-regularized least-squares problem was solved iteratively as:

$$\hat{\xi}_j = \arg\min_{\xi_j} |R_j - \Theta_j \xi_j|_2^2 + \gamma |\xi_j|_2^2 .$$

After each regression step, candidate terms with coefficient magnitudes below the threshold λ were removed, using the remaining active terms until the selected active set no longer changed. The nonzero coefficients obtained from this procedure define the identified data-driven reaction terms, as shown later in Eqs. 11-16."

5. **There is no associated github or Zenodo repository for this paper, nor supplemental files. Anyone wishing to use this model would have to program it themselves from scratch and take the authors' parameterization as accurate because it cannot be checked against the data. This is surprising as the modeling is done in MATLAB, so these could easily be provided.**

   We thank the reviewer for pointing out the data availability. Regarding this, please kindly refer to the Major Comment 3 from Reviewer 2 for details. We have uploaded our source code and datasheet in the public GitHub repository available at: `https://github.com/cmst9469/sindy_coculture`. Accordingly, we have also revised the Data Availability statement.

Minor Concerns

6. **Reactivation is not listed here, though it is included as a key term in equation sets (1) and (2), and is also defined in line 182. Is there a reason for this?**

   We thank the reviewer for pointing out the missing component. There was no specific reason but an oversight in the text. Because reactivation is explicitly included in Eqs. 1-2 through the k_r terms and defined as the transition from the dormant state back to the active state, we have revised the sentence to include reactivation among the primary mechanisms governing the cell population dynamics. The added part is marked as bold text below:

   "The temporal evolution of C_{ac} and C_{lj} biomasses in the reactor is governed by five primary mechanisms: specific cell growth, inactivation (transition from active to dormant), ***reactivation (transition from dormant to active)****, cell death, and physical removal via bleeding."*

7. **Equation sets (1) and (2): Are these cell populations tied to a particular unit (e.g. CFUs) or to an experimentally measurable quantity (like OD600)?**

We thank the reviewer for this clarifying question. The cell population variables in Eqs. 1-2 are not expressed in CFUs. They are expressed as biomass-equivalent concentrations derived from OD_{600} measurements using an OD-to biomass conversion factor. The OD_{600}-derived total biomass provides the overall biomass scale, while the rRNA-FISH/flow cytometry measurements provide active-cell population information for each species. Accordingly, the active populations, $X_{C_{ac}}$ and $X_{C_{lj}}$, are experimentally constrained biomass-equivalent quantities. The dormant populations, $X_{C^*_{ac}}$ and $X_{C^*_{lj}}$, are reconstructed latent states expressed on the same biomass-equivalent scale rather than independently measured CFU counts or dry cell weight measurements. We have clarified the units and experimental basis of these population variables in the revised manuscript, after Eqs. 1-2, as follows:

"The population variables in Eqs. 1-2 are expressed as biomass-equivalent concentrations. The total biomass scale was obtained from OD_{600} measurements using an OD-to-biomass conversion factor of 0.50 gDCW L^{-1} OD_{600}^{-1}*, while rRNA-FISH/flow cytometry measurements were used to constrain the active populations of C_{ac} and C_{lj}. Thus, $X_{C_{ac}}$ and $X_{C_{lj}}$ represent experimentally constrained active biomass-equivalent concentrations, whereas $X_{C^*_{ac}}$ and $X_{C^*_{lj}}$ represent model-reconstructed dormant biomass-equivalent latent states on the same scale."*

8. **Equation set (3): similarly, units for the $P_{mix,C_{ac}}$ term are also unclear. Is this a sum of pH and acid concentrations? (Later note: this is addressed well after equation set 3 in lines 213 to 220, and perhaps needs to be put earlier in the text). Further, if the pH of the system is automatically controlled by addition of 4 M NaOH (line 111), how is pH control accounted for in the setup of the exponential inhibition term? Similar concerns apply to equation set (4).**

We thank the reviewer for this clarifying question. We agree that the definition of P_{mix} should be introduced earlier, immediately after the growth-rate equations.

P_{mix}, the product-inhibition term, is dimensionless. It is not a direct sum of pH and metabolite concentrations. Rather, P_{mix} is defined as the sum of normalized inhibition contributions:

$$P_{mix} = \sum_j \frac{S_{j,\text{eff}}}{K_{I,j}},$$

where $S_{j,\text{eff}}$ is the effective concentration of inhibitory metabolite j, and $K_{I,j}$ is the corresponding inhibition constant. Because both $S_{j,\text{eff}}$ and $K_{I,j}$ are expressed in mM, each term $S_{j,\text{eff}}/K_{I,j}$ becomes dimensionless, making P_{mix} dimensionless and the exponential inhibition term $\exp(-P_{mix})$ dimensionally consistent.

In addition, the pH is not added directly to P_{mix}. Instead, the measured pH trajectory is used to calculate the undissociated acid fractions of acetate and butyrate using the

Henderson-Hasselbalch relationship. Although the reactor pH was controlled by automated NaOH addition, the measured pH values were still used in the model to account for residual pH variation and its effect on weak-acid speciation. For neutral solvents, the effective concentration was taken to be equal to the measured total concentration.

We have moved this explanation closer to Eqs. 3-4 and clarified that the same definition applies to both $P_{mix,C_{ac}}$ and $P_{mix,C_{lj}}$.

9. **Equation set (5): How are the hill coefficients selected?**

We thank the reviewer for the comment. The Hill coefficient in Eq. 5 was fixed at $n = 2$ as a phenomenological choice to represent a threshold-like, saturating increase in stress-induced inactivation with increasing inhibitory metabolite concentration. The Hill coefficient was fixed rather than fitted so that the stress-response exponent remained a prescribed modeling choice, while the sensitivity coefficients (e.g., $k_{i,ace}$) and half-saturation constants (e.g., K_{IPA}) were estimated from the data. We have clarified this modeling assumption in the revised manuscript as follows, after Eq. 5:

"The Hill coefficient was fixed at $n = 2$ as a phenomenological choice to capture a threshold-like, saturating stress response while avoiding additional non-identifiability from fitting the exponent itself."

10. **Equation set (7): How does effective concentration differ from actual concentration?**

We thank the reviewer for the clarifying question. The actual concentration refers to the measured total extracellular concentration, whereas the effective concentration refers to the concentration used to represent inhibitory activity in the product-inhibition term. For neutral solvents, the effective concentration is identical to the measured total concentration. On the other hand, for the weak organic acids, including acetate and butyrate, only the undissociated acid fraction was used as the effective concentration because this fraction is primarily responsible for membrane permeation and intracellular acidification. This fraction was calculated from the measured total acid concentration and pH using the Henderson-Hasselbalch relationship in Eq. 6.

11. **Table 1: It can be assumed that these values come from existing experimental observations (published elsewhere) or from fitting the model parameters to experimental data. It is not clear which is done, but it is likely the former. However, this table lacks citations.**

We thank the reviewer for pointing this out. The numerical values listed in Table 1 were not directly adopted from previously reported parameter set. Rather, they were estimated

in this study by fitting the structured population model to the experimental data. To make the source of these parameter values explicit, we have revised the text and the caption of Table 1 to explicitly state that the listed values are fitted model parameters estimated from the experimental dataset used in this study. The caption is modified as follows:

"Table 1. Kinetic parameters and inhibition constants used in the co-culture model to fit the structured population model to the experimental dataset used in this study."

12. **Equation sets (11) to (16): How is the coefficient of -0.075 arrived at from "first principles"? Is this a dilution rate? Characteristic time?**

We thank the reviewer for pointing this out. The coefficient, -0.075, is the effective dilution rate calculated from the perfusion mass balance, not a fitted parameter. Specifically, using $F \approx 0.30$ L h^{-1} and the total working volume $V = 3.6$ L gives

$$D = \frac{F}{V} \approx 0.083 \text{ h}^{-1}.$$

As a 10% bleed was applied, the effective dilution rate for extracellular metabolites was calculated as

$$D_e = 0.9D \approx 0.075 \text{ h}^{-1}.$$

Therefore, for metabolites not present in the feed, the first-principles transport term $D_e(S_{j,\text{in}} - S_j)$ reduced to $-0.075S_j$. To clarify this in the revised manuscript, a brief explanation has been added after Eq. 11, where the number first appears, as follows:

"The coefficient 0.075 h^{-1} *in the first-principles transport term represents the effective dilution rate under perfusion operation. Based on the feed flow rate* $F \approx 0.30$ L h^{-1} *and the total working volume* $V = 3.6$ L*, the nominal dilution rate is* $D = F/V \approx 0.083$ h^{-1}*. After accounting for the 10% bleed fraction, the effective dilution rate for extracellular metabolites becomes* $D_e = 0.9D \approx 0.075$ h^{-1}*. Thus, when a metabolite is absent from the feed stream,* $D_e(S_{j,\text{in}} - S_j)$ *simplifies to* $-0.075S_j$*."*
