# Supplementary material for: From observable fermentation data to hidden cell states: A modeling study of a mixotrophic Clostridium coculture under perfusion mode
Source: PLoS Comput Biol. 2026 Sep 28;22(9):e1014759. doi: 10.1371/journal.pcbi.1014759 (PMC13619069; doi:10.1371/journal.pcbi.1014759)
Supplement: Supplementary file 3 [file pcbi.1014759.s004.pdf]

List of changes

The items that have been changed are:

1. Line 158-166 of the Main Text: With an additional description, we clarified how the rRNA-FISH/flow cytometry measurements were processed and used in the model.
2. We revised Section 1.5 of the SI to clarify the reduced SINDy model hierarchy from M0 to M3. In particular, we clarified that M0 has a constant offset rather than setting $\Theta(\mathbf{x}) = 0$.
3. We added a new feature-inclusion table into Section 1.5 of the SI to specify which candidate-feature groups were included in each reduced SINDy model.
4. We added a practical interpretation of the M2 model in Section 1.5 of the SI, clarifying that the present M2 model should not be understood as an OD_{600}-only model but it still uses rRNA-FISH/flow cytometry-constrained active-biomass trajectories.

Reply to Reviewer 2

1. **There is a missing link from the rRNA-FISH + flow cytometry described in the Methods section. The output from flow cytometry is probably a distribution of fluorescence intensity. How exactly was the data used in the model? Was fluorescence intensity data processed to get a percentage of metabolically active cells in each sample, followed by multiplying by the total biomass unit converted from OD 600 to estimate XCac and XClj? Please clarify how exactly the experimental data was used to estimate/fit variables/parameters.**

   We appreciate the reviewer's comment. We agree that the connection between the rRNA-FISH/flow cytometry measurements and the model variables should be described more explicitly. Unlabeled (metabolically inactive) cells and labeled cells appear as two discrete groups from flow cytometry as shown in Figure 1 [1]. Fraction of the labeled cells was quantified by applying thresholds that were determined based on negative and positive control experiments. To clarify the data processing, the following description has been added to the 'Flow cytometry' subsection (Page 5):

   "For population-fraction estimation, fluorescence thresholds for rRNA-FISH-labeled cells were established based on the distribution observed in negative control experiments. Cells exhibiting fluorescence signals above the corresponding threshold were classified as active cells for each species. The resulting active fractions of C_{ac} and C_{lj} were multiplied by the

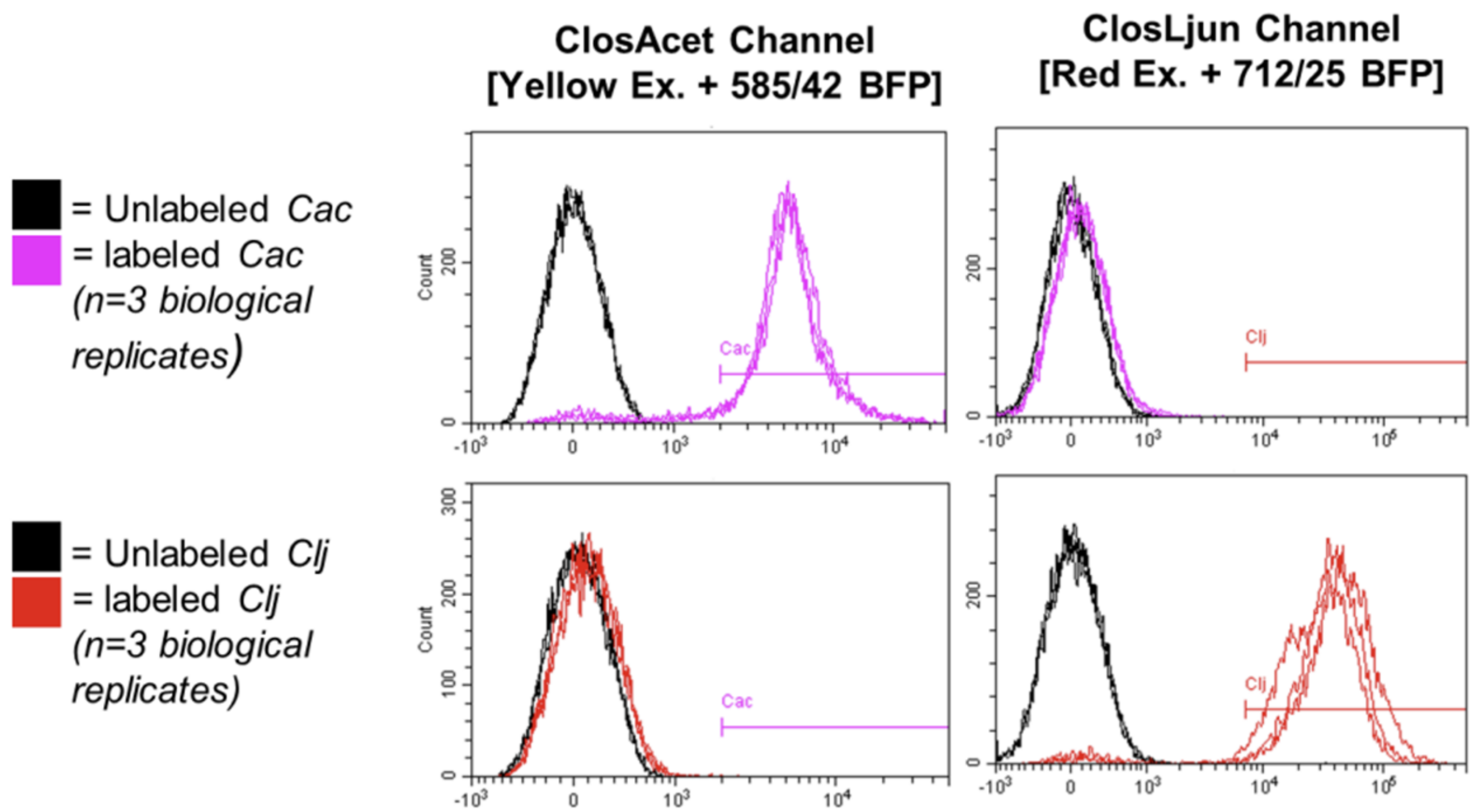

Figure 1: **Representative flow cytometry histograms used to distinguish rRNA-FISH-labeled active cells from unlabeled cells in C_{ac} and C_{lj} populations [1].**

OD_{600}-derived total biomass-equivalent concentration to estimate $X_{C_{ac}}$ and $X_{C_{lj}}$, respectively. Unlabeled cells were not treated as independently measurable values, and $X_{C^*_{ac}}$ and $X_{C^*_{lj}}$ were reconstructed as latent states in the structured population model as discussed below."

2. **The model complexity vs. performance comparison is an excellent addition. Models M0 – M3 probably refer to what features in Table 2 are available for SINDy but they are not exactly clear from the new supplementary information file (section 1.5 and Figure E). For example, does M0 mean theta(x) = 0 or a constant? Does M1 include pH-dependent inhibition term because of acid dissociation? Does M2 include additionally active biomass only or also biomass-coupled growth terms which also include active biomass so the growth-coupled terms are not in M0 and M1? Maybe put an extended form of Table 2 in the SI file with a new column specifying which models tested include the feature.**

   We thank the reviewer for kind suggestion. According to your comment, we added materials in the SI to clarify differences among reduced models, particularly in relation to the candidate features listed in Table 2.

   First, we have added the following statement right after the bullet points so that we clarified that M0 does not correspond to $\Theta(\mathbf{x}) = 0$ but represents a transport-plus-constant baseline:

   "Note that M0 does not correspond to $\Theta(\mathbf{x}) = 0$. In M0, the unknown biological reaction term R_j is approximated by a constant offset."

   Subsequently, the extended form of Table 2 (which will be Supporting Table D in the SI) has been added with the following description:

   "The overall comparison procedure is also illustrated in Fig. E, and Table D summarizes which candidate-feature groups were included in the SINDy library for M0–M3. The fixed first-principles transport term, $D_e\,(S_{j,in} - S_j)$, was applied to all models before sparse regression and was not counted as a fitted SINDy candidate."

   This added table and description now clarify how the reduced model hierarchy was constructed and how each model differs in terms of the candidate SINDy library.

3. **An interesting observation is that the M2 active-biomass-informed model already drastically improved the SINDy results while the M3 full model further significantly improved the SINDy results. So for practical modeling point of view, if rRNA-FISH/flow cytometry data is not available (which is probably the case in many other studies), would the M2 form of model with SINDy still be applicable and get reasonably good results, say, only with biomass estimated**

Table D: **Candidate-feature groups included in the reduced SINDy models M0–M3.**

**Feature group**	**Representative terms**	**M0**	**M1**	**M2**	**M3**
Constant offset in $R_j(\mathbf{x})$	1	✓	✓	✓	✓
Extracellular metabolites	S_{glu}, S_{fru}, S_{ace}, S_{acn}, S_{EtOH}, S_{IPA}, S_{BuOH}, S_{buty}	×	✓	✓	✓
pH-derived acidity term	$[H^+] = 10^{-pH+6}$	×	✓	✓	✓
Active biomass terms	$X_{C_{ac}}$, $X_{C_{lj}}$	×	×	✓	✓
Biomass-coupled active growth terms	$\mu_{C_{ac}} X_{C_{ac}}$, $\mu_{C_{lj}} X_{C_{lj}}$	×	×	✓	✓
Dormant biomass terms	$X_{C^*_{ac}}$, $X_{C^*_{lj}}$	×	×	×	✓
Stress-modulated metabolic-shift terms	$S_j X/(1 + P_{mix})$, $S_j X P_{mix}/(1 + P_{mix})$	×	×	×	✓

✓: included / ×: excluded

from OD600? Or the active vs. dormant population dynamics, which I think was kept in all these tested models here, is pretty much still largely needed for the good performance of the M2 model? This is probably something that people who want to model their fermentation data are interested to know.

We appreciate the reviewer's insightful comment. In the revised SI (Section 1.5), we added a discussion clarifying that the reduced-model comparison was designed to evaluate the candidate-feature sets used in the SINDy library for the extracellular metabolite equations, while the population-state reconstruction step was kept the same.

Specifically, M2 excludes dormant-biomass and stress-modulated terms from the SINDy candidate library, but it still uses active biomass trajectories, $X_{C_{ac}}$ and $X_{C_{lj}}$, derived from rRNA-FISH/flow cytometry-constrained population estimates. Therefore, the present M2 result should not be interpreted as demonstrating an OD_{600}-only modeling strategy. If only total OD_{600} measurements are available, an M2 type model would require an additional approximation of active biomass fractions from total biomass, growth-phase information, or another population-state estimation method. Without such active biomass information, the available input information would be closer to the M1 metabolite/pH-informed case.

We also clarified that the improvement from M1 to M2 supports the practical value of active biomass information, whereas the further improvement from M2 to M3 supports the additional contribution of dormant biomass and stress-related terms.

Regarding this, we have added the following sentences:

"From a practical modeling perspective, the M2 model suggests that an active-biomass-informed SINDy library can provide a useful reduced alternative when direct dormant-state information is not included in the candidate library. However, M2 should not be interpreted

as an OD_{600}-only model in the present analysis. The active biomass trajectories, $X_{C_{ac}}$ and $X_{C_{lj}}$, were still obtained using the rRNA-FISH/flow cytometry-constrained population estimates. Thus, when rRNA-FISH/flow cytometry data are unavailable, applying an M2-type library would require an additional approximation of the active biomass fractions from total OD_{600}, growth-phase information, or another population-state estimation method. Without such an approximation, the model would be closer to the M1 metabolite/pH-informed case than to the M2 active-biomass-informed case."

References

[1] Hill JD, Papoutsakis ET. Species-specific ribosomal RNA-FISH identifies interspecies cellular-material exchange, active-cell population dynamics and cellular localization of translation machinery in clostridial cultures and co-cultures. mSystems. 2024;9(10):e00572-24. http://dx.doi.org/10.1128/msystems.00572-24 doi:10.1128/msystems.00572-24.
