# Supplementary material for: From observable fermentation data to hidden cell states: A modeling study of a mixotrophic Clostridium coculture under perfusion mode
Source: PLoS Comput Biol. 2026 Sep 28;22(9):e1014759. doi: 10.1371/journal.pcbi.1014759 (PMC13619069; doi:10.1371/journal.pcbi.1014759)
Supplement: Supplementary file 4 [file pcbi.1014759.s002.docx]

**Title:** From observable fermentation data to hidden cell states: A modeling study of a mixotrophic Clostridium coculture under perfusion mode

**Authors:** Juhyeon Kim et al.

**Manuscript ID:** PCOMPBIOL-D-26-00654

**Recommendation:**

**Summary:**

A Clostridium coculture can of an ABE producer (*Clostridium acetobutylicum*) and CO_2_ fixer (*Clostridium ljundalhii*) could improve total ABE yield over just an ABE producer. In culture, each presents four possible states (active growing, resting, sporulating, and spore). Existing kinetic (or stoichiometric) models and ML approaches lack the resolution or interpretability (respectively) for identifying abundances of sub populations. A new framework based on Sparse Identification of Nonlinear Dynamics (SINDy) is used to try to account for two states: active and dormant to more accurately model population and metabolic dynamics. The framework is based largely on Monod- and Hill- type kinetic equations, simple population balances (allows for cell death and movement between populations), and metabolite mass balances.

**Summary of concerns:**

Mostly, the manuscript is well-written and addresses the important issue of heterogeneity in cell states in a mixed coculture. The main issue with this manuscript is obscuring some key steps and data sources. Once this is resolved, it should be appropriate for publication in this journal.

**Major Concerns:**

1. **Lines 243 to 246:** This framework has distinct terms for “death” and “inactivation” (e.g. entering metabolic dormancy), but this statement blurs the distinction. There is a key difference however, a “dead” cell can’t reactivate, whereas a dormant one can. The framework outlined in equation sets (1) and (2) seem to treat a “dead” cell as being removed from the population (e.g. it is not added to the inactivated cell population, and cells die in both populations). The definition of “death” as losing metabolic activity triggered by metabolite toxicity is confusing here.
2. **Figure 3:** The author summary states “important cellular states, such as whether they are actively growing or temporarily inactive, cannot be directly measured.” Yet, Figure 3 show experimental total dormancy measurements. How is this thing that is unmeasurable experimentally determined?
3. **Butyrate treatment throughout the manuscript:** According to the methods (line 93), C_ac_ should lack butyrate formation due to the knockout of hbd. Yet in equation set (5), there is a butyrate term for C_ac_. Furthermore, butyrate is treated similarly to other products in all equation sets, but is clearly not a possible product. It is also not listed as part of the media definition. Futher, butyrate is shown as an input to Cac in Figure 1, but an output. Finally, figure 4d shows both gain and loss in butyrate concentration, suggesting that butyrate must be produced somehow within the coculture (otherwise a gain is not possible).
4. **Data-driven components coefficients:** How are the coefficients for the data-driven components arrived at? Is this an optimization-driven approach (like minimizing sum of squared residuals), or some other method?
5. **Data and model availability.** There is no associated github or Zonodo repository for this paper, nor supplemental files. Anyone wishing to use this model would have to program it themselves from scratch and take the authors’ parameterization as accurate because it cannot be checked against the data. This is surprising as the modeling is done in MATLAB, so these could easily be provided.

**Minor Concerns:**

6. **Lines 176 to 178:** reactivation is not listed here, though it is included as a key term in equation sets (1) and (2), and is also defined in line 182. Is there a reason for this?
7. **Equation sets (1) and (2):** Are these cell populations tied to a particular unit (e.g. CFUs) or to an experimentally measurable quantity (like OD600)?
8. **Equation set (3):** similarly, units for the $P_{mix,C_{ac}}$ term are also unclear. Is this a sum of pH and acid concentrations? (Later note: this is addressed well after equation set 3 in lines 213 to 220, and perhaps needs to be put earlier in the text). Further, if the pH of the system is automatically controlled by addition of 4 M NaOH (line 111), how is pH control accounted for in the setup of the exponential inhibition term? Similar concerns apply to equation set (4).
9. **Equation set (5):** How are the hill coefficients selected?
10. **Equation set (7):** How does effective concentration differ from actual concentration?
11. **Table 1:** It can be assumed that these values come from existing experimental observations (published elsewhere) or from fitting the model parameters to experimental data. It is not clear which is done, but it is likely the former. However, this table lacks citations.
12. **Equation sets (11) to (16):** How is the coefficient of -0.075 arrived at from “first principles”? Is this a dilution rate? Characteristic time?

**Concerns of spelling, grammar, and clarity:**

1. The manuscript is well-written and generally clear.
