# Supplementary material for: From observable fermentation data to hidden cell states: A modeling study of a mixotrophic Clostridium coculture under perfusion mode
Source: PLoS Comput Biol. 2026 Sep 28;22(9):e1014759. doi: 10.1371/journal.pcbi.1014759 (PMC13619069; doi:10.1371/journal.pcbi.1014759)
Supplement: Peer review history

# Peer review: Decision Letter 0

**DOI:** 10.1371/journal.pcbi.1014759.r001

**Authors:** Marc R Birtwistle, Rajib Saha

5 May 2026

From observable fermentation data to hidden cell states: A modeling study of a mixotrophic *Clostridium*  coculture under perfusion mode

PLOS Computational Biology

Dear Dr. Kwon,

Thank you for submitting your manuscript to PLOS Computational Biology. After careful consideration, we feel that it has merit but does not fully meet PLOS Computational Biology's publication criteria as it currently stands. Therefore, we invite you to submit a revised version of the manuscript that addresses the points raised during the review process.

Please submit your revised manuscript by Jul 05 2026 11:59PM. If you will need more time than this to complete your revisions, please reply to this message or contact the journal office at ploscompbiol@plos.org. When you're ready to submit your revision, log on to https://www.editorialmanager.com/pcompbiol/ and select the 'Submissions Needing Revision' folder to locate your manuscript file.

Please include the following items when submitting your revised manuscript:

* A letter that responds to each point raised by the editor and reviewer(s). You should upload this letter as a separate file labeled 'Response to Reviewers'. This file does not need to include responses to formatting updates and technical items listed in the 'Journal Requirements' section below.

* A marked-up copy of your manuscript that highlights changes made to the original version. You should upload this as a separate file labeled 'Revised Manuscript with Track Changes'.

* An unmarked version of your revised paper without tracked changes. You should upload this as a separate file labeled 'Manuscript'.

If you would like to make changes to your financial disclosure, competing interests statement, or data availability statement, please make these updates within the submission form at the time of resubmission. Guidelines for resubmitting your figure files are available below the reviewer comments at the end of this letter

We look forward to receiving your revised manuscript.

Kind regards,

Rajib Saha, Ph.D.

Academic Editor

PLOS Computational Biology

Marc Birtwistle

Section Editor

PLOS Computational Biology

**Journal Requirements:**

**Reviewers' comments:**

Reviewer's Responses to Questions

**Comments to the Authors:**

**Please note that one review is uploaded as an attachment.**

Reviewer #1: This article presents a mathematical model for predicting coculture dynamics within bioreactors. Given the increasing industrial relevance of synthetic microbial consortia, modeling the metabolic interactions of diverse cell subpopulations is a timely and important challenge. While the topic is significant, the statistical framework is currently preliminary and more analysis is required to show generalizability and robustness.

Major Revisions

1. The current analysis lacks a thorough investigation into data sensitivity. The authors should perform noise-injection experiments and sensitivity analyses to determine how measurement noise impacts model predictions.

2. Address Missing Data: Study the effect of missing values and outliers by sampling subsets of the data.

3. Evaluate Parameter Sensitivity: Provide a sensitivity analysis for the parameters in Table 1 (e.g., how a 10% variation impacts the fit, feature selection, or error rates).

4. Replicate Analysis: Clarify whether the experimental data used are biological or technical replicates, and how many were used. The model should be evaluated using individual replicates rather than just averaged data to account for experimental variance.

5. Benchmark Against Baseline and Other models: Compare the proposed model with purely data-driven methods and hybrid models.

6. Complexity Penalization: Use information criteria such as AIC or BIC to justify model complexity and evaluate stepwise models.

7. Report standard performance metrics, including MSE, R2, and AUC, particularly for hidden parameters such as dormant vs. active population ratios.

8. The most significant weakness is that the model is currently trained and assessed on the same dataset. The performance must be assessed on independent datasets or runs that include novel variations (e.g., differences in growth rates or initial compositions).

9. Hyperparameters (such as lambda) should be determined using a dedicated training set.

10. Demonstrate model stability by showing whether the selected features or equations remain consistent when hyperparameters are varied.

11. The authors should justify the inclusion of each variable, particularly in equations 15 and 16, in terms of improvement in fit. Provide a series of fits with fewer variables to show the marginal gain of each additional parameter. If removing the least important variable reduces performance by only 1%, a simpler, more parsimonious model may be preferable for generalization.

12. The "regulatory logic" described in the paper lacks prospective or causal validation. It is unclear if the identified relationships are causal or merely correlative. The authors should perform temporal or perturbation analyses to demonstrate that these learned relationships reflect actual regulatory mechanisms.

13. The abstract needs more specificity regarding exactly what is being predicted. The results are currently described at a superficial level.

14. Given that the model has not been rigorously evaluated for generalizability, the claims made throughout the paper should be significantly toned down. This applies to both the abstract and the conclusion; the latter should focus more on current limitations than broad potential.

15. Figures should include simple schematics explaining the models, illustrating the relationship between input data, the governing equations, and the resulting outputs. Visual comparisons with simpler control models should also be included.

Reviewer #2: This manuscript presents a hybrid modeling framework that combines a structured kinetic population model with SINDy to infer governing dynamics in a Clostridium coculture. The attempt to integrate mechanistic insight with data driven discovery is timely and potentially impactful. Overall, this is an interesting and well written manuscript.

At the same time, the central modeling choices would benefit from clearer justification and stronger model selection evidence. Specifically, I have some major concerns regarding (i) the identifiability of some model parameters, (ii) model selection justification of the active/dormant biomass decomposition, and (iii) the absence of any form of cross validation or out of sample assessment, among other comments. Addressing these issues would substantially strengthen the rigor and interpretability of the study.

Major Concern 1: Identifiability of active vs. dormant cell states

The manuscript introduces dormant biomass states for both species and reconstructs their dynamics using a structured kinetic model, despite the fact that dormant populations are not directly measured. I would like to ask the authors to clarify, first, whether any experimentally measured data provide independent information that makes the active–dormant partition identifiable. Specifically:

1. The manuscript notes that rRNA FISH detects ribosome rich, translationally active cells rather than the entire community, while dormant populations are “experimentally inaccessible”.

- Is rRNA FISH used in a quantitatively calibrated way to provide absolute active biomass (e.g., dry weight or cell carbon equivalents), or is it used only as a relative activity proxy?

- If the latter, it is unclear how the scaling between fluorescence and biomass is fixed, and whether this introduces additional degrees of freedom.

2. From a systems modeling and identification perspective, the model appears to admit a scaling invariance: active and dormant biomasses enter the observable equations only through products with kinetic parameters (e.g., growth associated or maintenance like terms). As a result, different scalings of active vs. dormant biomass, combined with compensating kinetic rates, could yield identical metabolite concentration trajectories. If this interpretation is incorrect, it would be helpful for the authors to explain explicitly which measurements or constraints break this invariance (and therefore the non-identifiability of parameters).

Major Concern 2: Model selection

As a follow up point, even if the authors argue that the active–dormant decomposition is biologically plausible, the current formulation raises a model selection question. Given the available observables, a simpler model without explicit dormant states, in which all biomass is treated as active with effective kinetics, may fit and explain all the experimental data equally well. By standard parsimony and model selection criteria, such a model would be preferable unless the more complex formulation demonstrably improves identifiability or predictive performance. Currently, there is no such comparison provided in the results.

I encourage the authors to (1) compare the proposed model against simpler alternatives (e.g., models without dormant states but retaining the SINDy learned metabolite dynamics); and (2) evaluate these alternatives using formal criteria such as AIC/BIC or cross validated prediction error. This comparison would clarify whether the introduction of dormant states is truly supported by the data or functions primarily as an internal explanatory construct.

Major Concern 3: Lack of cross validation or out of sample assessment

The manuscript states that the full dataset was used for model identification and SINDy regression, without any splitting for validation. While this maximizes data usage, it makes it difficult to assess model generalizability, especially given the presence of unobserved latent states, the large number of kinetic parameters, and the high collinearity typical of fermentation time series data (for which the authors rightfully acknowledge and address by SINDy).

It would strengthen the work to include for example, a temporal hold out (e.g., training on early/mid fermentation, testing on later phases), sensitivity analysis of the SINDy structure to perturbations in the reconstructed latent states, and a discussion of how overfitting risk was evaluated.

Without such analysis, it is unclear whether the identified kinetic structures are robust features of the system or artifacts of fitting a flexible model to a single dataset.

Major Concern 4: Code and data availability

The manuscript states that code is available upon reasonable request. For a study centered on data driven discovery, depositing the code in a public repository would greatly enhance reproducibility.

Minor Comments and Clarifications

1. Use of “(a.u.)” in figures

Several plots label quantities in “(a.u.)”. I could not find a clear definition in the figure captions or main text. Please clarify explicitly whether this denotes arbitrary units. What exactly is being normalized or scaled? Is the same scaling used across time and across species?

2. Terminology: dormancy vs. non growing active cells

Dormant cells are defined as metabolically active but non growing. It may help readers if the authors briefly clarify how this definition differs from other commonly used terms (e.g., resting cells, maintenance state), and whether alternative interpretations would lead to equivalent model formulations.

3. Parameter identifiability discussion

Related to the first two major concerns, given the model complexity, a short discussion acknowledging potential structural or practical identifiability limitations (even qualitatively) would improve transparency and help guide future experimental work.

Reviewer #3: Review is included as an attachment.

**********

**Have the authors made all data and (if applicable) computational code underlying the findings in their manuscript fully available?**

The PLOS Data policy requires authors to make all data and code underlying the findings described in their manuscript fully available without restriction, with rare exception (please refer to the Data Availability Statement in the manuscript PDF file). The data and code should be provided as part of the manuscript or its supporting information, or deposited to a public repository. For example, in addition to summary statistics, the data points behind means, medians and variance measures should be available. If there are restrictions on publicly sharing data or code —e.g. participant privacy or use of data from a third party—those must be specified.

Reviewer #1: **No:**  I didnt see a github link for code

Reviewer #2: **No:**  Code not available. Raw or processed data also seems not available except on the plots

Reviewer #3: **No:**  There is no associated github or Zonodo repository for this paper, nor supplemental files. Anyone wishing to use this model would have to program it themselves from scratch and take the authors’ parameterization as accurate because it cannot be checked against the data. This is surprising as the modeling is done in MATLAB, so these could easily be provided.

**********

PLOS authors have the option to publish the peer review history of their article (what does this mean?). If published, this will include your full peer review and any attached files.

If you choose “no”, your identity will remain anonymous but your review may still be made public.

**Do you want your identity to be public for this peer review?**  For information about this choice, including consent withdrawal, please see our Privacy Policy.

Reviewer #1: No

Reviewer #2: No

Reviewer #3: No

[NOTE: If reviewer comments were submitted as an attachment file, they will be attached to this email and accessible via the submission site. Please log into your account, locate the manuscript record, and check for the action link "View Attachments". If this link does not appear, there are no attachment files.]

**Figure resubmission:**

After uploading your figures to PLOS’s NAAS tool - https://ngplosjournals.pagemajik.ai/artanalysis, NAAS will process the files provided and display the results in the "Uploaded Files" section of the page as the processing is complete. If the uploaded figures meet our requirements (or NAAS is able to fix the files to meet our requirements), the figure will be marked as "fixed" above. If NAAS is unable to fix the files, a red "failed" label will appear above. When NAAS has confirmed that the figure files meet our requirements, please download the file via the download option, and include these NAAS processed figure files when submitting your revised manuscript.

**Reproducibility:**

To enhance the reproducibility of your results, we recommend that authors of applicable studies deposit laboratory protocols in protocols.io, where a protocol can be assigned its own identifier (DOI) such that it can be cited independently in the future. Additionally, PLOS ONE offers an option to publish peer-reviewed clinical study protocols. Read more information on sharing protocols at https://plos.org/protocols?utm_medium=editorial-email&utm_source=authorletters&utm_campaign=protocols

Attachment Submitted filename: Review_PCOMPBIOL-D-26-00654.docx

# Author comment: Author response to Decision Letter 1

**DOI:** 10.1371/journal.pcbi.1014759.r002

3 Jun 2026

Attachment Submitted filename: 2026_PLOS_coculture_rebuttal_final2.pdf

# Peer review: Decision Letter 1

**DOI:** 10.1371/journal.pcbi.1014759.r003

**Authors:** Marc R Birtwistle, Rajib Saha

20 Jul 2026

PCOMPBIOL-D-26-00654R1

From observable fermentation data to hidden cell states: A modeling study of a mixotrophic *Clostridium*  coculture under perfusion mode

PLOS Computational Biology

Dear Dr. Kwon,

Thank you for submitting your manuscript to PLOS Computational Biology. After careful consideration, we feel that it has merit but does not fully meet PLOS Computational Biology's publication criteria as it currently stands. Therefore, we invite you to submit a revised version of the manuscript that addresses the points raised during the review process.

Please submit your revised manuscript by Aug 14. If you will need more time than this to complete your revisions, please reply to this message or contact the journal office at ploscompbiol@plos.org. When you're ready to submit your revision, log on to https://www.editorialmanager.com/pcompbiol/ and select the 'Submissions Needing Revision' folder to locate your manuscript file.

Please include the following items when submitting your revised manuscript:

* A letter that responds to each point raised by the editor and reviewer(s). You should upload this letter as a separate file labeled 'Response to Reviewers'. This file does not need to include responses to formatting updates and technical items listed in the 'Journal Requirements' section below.

* A marked-up copy of your manuscript that highlights changes made to the original version. You should upload this as a separate file labeled 'Revised Manuscript with Track Changes'.

* An unmarked version of your revised paper without tracked changes. You should upload this as a separate file labeled 'Manuscript'.

If you would like to make changes to your financial disclosure, competing interests statement, or data availability statement, please make these updates within the submission form at the time of resubmission. Guidelines for resubmitting your figure files are available below the reviewer comments at the end of this letter.

As the corresponding author, your ORCID iD is verified in the submission system and will appear in the published article. PLOS supports the use of ORCID, and we encourage all coauthors to register for an ORCID iD and use it as well. Please encourage your coauthors to verify their ORCID iD within the submission system before final acceptance, as unverified ORCID iDs will not appear in the published article. Only the individual author can complete the verification step; PLOS staff cannot verify ORCID iDs on behalf of authors.

We look forward to receiving your revised manuscript.

Kind regards,

Rajib Saha, Ph.D.

Academic Editor

PLOS Computational Biology

Marc Birtwistle

Section Editor

PLOS Computational Biology

**Additional Editor Comments (if provided):**

**Reviewers' comments:**

Reviewer's Responses to Questions

**Comments to the Authors:**

**Please note here if the review is uploaded as an attachment.**

Reviewer #1: The authors have addressed all my concerns adequately.

Reviewer #2: The authors have satisfactorily addressed most questions and issues. Just a few minor comments and clarifications needed:

- About how the rRNA-FISH + flow cytometry data was used, in the revised text, it reads:

- 'rRNA-FISH/flow cytometry measurements were used to constrain the active populations of Cac and Clj. Thus, XCac and XClj represent experimentally constrained active biomass-equivalent concentrations, whereas XC∗ac and XC∗lj represent model-reconstructed dormant biomass-equivalent latent states on the same scale.'

- 'rRNA-FISH detects ribosome-rich, translationally active cells rather than the entire community'

- '... whereas the active cell population was obtained from rRNA-FISH/flow cytometry measurements'

- '... while rRNA-FISH/flow cytometry measurements provided active cell population information'

There is a missing link from the rRNA-FISH + flow cytometry described in the Methods section. The output from flow cytometry is probably a distribution of fluorescence intensity. How exactly was the data used in the model? Was fluorescence intensity data processed to get a percentage of metabolically active cells in each sample, followed by multiplying by the total biomass unit converted from OD 600 to estimate XCac and XClj? Please clarify how exactly the experimental data was used to estimate/fit variables/parameters.

- The model complexity vs. performance comparison is an excellent addition. Models M0 - M3 probably refer to what features in Table 2 are available for SINDy but they are not exactly clear from the new supplementary information file (section 1.5 and Figure E). For example, does M0 mean theta(x) = 0 or a constant? Does M1 include pH-dependent inhibition term because of acid dissociation? Does M2 include additionally active biomass only or also biomass-coupled growth terms which also include active biomass so the growth-coupled terms are not in M0 and M1? Maybe put an extended form of Table 2 in the SI file with a new column specifying which models tested include the feature.

- An interesting observation is that the M2 active-biomass-informed model already drastically improved the SINDy results while the M3 full model further significantly improved the SINDy results. So for practical modeling point of view, if rRNA-FISH/flow cytometry data is not available (which is probably the case in many other studies), would the M2 form of model with SINDy still be applicable and get reasonably good results, say, only with biomass estimated from OD600? Or the active vs. dormant population dynamics, which I think was kept in all these tested models here, is pretty much still largely needed for the good performance of the M2 model? This is probably something that people who want to model their fermentation data are interested to know.

Reviewer #3: Manuscript is significantly improved, and all concerns from this reviewer are addressed.

**********

**Have the authors made all data and (if applicable) computational code underlying the findings in their manuscript fully available?**

The PLOS Data policy requires authors to make all data and code underlying the findings described in their manuscript fully available without restriction, with rare exception (please refer to the Data Availability Statement in the manuscript PDF file). The data and code should be provided as part of the manuscript or its supporting information, or deposited to a public repository. For example, in addition to summary statistics, the data points behind means, medians and variance measures should be available. If there are restrictions on publicly sharing data or code —e.g. participant privacy or use of data from a third party—those must be specified.

Reviewer #1: Yes

Reviewer #2: Yes

Reviewer #3: Yes

**********

PLOS authors have the option to publish the peer review history of their article (what does this mean?). If published, this will include your full peer review and any attached files.

If you choose “no”, your identity will remain anonymous but your review may still be made public.

**Do you want your identity to be public for this peer review?**  For information about this choice, including consent withdrawal, please see our Privacy Policy.

Reviewer #1: No

Reviewer #2: No

Reviewer #3: No

[NOTE: If reviewer comments were submitted as an attachment file, they will be attached to this email and accessible via the submission site. Please log into your account, locate the manuscript record, and check for the action link "View Attachments". If this link does not appear, there are no attachment files.]

**Figure resubmission:**

**Reproducibility:**

To enhance the reproducibility of your results, we recommend that authors of applicable studies deposit laboratory protocols in protocols.io, where a protocol can be assigned its own identifier (DOI) such that it can be cited independently in the future. Additionally, PLOS ONE offers an option to publish peer-reviewed clinical study protocols. Read more information on sharing protocols at https://plos.org/protocols?utm_medium=editorial-email&utm_source=authorletters&utm_campaign=protocols

# Author comment: Author response to Decision Letter 2

**DOI:** 10.1371/journal.pcbi.1014759.r004

22 Jul 2026

Attachment Submitted filename: 2026_PLOS_coculture_revision2_rebuttal.pdf

# Peer review: Decision Letter 2

**DOI:** 10.1371/journal.pcbi.1014759.r005

**Authors:** Marc R Birtwistle, Rajib Saha

25 Aug 2026

Dear Dr. Kwon,

We are pleased to inform you that your manuscript 'From observable fermentation data to hidden cell states: A modeling study of a mixotrophic *Clostridium*  coculture under perfusion mode' has been provisionally accepted for publication in PLOS Computational Biology.

Before your manuscript can be formally accepted you will need to complete some formatting changes, which you will receive in a follow up email. A member of our team will be in touch with a set of requests.

Please note that your manuscript will not be scheduled for publication until you have made the required changes, so a swift response is appreciated.

IMPORTANT: The editorial review process is now complete. PLOS will only permit corrections to spelling, formatting or significant scientific errors from this point onwards. Requests for major changes, or any which affect the scientific understanding of your work, will cause delays to the publication date of your manuscript.

Should you, your institution's press office or the journal office choose to press release your paper, you will automatically be opted out of early publication. We ask that you notify us now if you or your institution is planning to press release the article. All press must be co-ordinated with PLOS.

Thank you again for supporting Open Access publishing; we are looking forward to publishing your work in PLOS Computational Biology.

Best regards,

Rajib Saha, Ph.D.

Academic Editor

PLOS Computational Biology

Marc Birtwistle

Section Editor

PLOS Computational Biology

***********************************************************

Reviewer's Responses to Questions

**Comments to the Authors:**

**Please note here if the review is uploaded as an attachment.**

Reviewer #2: All previous comments have been addressed satisfactorily.

**********

**Have the authors made all data and (if applicable) computational code underlying the findings in their manuscript fully available?**

The PLOS Data policy requires authors to make all data and code underlying the findings described in their manuscript fully available without restriction, with rare exception (please refer to the Data Availability Statement in the manuscript PDF file). The data and code should be provided as part of the manuscript or its supporting information, or deposited to a public repository. For example, in addition to summary statistics, the data points behind means, medians and variance measures should be available. If there are restrictions on publicly sharing data or code —e.g. participant privacy or use of data from a third party—those must be specified.

Reviewer #2: Yes

**********

PLOS authors have the option to publish the peer review history of their article (what does this mean?). If published, this will include your full peer review and any attached files.

If you choose “no”, your identity will remain anonymous but your review may still be made public.

**Do you want your identity to be public for this peer review?**  For information about this choice, including consent withdrawal, please see our Privacy Policy.

Reviewer #2: No

# Editor report: Acceptance letter

**DOI:** 10.1371/journal.pcbi.1014759.r006

**Authors:** Marc R Birtwistle, Rajib Saha

PCOMPBIOL-D-26-00654R2

From observable fermentation data to hidden cell states: A modeling study of a mixotrophic *Clostridium*  coculture under perfusion mode

Dear Dr Kwon,

I am pleased to inform you that your manuscript has been formally accepted for publication in PLOS Computational Biology. Your manuscript is now with our production department and you will be notified of the publication date in due course.

The corresponding author will soon be receiving a typeset proof for review, to ensure errors have not been introduced during production. Please review the PDF proof of your manuscript carefully, as this is the last chance to correct any errors. Please note that major changes, or those which affect the scientific understanding of the work, will likely cause delays to the publication date of your manuscript.

Soon after your final files are uploaded, unless you have opted out, the early version of your manuscript will be published online. The date of the early version will be your article's publication date. The final article will be published to the same URL, and all versions of the paper will be accessible to readers.

For Research, Software, and Methods articles, you will receive an invoice from PLOS for your publication fee after your manuscript has reached the completed accept phase. If you receive an email requesting payment before acceptance or for any other service, this may be a phishing scheme. Learn how to identify phishing emails and protect your accounts at https://explore.plos.org/phishing.

Thank you again for supporting PLOS Computational Biology and open-access publishing. We are looking forward to publishing your work!

With kind regards,

Janani Seenivasan

PLOS Computational Biology | Carlyle House, Carlyle Road, Cambridge CB4 3DN | United Kingdom ploscompbiol@plos.org | Phone +44 (0) 1223-442824 | ploscompbiol.org | @PLOSCompBiol
